# Tubulin Tyrosine Ligase Like 12, a TTLL Family Member with SET- and TTL-Like Domains and Roles in Histone and Tubulin Modifications and Mitosis

**DOI:** 10.1371/journal.pone.0051258

**Published:** 2012-12-12

**Authors:** Jan Brants, Kostyantyn Semenchenko, Christine Wasylyk, Aude Robert, Annaick Carles, Alberto Zambrano, Karine Pradeau-Aubreton, Catherine Birck, Jack A. Schalken, Olivier Poch, Jan de Mey, Bohdan Wasylyk

**Affiliations:** 1 Institut de Génétique et de Biologie Moléculaire et Cellulaire, UMR 7104 CNRS UDS - U 964 INSERM , Illkirch, France; 2 Université de Strasbourg, Ecole Supérieure de Biotechnologie de Strasbourg C.N.R.S. - U.M.R.7100, Equipe “Microtubules et Morphogenèse”, Parc d'Innovation, Illkirch, France; 3 Department of Urology, Radboud University Nijmegen Medical Centre, Nijmegen, The Netherlands; Ludwig-Maximilians-Universität München, Germany

## Abstract

hTTLL12 is a member of the tubulin tyrosine ligase (TTL) family that is highly conserved in phylogeny. It has both SET-like and TTL-like domains, suggesting that it could have histone methylation and tubulin tyrosine ligase activities. Altered expression of hTTLL12 in human cells leads to specific changes in H4K20 trimethylation, and tubulin detyrosination, hTTLL12 does not catalyse histone methylation or tubulin tyrosination in vitro, as might be expected from the lack of critical amino acids in its SET-like and TTLL-like domains. hTTLL12 misexpression increases mitotic duration and chromosome numbers. These results suggest that hTTLL12 has non-catalytic functions related to tubulin and histone modification, which could be linked to its effects on mitosis and chromosome number stability.

## Introduction

The human genome codes for may proteins that have not been assigned a validated function. In our screens of RNAs that are differentially expressed in tumours, we identified a number of encoded proteins with unknown functions that could potentially be targeted for therapeutic intervention [1]. We selected hTTLL12 for further study, since it has enzymic features. We showed that hTTLL12 is expressed in the proliferating layer of benign human prostate, and expression increases during cancer progression to metastasis. Overexpression alters chromosomal ploidy. These results raise the possibility that hTTLL12 could contribute to tumorigenesis through effects on chromosome number stability [2]. In order to study whether hTTLL12 may have enzymatic activity, we used sequence homology searches to reveal the presence of SET-like and TTL-like domains in the N- and C-terminal parts of the molecule, respectively.

SET domains are approximately 130 amino acids long and have been found in all eukaryotic organisms studied so far. Their principle function is to transfer a methyl group from S-adenosyl-L-methionine (SAM) to the ε-amino group of lysine residues on histones or other proteins. Various histone lysine residues are methylated, and the combination of these methylations and other covalent modifications constitutes “the histone code” that has epigenetic functions and regulates various cellular processes, such as transcription and the organization of chromatin. Chromatin condensation and compaction are essential for rapid chromosome congression and accurate chromosome segregation during cell division [reviews: [3], [4], [5], [6], [7]].

TTL domains are approximately 350 amino acid modules that catalyze ligation of amino acids to tubulins or other substrates. The TTL domain contains ATP-grasp-like motifs that correspond to the ATP/Mg^2+^ binding site typical of enzymes with ATP-dependent carboxylate-amine/thiol ligase activity [8]. This domain is present in a family of proteins that has 14 members in mouse. They have been shown to ligate tyrosine (TTL), glutamate (TTLL1, 4, 5, 6, 7, 9, 11 and 13) or glycine (TTLL3, 8 and 10) to the C-terminal tails of α/β tubulin. TTL re-adds tyrosine to α-tubulin that has been terminally detyrosinated, in a process called the TTL cycle. TTLL1, 4, 5 and 7 ligate an initial glutamate to a glutamic acid side chain through iso-peptide bonds, whereas TTLL 6, 7, 9, 11 and 13 elongate polyglutamate chains through peptide bonds. In related reactions, TTLL3 and 8 ligate glycine to a glutamic acid side chain, whereas TTLL3 and 10 elongate polyglycine chains. Tubulin C-terminal tails are hotspots for complex patterns of modifications, with important roles in cellular processes that include subcellular organization, intra-cellular transport, cell movement and mitosis [reviews: [9], [10], [11], [12], [13]]. The complexity and importance of tubulin modifications has led to the analogy being made between the “tubulin code” and the better established “histone code” [14], [15].

hTTLL12 is the least characterized and most unusual member of the TTL family. We report its effects on histone and tubulin modifications, mitotic duration and chromosome numbers. hTTLL12 does not appear to have detectable in-vitro enzymatic activity related to the changes observed in cells. We raise the possibility that hTTLL12 is an inactive pseudo-enzyme that has important regulatory roles, similar to pseudo-enzymes in other protein families.

## Materials and Methods

### Materials

Details for vector constructs, siRNAs, antibody generation, commercial antibodies and their dilutions used for western blotting (WB) and immunocytochemistry, are described in Supporting Information.

### Databases searches

The initial step was an iterated PSI-BLAST search with the full-length human hTTLL12 CDS against the nr database of NCBI until convergence occurred. Similar PSI-BLAST searches were performed using the C-terminal TTL-like domain and the N-terminal unknown region [16].

### Multiple sequence alignments and phylogenetic trees

For each domain or region, the detected homologs were included in a clustered multiple alignment of complete sequences (MACS) constructed using the PipeAlign program suite [17] and subsequently manually adjusted. For the MACS of the N-terminal of hTTLL12, SET domain containing proteins were retrieved from PFAM (PF00856). Human SET domain proteins were retained and aligned with the hTTLL12 sequence through ClustalX and manually adjusted. The MACS of the TTL domain was made using representative sequences for each human TTL-like and TTL protein. All MACSs and details about the proteins included are available in data. The PhyloWin program [18] was used to generate the phylogenetic tree based on 212 positions of the TTL domain MACS and using the neighbour-joining reconstruction algorithm with pair-wise gap removal option and 1000 bootstrap replicates. The trees were edited and displayed with TreeView [19].

### Cell culture, transfection and generation of stable clones

HEp-2 cells (ATCC CCL-23) were grown in modified Eagle's medium (MEM), 10% foetal calf serum, 0.1 mM nonessential amino acids, 1 mM Na-pyruvate, 40 µg/ml gentamicin at 37°C in a humidified 5% CO_2_ atmosphere. siRNA transfections were performed using Lipofectamine (Invitrogen) according to the manufacturer's protocol. In brief, 100,000 cells per well were seeded in a 12 well plate, grown for 18 hours followed by 3 hours OPTIMEM incubation and transfection during 6 hours. After transfection, medium was changed to normal growth medium and incubated as indicated until cell lysis. To obtain stable clones, HEp-2 cells were transfected with the Flag-*hTTLL12* expression plasmid, the empty pSG5-puro-Flag plasmid [20] or the as*hTTLL12* expression plasmid using the BBS calcium phosphate method [21]. 48 hours post-transfection, cells were passaged into selection medium containing 3 µg/ml puromycin, renewed every three days. 10–14 days post-transfection, we characterized five positive clones per stable transfection by immunofluorescence and WB giving rise to hTTLL12 clones A-E and Control clones A–E. Since we did not observe significant difference between as*hTTLL12* clones and empty vector clones by various criteria we considered the ashTTLL12 clones as additional negative controls (Control clones F–G).

### SDS-PAGE and WB

Standard procedures were used (see Supporting Experimental Procedures S1 for details).

### Co-immunoprecipitation

Nuclear extracts from hTTLL12_A, _B and _C clones were prepared using the Nuclear Complex Co-IP Kit (Active Motif) according to the manufacturer's instructions. For tubulin co-immunoprecipitation, 75% confluent hTTLL12 or control clones were washed thrice with cold 1× PBS, lysed in RIPA buffer (30 minutes, ice), scraped and cleared (12,000 g, 2 minutes). After protein quantification of the supernatants (Bradford assay, BioRad), 1 mg of proteins was pre-cleared with 100 µl of protein A-Sepharose (Sigma-Aldrich, 30 minutes, 4°C). After centrifugation, supernatants were mixed to 100 µl of M2-sepharose (Sigma-Aldrich) and incubated for 90 minutes at 4°C. Beads were washed thrice, bound proteins were eluted by boiling in 100 µl 2× SDS sample buffer (10 minutes) and centrifuged (10,000 g, 30 seconds). The resulting supernatants were fractionated on a 8.5% SDS-PAGE gel and WB was performed as described above.

### Immunocytochemistry and Confocal Microscopy

Standard procedures were used (see Supporting Experimental Procedures S1 for details).

### Cell cycle synchronisation by double thymidine block

Cells were plated on 10 cm dishes in an amount (≈750,000) that would give 80% confluence in 4–5 days. After about 24 h after plating, medium with 0.2 µM thymidine was applied for 23 h, then changed for fresh medium for 9 h, and again with thymidine for 23 h. Cells were collected at 2 h time intervals for 18–24 h for FACS analysis for DNA content. Time points in which cells were predominantly in the S1, G2/M and G1 phase were selected for WBs.

### Time-lapse microscopy

Control clones, hTTLL12 clones or parental HEp-2 cells were seeded at a density of 200,000 cells/2 ml in six well cell culture plates. siRNA transfection of parental HEp-2 cells was performed as described above. 48 hours after seeding of the stable clones, or 72 hours after siRNA transfection [3 non-targeting control siRNAs (siScramble, siLuciferase and siCtrl), or one of 6 hTTLL12-sepecific siRNAs (sihTTLL12_1-6)], cell cultures were stained with vital Hoechst (3.3 µg/ml, Hoechst 33258, Molecular Probes) during 30 minutes at 37°C and grown on a video microscopy platform in a humidified 5% CO_2_ atmosphere. Images were acquired in phase contrast and UV at 1 minute intervals during 4 hours (Control and hTTLL12 clones) or 3 minutes intervals during 10 hours (siRNA transfected cells) using a CCD camera with a 40×//0.55 N-Plan objective in an inverted motorized microscope (DMIRE2, Leica). Analyses were performed using ImageJ software (http://rsb.info.nih.gov/ij/). Mitotic duration was scored from cells that had one, normal-sized, nucleus. The following numbers of cells were used to analyse: (a) mitotic duration, 205 (HEp-2), 263 (Control clones), 372 (hTTLL12 clones), 591 (siControl transfected Hep-2), and 899 (sihTTLL12 transfected Hep-2); (b) spindle positioning, 262 (HEp-2), 420 (Control clones), 380 (hTTLL12 clones), 502 (siControl transfected Hep-2), and 973 (sihTTLL12 transfected Hep-2); (c) congression delay, 420 (Control clones), 491 (hTTLL12 clones), 502 (siControl transfected Hep-2), and 852 (sihTTLL12 transfected Hep-2).

### Cell growth

3,000 cells of hTTLL12 and Control clones (hTTLL12_A-E and Control_A-E) were seeded per well of a 96 well plate. Cell proliferation was assayed using the MTT-based colorimetric assay (Chemicon Int.) following the manufacturer's instructions.

### Cell cycle analysis

Unsynchronized exponentially growing cells were fixed in 70% ethanol (−20°C), washed and stained with propidium iodide. Detached cell populations were collected and combined with adherent cells for all experiments. FACScalibur cytometer (Becton Dickinson) and Cellquest software were used for acquisition, and Modifit software (Verity) for cell cycle analysis.

### In-vitro assays for histone methyl transferase and tubulin tyrosine ligase and purification of proteins

Standard procedures were used as detailed in the Experimental Procedures.

### Statistics

Statistical analyses were carried out by Student's *t*-test.

## Results

### Domain structure and phylogenetic distribution of hTTLL12

**Figure 1:**
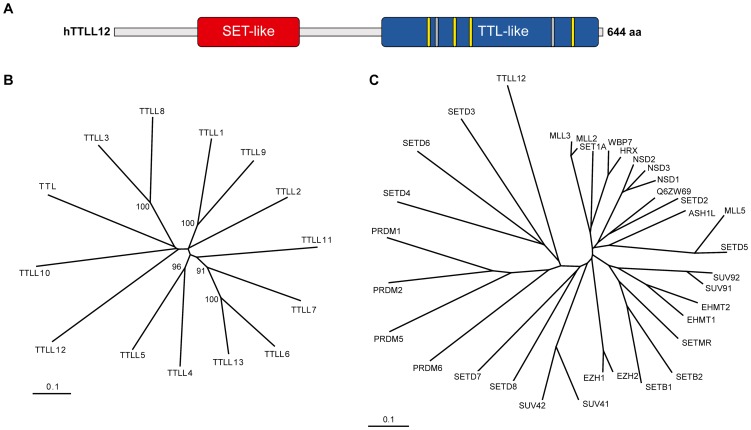
hTTLL12, domain organisation and similarity with other proteins. *A.* Schematic representation. Red indicates the SET-like domain (amino acids 91–249), blue the TTL-like domain (amino acids 353–642), yellow or grey the predicted ATP and Mg^2+^ binding amino acid motifs that are respectively conserved (WICK^416–419^, SKYI^450–453^, DIRY^470–473^, EVN^605–607^), or non-conserved (SLDT^426–429^ and RAMYAVD^578–584^) in hTTLL12. *B, C.* Phylogenetic trees of the TTL domains of the human TTL family based on Alignment S1 *(B)* and the SET-like domain of hTTLL12 and human SET domains based on Alignment S2 *(C)*. Bootstrap values are provided for significant nodes when they are >80%. Multiple sequence alignments are available as data.

We initially characterised human hTTLL12 using bioinformatics. There are thirteen human proteins with homology to the hTTLL12 C-terminal TTL domain (Figure 1A, Alignment S1) [22], [23]. The phylogenetic tree, based on the sequences of the TTL domains (Figure 1B), has a relatively long branch for hTTLL12 compared to the other hTTL-like proteins, indicating that it is one of the most divergent. The core TTL domain (416–642) of hTTLL12 has a N-terminal extension (353–415) with weak homology to the extended domains of the other hTTL family members (Alignment S1 and data not shown). Despite the overall similarity, the TTL domain of hTTLL12 has distinct features. It lacks 2 of the six sequences predicted to bind ATP and Mg^2+^ in the other TTL domains (Figure 1A, grey lines). It also lacks three of the seven conserved motifs found in the other TTL family members (Table S1). In addition, it contains specific residues that are conserved in TTLL12s from other species (Table S1). Interestingly, TTLL12 is widely conserved; it is found in simple eukaryotes (Entamoeba, Trypanosomes, Paramecium, Tetrahymena, Hydra), plants [monocots (*Oryza sativa*, Indian rice), eudicots (*Arabidopsis thaliana*)], nematodes (*Caenorhabditis elegans*, etc.), flies (*Drosophila melanogaster*, etc.) and higher eukaryotes (data not shown). The conserved sequences include the N-terminal region, which has a SET-like domain according to PSI-BLAST [16] (amino acids 91 to 249 of hTTLL12; Figure 1A). This sequence aligns with human SET domain proteins from PFAM (Alignment S2), and is most related to the SET domains of SETD3, SETD4 and SETD6 (Figure 1C). Some of its sequences are highly conserved in other human SET domains (see Table S2; letters colours indicate degrees of conservation; red: absolutely conserved; blue: very highly conserved; black: highly conserved). The highly conserved sequences correspond to SET domain structural elements, including β-strands, an α-helix and a linker region [6]. The hTTLL12 SET-like domain appears to contain an insert, as found in iSET domains [24]. This insert lies between amino acids 158–188 (Alignment S2). Several plant and protozoan orthologues have an additional sequence inserted in a position equivalent to 160 of hTTLL12, indicating that these orthologues have an iSET domain with a large insert. In summary, hTTLL12 appears to be an atypical member of the TTL protein family, from the presence of a SET-like domain, the loss of conserved residues found in other TTL family proteins, and the conservation of hTTLL12-specific features throughout many species.

### hTTLL12 selectively affects trimethylation of histone H4K20 in cells

**Figure 2:**
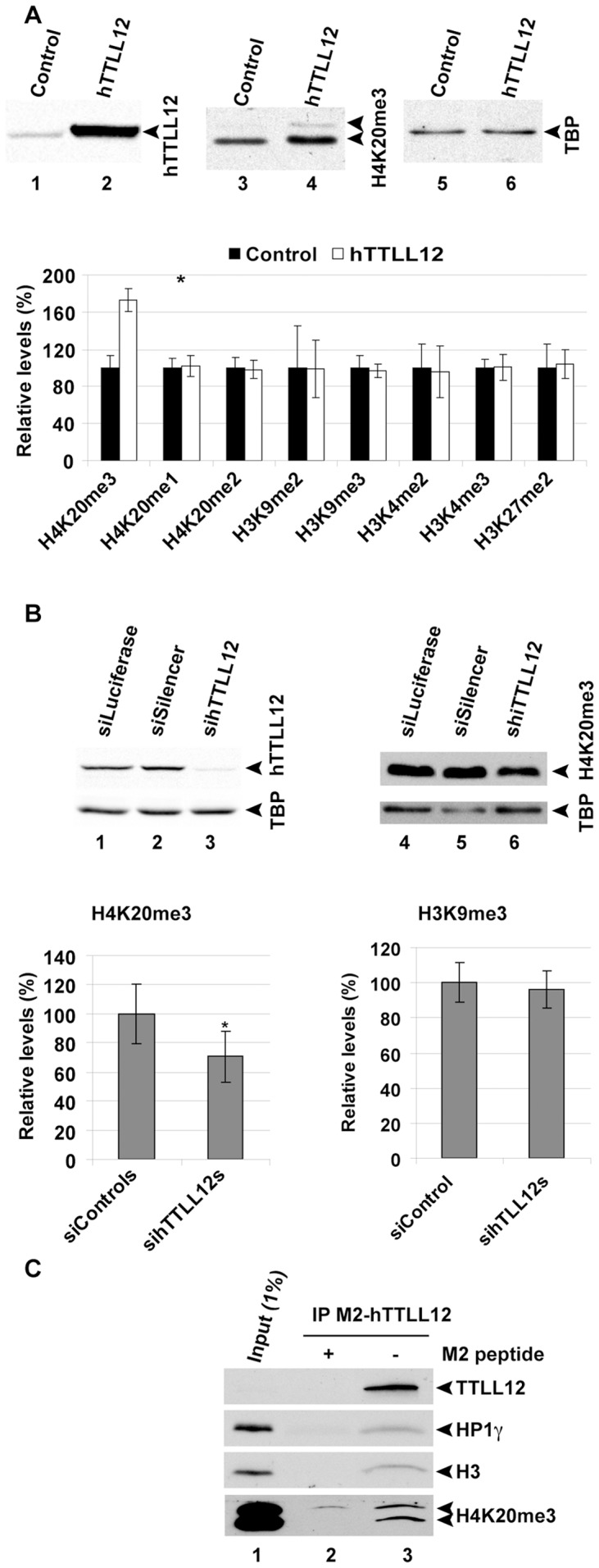
hTTLL12 changes H4K20me3 levels and co-immunoprecipitates with HP1γ, H3 and H4K20me3. *A.* hTTLL12 (hTTLL12_A-E) or Control (Control_A, _B, _D, _E) clones were harvested in Laemmli buffer and subjected to SDS-PAGE and WB. Representative WBs (upper part; hTTLL12_A and Control_E) are shown for hTTLL12 (30 µg protein/lane) or H4K20me3 and TBP (20 µg protein/lane). Similar WBs were probed with different antibodies, quantified by densitometry and normalized to TBP levels (lower part), to determine methylated H4K20me3, H4K20me1, H4K20me2, H3K9me2, H3K9me3, H3K4me2, H3K4me3 and H3K27me2 levels. Data represent average protein levels ± SEM in hTTLL12 lysates relative to average levels in Control lysates (n = 2 except for H4K20me3 (n = 5) and H3K9me3 (n = 3)). *B.* HEp-2 cells were transfected with 10 nM control siRNAs (siLuciferase, siCtrl, siGFP or siSilencer) or 10 nM hTTLL12-specific siRNA (sihTTLL12_2-5). 72 hours after transfection, cells were lysed in Laemmli buffer and analysed by SDS-PAGE and WB. Chemiluminescent signals were detected using a Versadoc image station (hTTLL12) or film (H4K20me3). Representative WBs (upper part) for hTTLL12 (40 µg of total protein/lane), H4K20me3 (5 µg total protein/lane) and respective TBP signals in siLuciferase, siSilencer and sihTTLL12_2 lysates. H4K20me3 levels were quantified using Quantity One software and normalized to TBP (lower left part). Data represent average H4K20me3 levels ± SEM in sihTTLL12 lysates relative to average levels in control siRNA lysates (siControl; n = 3). * Statistically significant difference to the levels in siControl (*P*<0.05, Student's *t*-test). To evaluate H3K9me3 levels (lower right part), HEp-2 cells were transfected with non-targeting control siRNA (siGFP or siCtrl) or hTTLL12-specific siRNA (20 nM sihTTLL12_3 or 10 nM sihTTLL12_4). 48 hours after transfection, cells were harvested in Laemmli buffer and subjected to SDS-PAGE and WB. H3K9me3 levels were quantified by densitometry and normalized to TBP. Data represent average protein levels ± SEM in sihTTLL12 lysates relative to average levels in non-targeting siRNA lysates (siControl; n = 2). *C.* WB showing co-immunoprecipitation of HP1γ, Histone H3 and H4K20me3 with M2-Flagged hTTLL12 from nuclear extract of stable clone hTTLL12_A. Similar results were obtained with two other clones (hTTLL12_B and hTTLL12_C, data not shown). Lane 1: 1% of the input; lane 2: IP of M2-Flagged hTTLL12 in the presence of 500 µg M2-peptide, lane 3: IP M2-Flagged hTTLL12 in the absence of M2-peptide. * Statistically significant (*P*<0.05, Student's *t*-test) difference to the levels in Control or siControl.

Since SET domain proteins methylate histone lysines [reviewed in [25]], we studied whether hTTLL12 affects methylation of histones H3 and H4. We generated stable clones of HEp-2 that overexpress Flag-hTTLL12 (hTTLL12_A-E), and control clones with the corresponding empty Flag-expression vector (Control_A-E). Western blots (WBs) of total cell extracts were probed with antibodies against hTTLL12, specific methylated forms of H4K20, H3K9, H3K4 and H3K27, and a loading control (TBP); suitably exposed films were quantitated (Figure 2A, see lanes 1–6 for representative blots and the histogram for quantification). hTTLL12 overexpression increased the levels of H4K20 trimethylation (p = 0.005), without affecting total H4 protein levels (data not shown), or any of the other modifications investigated. We then studied knockdown of hTTLL12 with siRNAs. Decreasing hTTLL12 levels decreased H4K20me3 levels (p = 0.029; Figure 2B; a representative WB is shown; the histogram combines the results from four sihTTLL12 and four control siRNAs in replicated experiments), whereas H3K9me3 levels were not changed. These results show that up and down regulation of hTTLL12 have opposing effects on H4K20 trimethylation. In order to investigate whether there is a direct connection between hTTLL12 and chromatin, we tested whether hTTLL12 interacts with histones and chromatin components such as HP1 isoforms [26], [27]. We found that immunoprecipitates of Flag-hTTLL12 from nuclear extracts of hTTLL12 overexpressing clones contained HP1γ the nucleosomal component histone H3 and H4K20me3 (Figure 2C). These proteins were not detected in the controls, in which Flag-hTTLL12 pull down was inhibited with the Flag peptide, indicating that the interactions were specific. These results show that hTTLL12 interacts with chromatin components.

### hTTLL12 affects the levels of detyrosinated tubulin

**Figure 3:**
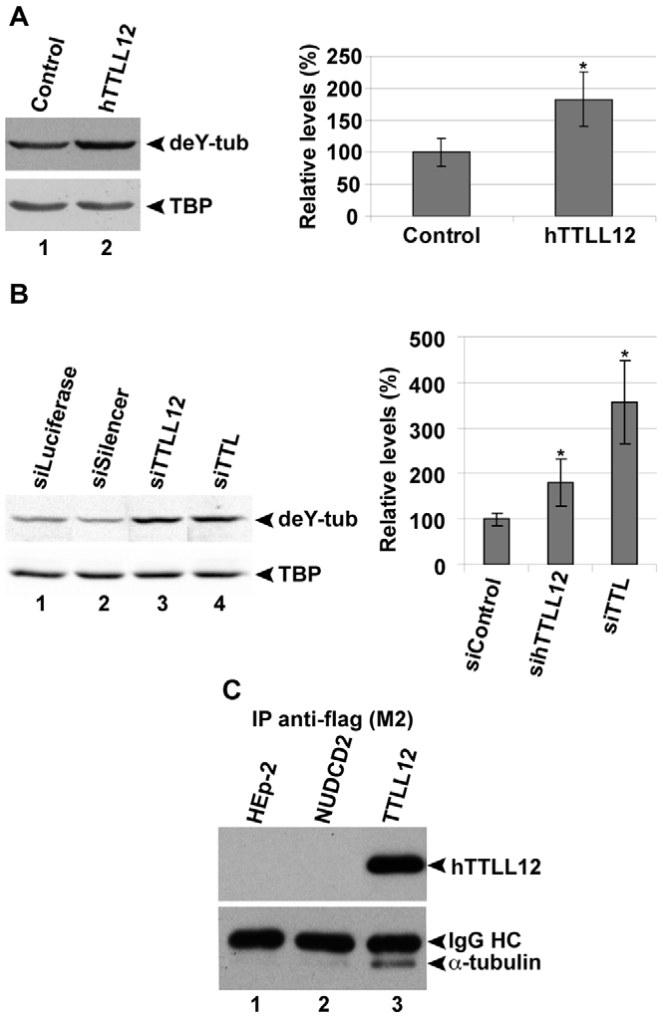
hTTLL12 alters detyrosinated tubulin levels and co-immunoprecipitates with α-tubulin. *A.* hTTLL12 (hTTLL12_A-E) or Control (Control_A, _B, _D, _E) clones were harvested in Laemmli buffer and analysed by SDS-PAGE and WB. Representative WBs (left part) for detyrosinated tubulin (deY-tub) and TBP (40 µg total protein/lane; Control_D and hTTLL12_B shown). deY-tub levels from similar WBs were quantified by densitometry and normalised to TBP (right part). Data represent average detyrosinated tubulin levels ± SEM in hTTLL12 lysates relative to average levels in control lysates (n = 2). * Statistically significant difference to the controls (*P*<0.05, Student's *t*-test). *B.* HEp-2 cells were transfected with 10 nM control siRNAs (siLuciferase, siCtrl, siGFP or siSilencer), 10 nM hTTLL12-specific siRNA (sihTTLL12_1-6), or 10 nM positive control siRNA (siTTL_1-2). 72 hours after transfection, cells were lysed in Laemmli buffer and analysed by SDS-PAGE and WB. Chemiluminescent signals were detected using a Versadoc image station. Representative WBs (left part) for detyrosinated tubulin (40 µg of total protein/lane) and TBP signals (note that lanes 1–3 of the TBP blot are identical to the ones shown in the upper left part of Figure 2B) in siLuciferase, siSilencer, sihTTLL12_2 and siTTL_2 lysates. Detyrosinated tubulin levels were quantified using Quantity One software and normalized to TBP (right part). Data represent average detyrosinated tubulin levels ± SEM in sihTTLL12 and siTTL lysates relative to average levels in control siRNA lysates (siControl; n = 5). * Statistically significant difference to the levels in siControl (*P*<0.05, Student's *t*-test). *C.* Representative WB showing the co-immunoprecipitation of α-tubulin with Flag-hTTLL12 immunoprecipitated from the stable clone hTTLL12_A (lane 3). Similar results were obtained with 4 other clones (hTTLL12_B-E, data not shown). Parental HEp-2 cells (lane1) and a stable HEp2-clone overexpressing Flag-NudCD2 (lane 2) were used as negative controls. IgG HC: IgG heavy chain.

hTTLL12 has a TTL-like domain, suggesting that it might affect tubulin tyrosination. We measured the effects of increasing and decreasing hTTLL12 levels in HEp-2 on detyrosinated tubulin levels by WB with a specific antibody. hTTLL12 overexpression in the stable clones resulted in a significant nearly two-fold increase in detyrosinated tubulin levels, compared to the control clones (p = 0.003; Figure 3A, see the representative WB and the combined data from 4 overexpression and 5 control clones). Knockdown of endogenous hTTLL12 in HEp-2 cells also resulted in a significant increase in detyrosinated tubulin levels (p = 8.8 10^−5^; Figure 3B; a representative WB is shown; the histogram combines the results from six sihTTLL12 and four control siRNAs in replicated experiments). As expected, TTL knockdown also increased detyrosinated tubulin levels, but to a greater extent than knockdown of hTTLL12, even though the extents of knockdown were similar (80% or more, 2 siTTLs and 6 sihTTLL12s; data not shown). We were not able to detect corresponding changes in tyrosinated tubulin, most likely due to the technical limitations of WB and dot-blots. These techniques did not accurately detect the small proportional changes that we would expect to find (about 10%). Hep-2 express a high proportion of tyrosinated tubulin relatively to detyrosinated tubulin (data not shown), which reduces the proportional change of tyrosinated tubulin that would result from conversion to detyrosinated tubulin. Nevertheless, we did find that changes in hTTLL12 levels increase tubulin detyrosination.

The effects of hTTLL12 on tubulin modification may result from interactions with α-tubulin and tubulin-associated structures in the cell. To detect interacting proteins, we immuno-purified Flag-hTTLL12 from overexpressing clones with anti-Flag affinity columns. Proteins that co-purified with Flag-hTTLL12 from overexpressing clones were dissociated by boiling in loading buffer and analysed by WB. α-tubulin was detected in extracts prepared from the Flag-hTTLL12 overexpressing clones (Figure 3C, lane 3, and data not shown), but not from cells that overexpress Flag-NudCD2 (a different protein, lane 2) or from parental cells (lane 1). We also analysed proteins eluted with the FLAG peptide by SDS-PAGE electrophoresis, silver staining and mass spectrometry. One of the bands, that migrated around 50 kDa (the size of α-tubulin) and that was not present in the sample purified from a control clone, was shown to be α-tubulin by mass spectrometry (Figure S1 and Table S3). These results suggest that hTTLL12 interacts specifically with α-tubulin.

We have previously reported that hTTLL12 co-localises with the mitotic spindle during mitosis [2]. We verified that hTTLL12 has a similar location in HEp-2 cells. Cells were fixed, permeabilised and stained with antibodies against hTTLL12 and fluorescently labelled secondary antibodies, and observed by confocal microscopy. The staining was specific, since knockdown with different sihTTLL12s strongly decreased staining intensity (data not shown). hTTLL12 (red; Figure S2) localised to dots on mitotic spindle MTs that co-stained with α-tubulin antibodies (green, Figure S2A). hTTLL12 also appeared to localize to centrosomes, which were co-stained with beta/gamma-tubulin (green; Figure S2B & C). These results indicate that hTTLL12 localises to the tubulin-rich mitotic spindle, which is consistent with its ability to affect tubulin C-terminal modification.

### H4K20me3 and tubulin detyrosination levels are altered by TTLL2 expression at defined stages of the cell cycle

H4K20 methylation and tubulin tyrosination levels change during the cell cycle [28], [29]. hTTLL12 can affect cell cycle distribution in some cell lines [2], suggesting that hTTLL12's effects on histone and tubulin modifications in HEp-2 could be an indirect consequence of changes in cell cycle distribution. We checked whether hTTLL12 expression affects cell cycle progression of HEp-2 cells. Using the MTT assay, we found that hTTLL12 clones grew significantly more slowly from day 6 onwards (Figure S3A). Using WBs of cell extracts, we found that the hTTLL12 clones expressed increased levels of Rb (p = 0.1 10^−3^) and decreased levels of HP1α (p = 0.4 10^−4^), without changes in HP1β and HP1γ (Figure S3B), as expected for inhibition of cell cycle progression by hTTLL12 [30], [31]. FACS analysis showed that hTTLL12 clones also had a larger proportion of cells in G2/M with tetraploid chromosome content (4c; Figure S3C). As observed previously [2], there were also cells with a greater DNA content (8c), as expected from increased chromosome numbers. To measure this increase, we counted chromosomes in metaphase spreads of HEp-2, and hTTLL12 and Control clones (Figure S3C; hTTLL12 red; Control green). The modal chromosome number for HEp-2 (data not shown) and Control clone cells was 74. hTTLL12 overexpressing cells had significantly more cells with greater modal chromosome numbers (p = 4.84 10^−12^). This shows that hTTLL12 overexpression increases the number of chromosomes per cell in HEp-2, similar to HCT-116 cells [2].

**Figure 4:**
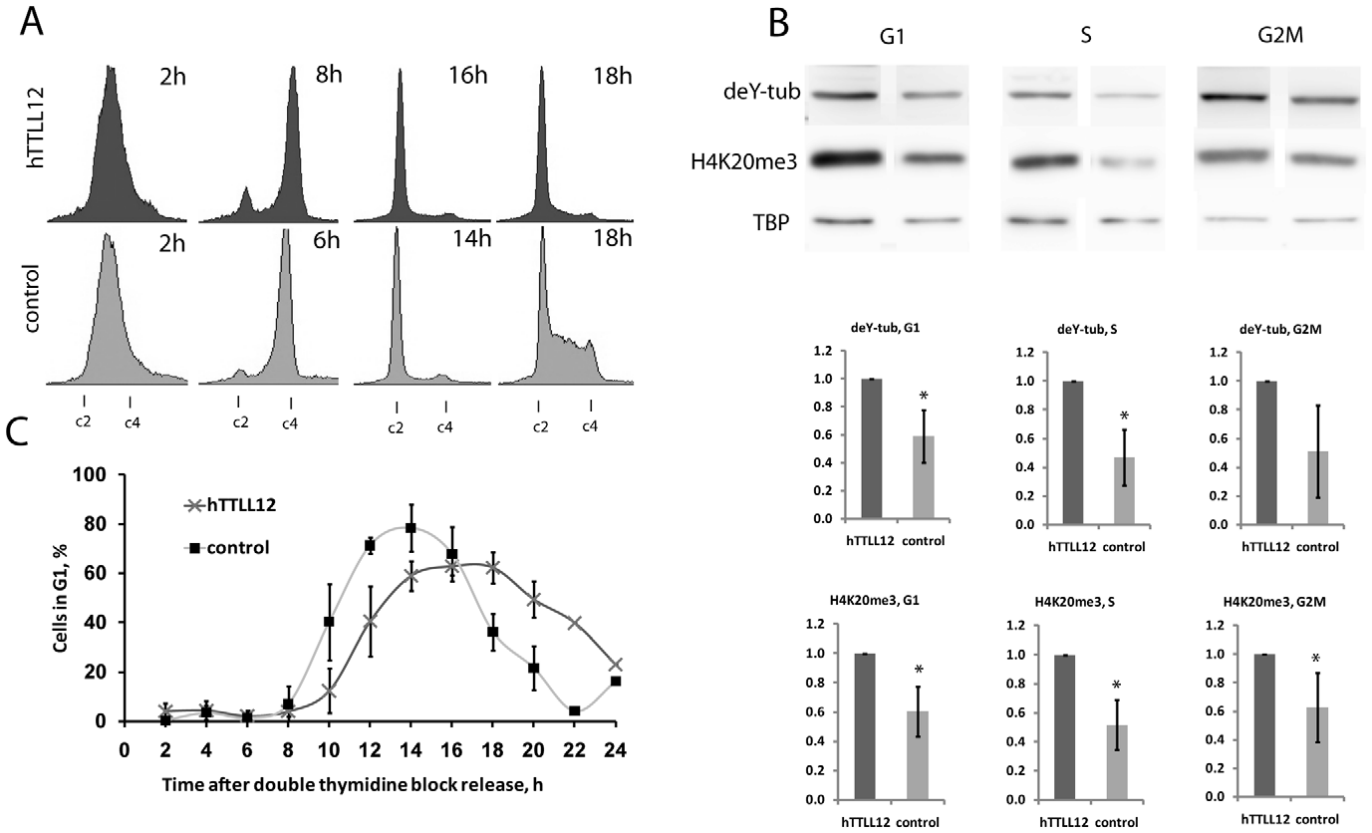
Cell cycle phase dependence of the levels of detyrosinated tubulin (deY-tub) and H4K20me3 in clones overexpressing hTTLL12 compared to control clones. *A.* FACS scans of cells released from a double thymidine block at the indicated times. The times shown are those at which the cells were predominantly in S, G2/M and G1, except for the 18 hour time point. One representative synchronisation is shown. *B.* WBs, from one experiment, of whole cell lysates from cells synchronised in the G1, S and G2/M phases, respectively. The graphs are the averages from 3 independent experiments. * p<0.05. *C.* Proportion of cells in G1 at different times after release from double thymidine block. The average from 3 different experiments (up to 18 h) is shown. c2, c4 = DNA complements as measured by propidium iodide.

Since the difference in H4K20me3 and detyrosinated tubulin levels could have been a consequence of differences in cell cycle distribution of the cell populations that were compared, we studied whether there were differences when cells were at the same stage of the cell cycle. Cells were synchronised by double thymidine block, released, collected every 2 hours for 24 hours, analysed by FACS for DNA content, and time points were selected at which the cells were predominantly in the S, G2/M and G1 phases (Figure 4A). Western blot analysis showed that there were differences in modifications in the selected populations (Figure 4B), suggesting that the differences are not due to differences in cell cycle distributions of the hTTLL12 overexpressing and control clones. Interestingly, we observed that the time to transit G2 and M and then enter into G1 was delayed in the hTTLL12 overexpressing cells (Figure 4C), suggesting that hTTLL12 could have an effect on the length of the G2 or M phases of the cell cycle.

### Mitotic phenotypes induced by hTTLL12 up and down regulation

**Figure 5:**
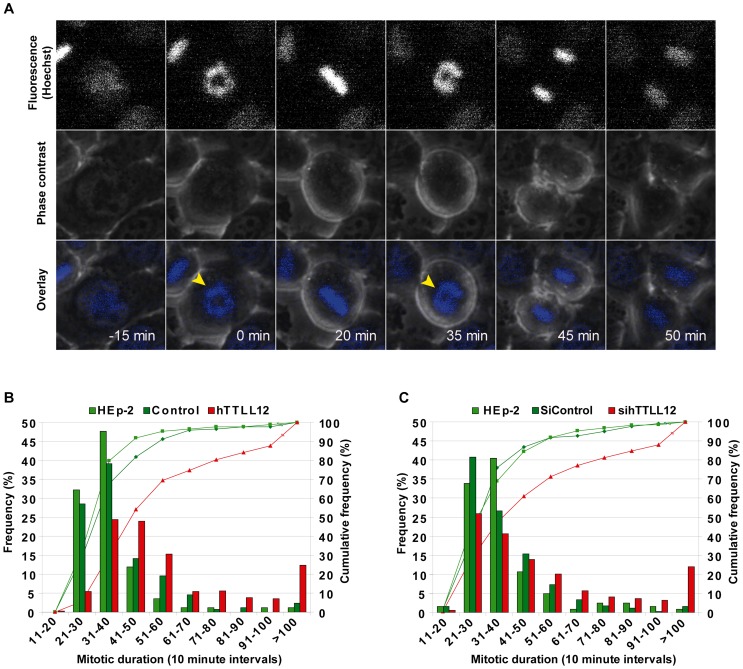
hTTLL12 up and down regulation prolong mitotic duration of HEp-2 cells. *A.* Example frames from live cell movies of a HEp-2 cell stained with vital Hoechst. The time between nuclear envelope breakdown (NEB, first arrowhead, 0 min) and anaphase onset (second arrowhead) are indicated. Upper panel: fluorescent images (Hoechst), middle panel: phase contrast images, lower panel: overlay. *B–C.* Cumulative frequency (plot, right axis) and frequency distribution (histogram, left axis) of mitotic duration for hTTLL12 clones *(B)* and siRNA transfected HEp-2 *(C)*. *B.* hTTLL12 (hTTLL12_A-E) or Control (Control_A-E) clones were seeded in 6-well plates. 48 hours post seeding, cells were treated with vital Hoechst and analysed (Experimental Procedures). Parental HEp-2 cells (light green), and the averages of the hTTLL12 (red) and Control (dark green) clones are plotted. *C.* HEp-2 cells were transfected separately with 12.5 nM sihTTLL12_1-6 or controls (siLuciferase, siCtrl, siScramble). 72 hours post transfection, cells were stained with vital Hoechst and analysed. HEp-2 cells; light green; averages of sihTTLL12s; red; averages of siControls; dark green. More detailed information and statistical analysis can be found in Figure S1.

Microscopy of individual living cells was used to study the effects of altering hTTLL12 levels on the duration of different phases of the cell cycle. Chromosomes were detected by fluorescent labelling with Hoechst vital stain and cells by phase contrast (Figure 5A). Ten clones (5 hTTLL12, 5 Control) and HEp-2 were plated and after 48h the unsynchronized cultures were observed every minute for 4 hours. hTTLL12 overexpressing cells had a longer mitotic duration than Control clones and HEp-2 [61±9 versus 40±4 and 37±1 minutes; Figure 5A, Figure 5B and Figure S5A]. The increased duration of mitosis resulted from increases in both prometaphase and metaphase [hTTLL12 versus Control clones: prometaphase 34±6 versus 25±2 min (p = 0.016), metaphase 27±3 min versus 16±2 min (p = 1.3 10^−4^)]. There were no significant overall differences in other parameters (data not shown), including: (1) chromosome “delay” and “lag” [the presence of chromosomes that did not congress immediately to the metaphase plate (Figure S4A), or that remained stranded at the spindle equator after anaphase, respectively]; (2) chromatin fragmentation and membrane blebbing (markers of cell death), (3) variations in sharpness and centring, and reorientation of the metaphase plate (markers for spindle positioning defects), (4) multipolar spindles (a marker for centrosome duplication and spindle assembly defects); and (5) ingression of the furrow during cytokinesis (a marker for cytokinesis defects). This indicated that the predominant effect of hTTLL12 overexpression is prolongation of prometaphase and metaphase that cumulate to increase mitotic duration.

In parallel, hTTLL12 was depleted from HEp-2 cells with siRNAs (6 sihTTLL12 and 3 controls, used individually; hTTLL12 was decreased by more than 70% 72–80 hours post-transfection). The unsynchronized cultures were observed every 3 minutes for 10 hours. hTTLL12 down regulation increased mitotic duration to 63±15 minutes, compared to 38±2 minutes for the controls (Figure 5C & Figure S4A). About 17% of the hTTLL12 down regulated cells displayed very long mitotic durations, of more than 90 minutes, compared to about 4% of the control cells (Figure S4C). hTTLL12 down regulation did not grossly alter chromosome segregation and cytokinesis compared to the controls. These results show that down regulation as well as up regulation of hTTLL12 had similar effects on mitotic duration. Up and down regulation also had similar effects on the levels of detyrosinated tubulin (see above), suggesting that cells are exquisitely sensitive to hTTLL12 levels.

To facilitate identification of mitotic defects, we focussed on cells with a mitotic duration longer than 50 minutes. Within the hTTLL12 overexpressing cell population, these cells were significantly delayed in chromosome congression to the metaphase plate (Figure S5). They also had differences in spindle positioning prior to anaphase (Figure S6). Control cells displayed a sharp chromosome plate at the equator of the cell very soon after NEB, even before completion of chromosomes congression, indicating that they achieved spindle alignment parallel to the substrate during prometaphase. Modifications of hTTLL12 expression affected spindle positioning, as shown by displacement of the chromosome plate from the cell centre, rotational reorientation of the plate within the cell, and oscillations between a sharp and a fuzzy plate (Figure S6B & C). These are typical characteristics of spindles that are not stably maintained in the cell centre and that change the spatial orientation of their axis. We deduce that hTTLL12 has effects on the spindle that affect congression and positioning, which could affect mitotic timing.

### hTTLL12 lacks HMTase activity in vitro

**Figure 6:**
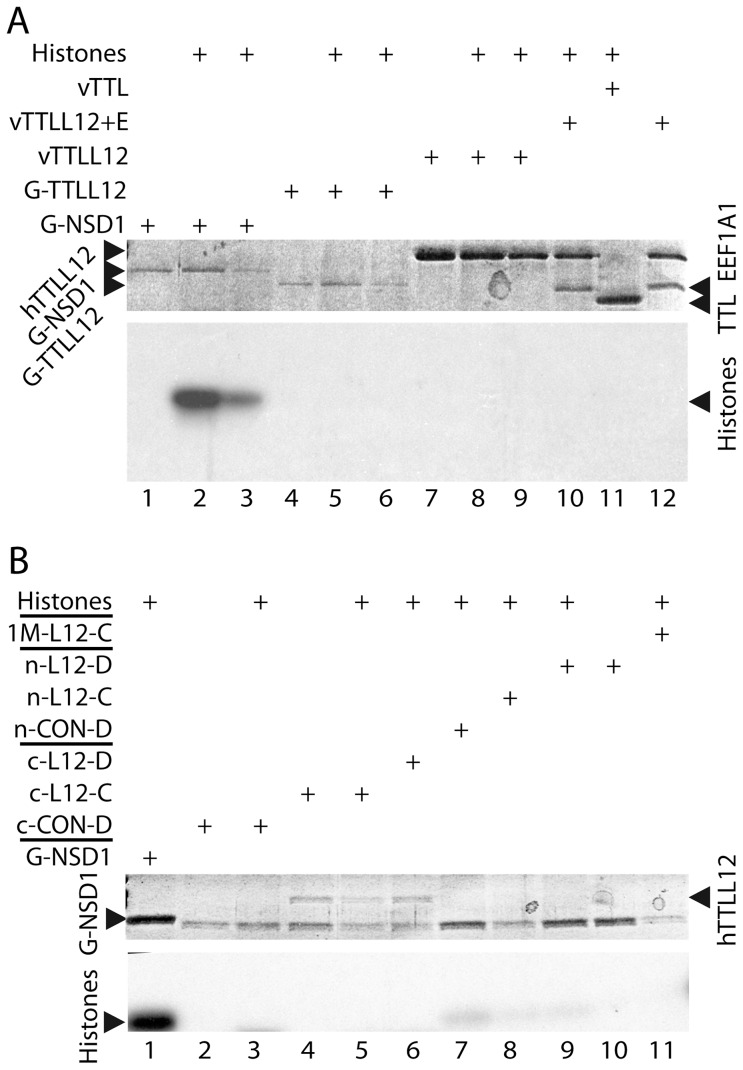
hTTLL12 lacks HMTase activity. The assays included histones purified from calf thymus (*A*, lanes 2, 3,5, 6, 8–11; *B* lanes 1, 3, 5–9, 11) as substrate and proteins to be tested for HMT activity that were purified from bacterial, viral and mammalian cell expression systems. The upper panels in each part (*A* & *B*) are Coomassie Blue stained SDS-polyacrylamide gels of the expressed proteins and the lower panels are the corresponding fluorograms centred on the core histones (Histones). The proteins tested for HMTase activity were expressed and purified from appropriate pGEX vector transformed E. coli [GST-NSD1 (1700–1987) (G-NSD1), GST-TTLL12 (50–250) (G-TTLL12)], recombinant vaccinia virus Ankara strain (MVA) infected mammalian cells [vTTLL12, vTTLL12 and vTTLL12 + E (a fraction that contains EEF1A1)] and HEp-2 cell clones transformed with pSG5-puro-Flag (CON-D) or pSG5-puro-Flag TTLL12 (L12-C & L-12-D). The HEp-2 proteins were purified from cytoplasmic (c-CON-D, c-L12-c, c-L12-D), nuclear (n-CON-D, n-L12-C, n-L12-D) and 1M KCl (1M-L12-C) fractions. 10 µl reactions were loaded on 15% SDS-PAGE gels. In *A*, the approximate amounts of protein used per reaction were: GST-NSD1 (1700–1987) (1 µg, lanes 1, 2; 0.5 µg, lane 3), GST-TTLL12 (50–250) (0.5 µg, lanes 4, 5; 0.25 µg, lane 6), TTLL12 (3 µg, lanes 7, 8; 1.5 µg lane 9), TTLL12 + EEF1A1 (2 µg, lanes 10, 12) and TTL (4 µg, lane 11). In *B*, they were GST-NSD1 (1700–1987) (2 µg, lanes 1, 12), TTLL12 (0.25 µg, lanes 4–6; 0.005 µg lanes 8–10, 0.001 µg lane 11). TTLL12 was not detected in the equivalent fractions purified from the control HEp-2 clone (CON-D). In *B*, lanes 2–11, the band migrating slightly faster than GST-NSD1 (1700–1987)) is IgH from the affinity column that is eluted under the harsh denaturing conditions used for sample preparation for SDS-PAGE.

The presence of a SET-like domain and the effects of hTTLL12 on the levels of H4K20me3 raised the possibility that hTTLL12 could have HMTase activity. In order to investigate this possibility, we used in vitro HMT assays in which the transfer of radiolabelled methyl groups from SAM to purified histones is followed by fluorography. The positive control was NSD1, which has HMTase activity towards both H4K20 and H3K36 [32]. We expressed hTTLL12 in bacteria using the Glutathione S-transferase (GST) Gene Fusion system and in mammalian BHK21 cells using Vaccinia virus Ankara strain (MVA) vectors [33] or establishing stable clones of HEp-2 cells that express Flag-tagged hTTLL12. Tagged proteins were purified by affinity chromatography. The bacterial proteins were essentially pure, whereas the mammalian proteins contained substantial levels of EEF1A1. EEF1A1 was identified by mass spectrometry and WB in both mammalian expression systems, and was shown to co-immunoprecipitate with hTTLL12 (see Figure S1). As expected, we found that a fragment of NSD1 that contains the SET domain [GST-NSD1 (1700–1987)] catalyses the methylation of core histones [Figure 6A; compare lanes 2 & 3 containing different amounts of GST-NSD1 (1700–1987) with lane 1 lacking histones]. In comparison, GST-hTTLL12 (50–250), which contains the SET domain of hTTLL12 and which was purified in parallel with GST-NSD1 (1700–1987), did not have detectable HMTase activity (Figure 6A, lanes 4–6). The same results were obtained in several independent experiments in which the NSD1 and hTTLL12 recombinants were purified in parallel. Similar lack of activity was observed with full length hTTLL12 purified from vaccinia virus infected cells, whether or not the hTTLL12 fraction contained EEF1A1 (Figure 6A, lanes 7–10 and 12). TTL, expressed and purified using the same system, also lacked HMTase activity, as expected from the lack of a SET domain (lane 11). However, this protein was enzymatically active in tubulin tyrosine ligase assays [[33] and see below]. This suggests that the experimental procedure used is compatible with the isolation of enzymatically active proteins. We also purified hTTLL12 from the clones in which endogenous H4K20me3 levels were altered due to hTTLL12 overexpression. Flag-tagged hTTLL12 was purified from fractionated cell extracts from two overexpressing clones (hTTLL12_C & hTTLL12_D). Control clones (Control_C, Control_D) were processed in parallel to give control extracts. We used fractionated cells since hTTLL12 is located in the cytoplasm and the nucleus of cells, in different proportions depending on the cell type [2]. Three fractions were prepared, the cytoplasmic fraction, a nuclear 0.4 M NaCl wash, and a subsequent 1 M salt wash. The relative levels of hTTLL12 in the three fractions were approximately 100∶2∶0.4 in HEp-2. Purified hTTLL12 did not have any additional HMTase activity compared to the control extracts [Figure 6B, lower panel; compare lanes 4, 5 with 3, lanes 8, 9 with 7, and see the lack of detectable incorporation in lane 11; see also the signal for the positive control GST-NSD1 (1700–1987)]. There was background incorporation of radioactivity that was histone dependent (compare lanes 2 and 3, 4 and 5, and 9 and 10). In some cases the background activity appeared to be lower in the hTTLL12 containing extracts, suggesting that the hTTLL12 extracts could contain inhibitors. However, adding hTTLL12 extracts to either GST-NSD1 (1700–1987)] or control extracts did not significantly decrease their activities (data not shown). The results were reproducible, using independent protein preparations (data not shown). Taken together, our results suggest that hTTLL12 does not have HMTase activity.

### hTTLL12 lacks TTL activity in vitro

**Figure 7:**
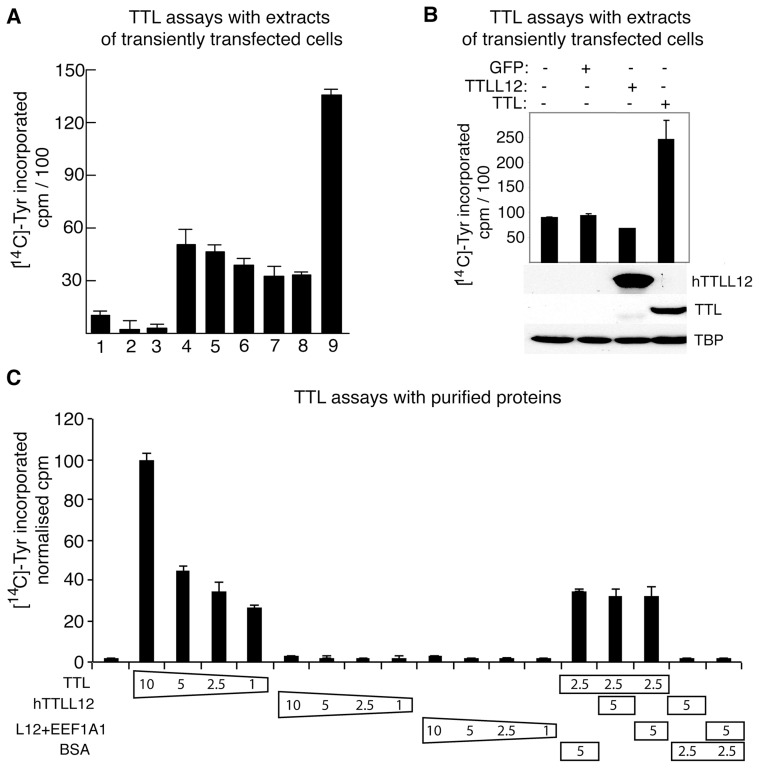
hTTLL12 lacks tubulin tyrosine ligase activity, as shown with tubulin tyrosine ligase (TTL) assays using cell extracts (*A*, *B*) or purified proteins (*C*). (A) TTL assays on extracts of transfected HEp-2 cells. Control reactions lacked cell extract (1), ATP (2) or purified tubulin (3). Test reactions contained extracts from cells transfected with: no DNA (4, mock), pc-AS 153 (5, antisense hTTLL12), pcDNA3-hTTLL12 (6), pSG5-puro-hFlag TTLL12 (7, Flag-hTTLL12), pc-AS TTL (8, antisense hTTL), and pcDNA3-TTL (9). (B) Expression of TTLL12 and TTL in transfected cell extracts. WBs were used to detect hTTLL12, TTL and TBP (loading control) in the extracts used for the activity tests. (C). TTL activity of purified proteins. The in-vitro reactions contained the indicated quantities (µg) of TTL, hTTLL12 and hTTLL12 complexed to EEF1A1 that had been purified from mammalian cells infected with corresponding recombinant Ankara strain (MVA) vaccinia viruses. The counts were normalised to 100 for 10 µg TTL. Error bars indicate standard deviations.

Since hTTLL12 has a TTL-like domain, we studied whether it has TTL activity. We used in vitro assays with [^14^C] labelled tyrosine, purified tubulin, and either extracts from transiently transfected cells or proteins purified from mammalian cells infected with appropriate viral expression vectors. Extracts from cells transfected with the TTL expression vector exhibited increased TTL activity compared to mock-transfected cell extracts (Figure 7A, lanes 9 and 4, respectively). The reactions were specific, as shown by dependence on cell extract (1), ATP (lane 2), tubulin (lane 3) and the use of an expression vector with the appropriate orientation (lane 8). Extracts from cells transfected with expression vectors for hTTLL12 (6) and Flag-hTTLL12 (7) did not increase endogenous TTL activity. As might be expected, transfection of the hTTLL12 anti-sense construct did not affect TTL activity. hTTLL12 and TTL were efficiently expressed in these experiments (Figure 7B), showing that lack of activity was not due to absence of expression. We also tested the activity of TTL and hTTLL12 purified from mammalian BHK21 cells infected with appropriate Vaccinia virus Ankara strain (MVA) vectors [33]. As expected, TTL catalysed ligation of labelled tyrosine to tubulin in a dose dependent manner (Figure 7C). In contrast, equivalent amounts of hTTLL12 or hTTLL12 complexed to EF1A1 did not exhibit any activity relative to control reactions lacking purified proteins (the first bar). Adding hTTLL12 or hTTLL12 +EEF1A1 to TTL did not affect its activity, showing that these preparations did not contain contaminants that may have inhibited the reactions. Overall, these results suggest that hTTLL12 is devoid of TTL activity.

## Discussion

hTTLL12 is the most divergent member of the 14-gene TTL family in humans. It has a phylogenetically conserved association of two domains (SET- and TTL- like) that predict that it could be involved in histone and tubulin modification. We found that changes in hTTLL12 expression affect histone and tubulin modifications in cells. They also affect mitotic duration, which could be linked to both types of modification. However hTTLL12 appears to lack these histone and tubulin modification activities in vitro. These results suggest that hTTLL12 is an inactive enzyme homologue that may have a regulatory function.

TTLL12 is an unusual member of the TTL family, according to bioinformatics analysis. TTLL12 has a unique phylogenic distribution and it has features that distinguish it from the other family members in humans. For example, it is the only member of the TTLL family to be identified in plant genomes, such as Arapidopsis thaliana [34]. The TTL domain of hTTLL12 lacks three of the seven motifs that are conserved in the other family members. Furthermore, it has several residues that are specific to hTTLL12 and conserved orthologues in different species. The N-terminal region of hTTLL12 has homology to SET domains in a number of human proteins. The overall sequence similarity is low, which is a general characteristic of SET domains [for reviews see [5], [6], [7], [24]]. The TTLL12 SET domain has also been detected by Aravind et al [3], who studied the natural history of the eukaryotic chromatin protein modification system. They propose that the TTLL12 clade is one of at least 5 types of SET domains that were present in the LECA (last eukaryotic common ancestor). TTLL12 is one of the major lineages of SET domains that might have played the initial role in the establishment of multiple distinct heterochromatic and euchromatic states that are likely to have been present throughout much of eukaryotic evolution.

The presence of a SET-like domain in hTTLL12 prompted us to investigate its role in histone lysine methylation. We found that hTTLL12 is functionally linked to at least one histone methylation. Altering hTTLL12 levels affects H4K20me3 levels, without influencing other methylation states of H4K20 (me2 and me1) and methylation of H3K4 and H3K9. However, we did not detect histone methyl transferase activity in vitro with hTTLL12 proteins purified from various sources. hTTLL12 interacts with EEF1A1, raising the possibility that hTTLL12 may methylate this protein. EEF1A1 has been shown to be methylated and two histone methyltranferases that mediate this modification have been identified in Saccharomyces cerevisiae [35]. However, we did not detect methylation of EEF1A1 by hTTLL12 in our in-vitro methylation experiments. We propose that hTTLL12 affects H4K20 trimethylation in cells by an indirect mechanism, perhaps by altering the expression and/or interactions with factors implicated in this modification. hTTLL12's SET-like domain appears to lack a critical asparagine residue that is important for methyltransferase activity. A single asparagine to glutamine mutation in the SET domain of *Neurospora* DIM-5 strongly reduces the enzyme activity and binding to the cofactor SAM [S-Adenosyl-L-methionine, N241Q, [36]]. The predicted corresponding position in hTTLL12 (position 458; Alignment S2) is in fact a glutamine residue. hTTLL12 alters the expression levels of proteins that have indirect effects on trimethylation of H4K20 [Rb, HP1α; [26], [27], [37]]. TTLL12 has been shown to interact with SAP25 [38] and Arid1A [39], which are involved in nucleosome remodelling. It remains to be seen whether these interactions account for its effects on histone modification.

hTTLL12 is also functionally linked to tubulin tyrosination [this report and [2]]. This effect appears to be indirect, for a number of reasons. Increasing and decreasing hTTLL12 levels increase the levels of detyrosinated tubulin, which would be difficult to explain by a direct effect. In our experiments, hTTLL12 purified from a number of sources does not have tubulin tyrosine ligase activity. In other studies it was shown to lack polyglutamylation [23] and polyglycylation [40] activities. Lack of activity is also suggested by its sequence. In the alignment of TTL domains (Alignment S1) hTTLL12 seems to lack residues that are important for ATP/Mg^2+^ binding [GXGI→SLDT^426–429^ and FEψψGFD→RAMYAVD^578–584^ [41], [42], [43]]. The GXGI ATP/Mg^2+^ binding motif in hTTL is important for TTL activity, since this activity is abolished by mutation of the corresponding ^157^GEGI^160^ in the human TTL protein to ^157^GAAA^160^ (our unpublished data). Overall, our results suggest that hTTLL12 has indirect and perhaps regulatory roles in histone modification and tubulin detyrosination, rather than direct enzymatic activity.

Altered tubulin tyrosination and H4K20 methylation is compatible with the phenotypic effects of hTTLL12. hTTLL12 up- and down-regulation prolong mitotic duration. There are also more subtle defects in chromosome congression and positioning, in cells with the longest mitotic durations. Detyrosinated tubulin is enriched in the mitotic spindle, but less so in astral microtubules of the spindle [44]. Detyrosination could be an essential regulator of chromosome segregation, since the tyrosination state of α­tubulin regulates the activity of the depolymerizing kinesin MCAK [45], which is essential for proper chromosome segregation in anaphase [46]. H4K20 mono-methylation has been implicated in mitosis, but the link with tri-methylation is weaker [reviews: [47], [48], [49], [50]]. There are high levels of H4K20me3 in centromeric heterochromatin, which is required for the proper segregation of chromosomes during mitosis [51], [52]. hTTLL12 affects HP1α levels. HP1 proteins are required for correct chromosome segregation in Drosophila embryos [53], and during metaphase only HP1α, and not HP1β and HP1γ, co-localises with centromeric heterochromatin [54].

A role for hTTLL12 in mitosis is also supported by the MitoCheck consortium [55], [56]. They systematically analyzed genes and proteins that are required for chromosome segregation and cell division in human cells. 22,000 human genes were inactivated individually by RNA interference (RNAi) in cultured human cells. Cellular phenotypes were recorded by high-throughput live cell imaging. Automated analyses of images and movies revealed that about 600 out of the 22,000 genes play a role in mitosis. One of these genes is hTTLL12. The phenotypes observed were dynamic changes, segregation problems, strange nuclear shapes, nuclei that stay together and apoptosis. Different aspects of this phenotype were observed with 2 out of 4 dsRNAs used to down regulate hTTLL12 (http://www.mitocheck.org/cgi-bin/mtc?query=ttll12&query_type=genes). These phenotypes are compatible with our observations. Significantly, these phenotypes were not observed with any of the other members of the TTL family. hTTL and hTTLL1 were found to have effects on secretion, and hTTLL4 on apoptosis.

We found that hTTLL12 induces changes in DNA content and chromosome numbers by FACS and karyotype analysis, respectively. The changes in chromosome number are observed in some cells in each clone, which are composed of a mixed population of cells with different chromosome numbers. This suggests that hTTLL12 overexpression favours relatively rare events that are not completely eliminated by cell death. A strong effect on chromosome number would be expected to be lethal to the cell. hTTLL12 appears to predispose cells to low frequency events that may accumulate with time, and perhaps help cells adapt during tumour progression. hTTLL12 has been linked to altered chromosome numbers by others. In a study of amniotic fluid from fetuses with the two most common human autosomal aneuploidies, trisomies 21 and 18, in which RNA expression was compared to euploid fetuses matched for sex and gestational age, one of the four RNAs that was upregulated in both comparisons was hTTLL12. hTTLL12 does not map to the chromosomes with altered copy number, suggesting that copy number did not account for the differences [57], [58]. It remains to be seen whether there is any connection between these studies and our observations.

Our working hypothesis is that hTTLL12 could be a pseudo-enzyme that regulates histone and tubulin modifications and thereby microtubule dynamics, mitosis and chromosome number instability. hTTLL12 may exploit its ability to bind the same substrates and cofactors as other TTLs and SET domain factors. Consequently, hTTLL12 would be well placed to directly or indirectly regulate the biological processes in which its cognate enzymes act. Such enzymes could include the many tubulin-modifying and -demodifying enzymes that have been recently characterised and shown to regulate microtubule functions through different modifications. Combinatorial use of tubulin modifications could generate a dynamic microtubule code [10], [12]. hTTLL12 would thus resemble the numerous other inactive enzyme homologues that contain conserved substitutions in their catalytic sites and have regulatory roles [59]. Most enzyme families have inactive homologues (pseudo-enzymes) that are well conserved. They appear to have sufficient functional importance to resist selective pressure during evolution. hTTLL12 may be another example of a dead enzyme with an important physiological role. It is possible that the study of hTTLL12 could offer novel insights into biochemical pathways controlled by its active counterparts.

## Supporting Information

Supporting Experimental Procedures S1. (PDF)

Figure S1 **KIAA0153 might be part of a complex containing EF1A1, α- and β-tubulin.** *A* Purification of flag-hTTLL12 by affinity and further Coomassie staining revealed an extra band identified as EF1A1 by Mass Spectrometry. 1: total cell extract, 2: column flow through, 3: column wash, 4: proteins eluted by peptide competition. *B* Silver stained gels and bands identified by Mass Spectrometry. 1: marker, 2 and 3: proteins eluted from columns loaded with cell extract from vector stable transfectant (2), and sense stable transfectant. Major proteins identified are indicated. The other bands correspond to heat shock proteins and degradation products from either hTTLL12 or EF1A1 (not indicated). 75, 50, 37 correspond to molecular weight markers (×10^−3^ kD).
(PDF)

Figure S2 **hTTLL12 localises to mitotic spindle microtubules and centrosomes.** HEp-2 cells were fixed and co-stained with hTTLL12 (2065, red) and β-tubulin (green) (panel A), hTTLL12 (2089, red) and β-tubulin (green) (panel B) or hTTLL12 (2089, red) and γ-tubulin (green) (panel C) specific antibodies. Images were taken by confocal microscopy.
(PDF)

Figure S3 **hTTLL12 expression deregulation in HEp-2 cells alters growth and DNA-profiles.** *A.* Growth of Control (Control_A-E) and hTTLL12 (hTTLL12_A-E) clones was measured by MTT in quadruplicate during ten days. Data represent average values ± average deviation of Control (black) and hTTLL12 (white) clones. * Statistically significant difference to Control cells (*P*<0.05, Student's *t*-test). *B.* hTTLL12 (hTTLL12_A-E) or Control (Control_A, _B, _D, _E) clones were seeded, harvested in Laemmli buffer and subjected to SDS-PAGE and WB (20 µg protein/lane). Histone H4 (H4), Rb, HP1α, HP1β and HP1gamma levels were quantified by densitometry and normalized to TBP. Data represent average protein levels ± SEM in hTTLL12 lysates relative to average levels in Control lysates (n = 3 for Rb, HP1β, HP1gamma, n = 4 for H4, HP1α). *C.* The DNA profile of controls (HEp-2, Control_A, _B, _F, and _G; purple bars) and hTTLL12 clones (hTTLL12_A-E; red bars) was analyzed by FACs after propidium iodide staining. 2c, 4c and 8c: diploid, tetraploid and octaploid DNA contents. D. Chromosome numbers per cell. For each clone (Control_A-E: “triangle” A, “dot” B, “cross” C, “square” D, “diamond” E, green; hTTLL12_A-E: “dot” A, “cross” B, “square” C, “diamond” D, “triangle” E, red), sixty-five metaphase spreads (1–65, y axis) were stained with Giemsa and the number of chromosomes per mitosis was counted (X-axis). The modal chromosome number of HEp-2 cells is 74. * Statistically significant difference between the average chromosome numbers in hTTLL12 and Control clones (*P*<0.05, Student's *t*-test).
(PDF)

Figure S4 **Altering hTTLL12 expression prolongs mitotic duration (see** **Fig. 5** **).** Mitotic duration was scored from time-lapse movies (Experimental Procedures). The total numbers of clone or transfected cells analysed were: 205 HEp-2, 263 Control_A-E, 372 hTTLL12_A-E, 591 control siRNAs (siCtrl, siLuciferase, siScramble, used separately and combined), and 899 sihTTLL12_1-6, used separately). *A.* Data represent average mitotic duration ± STDEV (standard deviation) for HEp-2, hTTLL12 and Control clones, and control siRNAs (siControl) and sihTTLL12 transfected cells. * Statistically significant difference between hTTLL12 clones and Control clones or Hep-2, as well as between sihTTLL12 and siControl or HEp-2 (*P*<0.05, Student's *t*-test). *B.* Average percentages for each clone type (HEp-2, Control_A-E and hTTLL12_A-E) ± STDEV divided into time intervals. * Statistically significant difference between hTTLL12 clones and Control clones or HEp-2 (*P*<0.05, Student's *t*-test). *C.* Average percentages for each siRNA type (non-transfected (HEp-2), siControl (siCtrl, siLuciferase, siScramble) and siTTLL12 (sihTTLL12_1-6)) ± STDEV divided into time intervals. * Statistically significant difference between sihTTLL12 transfected cells and siControl or HEp-2 (*P*<0.05, Student's *t*-test).
(PDF)

Figure S5 **hTTLL12 overexpression leads to chromosome congression delay in HEp-2 cells.** The total numbers of cells analysed by time lapse movies (Experimental Procedures) were 420 for Control_A-E and 491 for hTTLL12_A, _B, _D, and _E. *A.* Representative fluorescent (Hoechst) images of metaphases of a HEp-2 cell (Control), and a hTTLL12 overexpressing cell showing congression delay. Red arrows mark chromosomes that are delayed. 0 min is the time of NEB. *B.* Average percentages for each clone type ± STDEV having congression delay divided in time intervals (mitotic duration ≤50 min or >50 min) scored from time lapse movies of hTTLL12 and Control clones. * Statistically significant difference between Control and hTTLL12 clones (*P*<0.05, Student's *t*-test).
(PDF)

Figure S6 **hTTLL12 up and down regulation leads to a spindle positioning phenotype in HEp-2 cells.** The orientation and placement of mitotic spindles was scored from time-lapse movies (Experimental Procedures). The numbers of cells analysed were: 262 HEp-2, 420 Control_A-E, 380 hTTLL12_A, _B, _D clones, 502 control siRNAs (siCtrl, siLuciferase, siScramble, used separately and combined), and 973 sihTTLL12 (sihTTLL12_1-6, used separately). *A.* Phase contrast and fluorescent (Hoechst) image overlays of metaphases from representative cells: a HEp-2 (Control, upper panel), a hTTLL12 clone (hTTLL12, middle panel) and a sihTTLL12 transfected cell (sihTTLL12, lower panel). Red lines and grey circles highlight metaphase plate position with respect to the circumference of the cell. 0 min is the time of anaphase onset. *B.* Average percentages for each cell type ± STDEV with the spindle positioning phenotype divided into time intervals (mitotic duration ≤50 min or >50 min) for HEp-2 cells, hTTLL12 and Control clones. * Statistically significant (*P*<0.05, Student's *t*-test) difference between hTTLL12 clones and Control clones or HEp-2 cells. *C.* Average percentage for each clone or cell type ± STDEV with the spindle positioning phenotype divided into time intervals (mitotic duration ≤50 min or >50 min) scored from time lapse movies with HEp-2 or cell transfected with control siRNAs or sihTTLL12s. * Statistically significant difference between sihTTLL12 and control siRNA transfected cells or HEp-2 (*P*<0.05, Student's *t*-test).
(PDF)

Table S1 **Amino acid conservation in the TTL core domain of TTL family members.** The consensus sequence for the human family and the corresponding sequence in hTTLL12 are from Alignment S1. The alignment for different species is not shown.
(PDF)

Table S2 **The sequences and their positions in hTTLL12 that are highly conserved in other human SET domains (see Alignment S2) are listed.** Letters are coloured according to Fig. 1 in Qian et al. (38): red: absolutely conserved; blue: very highly conserved; black: highly conserved). The corresponding conserved structural elements are indicated (38). Amino acids 158–188 are the approximate limit of a predicted insert in the SET domain that is deduced from Alignment S2. Sequence alignments of hTTLL12 with several plant and unicellular ciliate protozoan orthologues (e.g. NP_177879.3, *Arabidopsis thaliana*; XP_002326123 *Populus trichocarpa*; ABF94289, *Oryza sativa*; XP_001020206, *Tetrahymena thermophila* SB210; XP_001454840, *Paramecium tetraurelia* strain d4-2; XP_001580810, *Trichomonas vaginalis* G3) indicate that these orthologues have an insert that has an additional sequence inserted in this position (data not shown).
(PDF)

Table S3 **The 55 kDa co-immunoprecipitated protein band (** **Fig. 3C** **) corresponds to α-tubulin.** The table summarizes tryptic peptide sequences, their relative positions on the α-tubulin primary protein sequence, their measured masses and computed masses. * oxidized methionine.
(PDF)

Alignment S1 **Multiple sequence alignment of the TTL domains of the human TTL protein family members.**
(PDF)

Alignment S2 **Multiple sequence alignment of the hTTLL12 SET-like domain and the SET domains of human proteins.**
(PDF)
