# Supplementary material for: Tubulin Tyrosine Ligase Like 12, a TTLL Family Member with SET- and TTL-Like Domains and Roles in Histone and Tubulin Modifications and Mitosis
Source: PLoS One. 2012 Dec 12;7(12):e51258. doi: 10.1371/journal.pone.0051258 (PMC3520985; doi:10.1371/journal.pone.0051258)
Supplement: Supporting Experimental Procedures S1. — (PDF) [file pone.0051258.s001.pdf]

Supporting Experimental Procedures

Plasmid constructions- Human *TTLL12* was cloned from the 22Rv1 cell line (manuscript in preparation). For the expression of N-terminal Flag epitope-tagged proteins, the coding region was PCR amplified (primer set: 5'-CGCCTCGAGATGGAGGCCGAGCGGGGT-3' / 5'-CGCTGATCACTAGACAAGGCAGGTAACG-3') and cloned in the XhoI/BglII restriction sites of the pSG5-puro-Flag expression vector (31). A similar antisense expression plasmid was created by PCR amplification of the coding region (primer set: 5'-CGCCTCGAGATGGAGGCCGAGCGGGGT-3' / CGCCTCGAGCTAGACAAGGCAGGTAACG-3') and cloning in reverse direction in the XhoI restriction site of psg5-puro-Flag vector (ash*TTLL12*). All plasmid constructs were verified by sequence analysis.

siRNAs- siRNAs used in this study include: non-targeting control siRNAs: siCtrl (CONTROL® Non-Targeting siRNA #1, Dharmacon); siLuciferase (GL2 *luciferase* siRNA, Dharmacon); siSilencer (Silencer® Negative Control #1 siRNA, Ambion); siGFP (GFP-22 siRNA, Qiagen); siScramble (siCLIP170 scramble 5'-GGUACUUAGGAGAACCAAA-3', Invitrogen), h*TTLL12*-specific siRNAs: sihTTLL12_1: 5'-GAGUUCAUCCCCGAGUUUG-3'; sihTTLL12_2: 5'-GGAACGAGCUGUGCUACAA-3'; sihTTLL12_3: 5'-AAGGCCAUCUUCUCUUAAA-3'; siTTLL12_4: 5'-ACGCCGACAUCCUCUUCAA-3'; sihTTLL12_5: 5'-GUAGCGGUGUCUCCUCUUU-3' (Dharmacon); sihTTLL12_6: 5'-GGUUGUUCGUGUAUGAUGU-3' (Sigma-Proligo) and *TTL*-specific siRNAs: siTTL_1: 5'-GAAAGGCAACUCGCAAAGA-3'; siTTL_2: 5'- UAACUCAAGGACAGAUGAA-3' (Dharmacon).

Antisera and antibodies- hTTLL12-specific sera 2089 and 2562 were raised in rabbits immunized with peptides NH_2-EDEAAREVRKQQPNPGNE-COOH and NH_2-EDEAAREVRKQQPNPG-COOH, respectively. Serum 2565 was purified on a peptide column. Primary antibodies used in WB: anti hTTLL12 2089 (1/3000), anti detyrosinated tubulin antibody (1/1000, AbCys) and anti α-tubulin antibody (1/2500, clone DM1A, Sigma); anti Histone H3 and -H4 (1/200, 1/100, Santa Cruz); anti TATA binding protein, (TBP, clone 3TF1/3G3, 1/2000), anti HP1α, -β and –γ (1/2000) were obtained from the IGBMC antibody facility; all other antibodies were purchased from Abcam: anti H4K20me1, -3 (1/1000), anti H4K20me2 (1/2000), anti H3K9me2 (1/2000) H3K9me3 (1/500), anti H3K4me2 (1/3000), anti H3K4me3 (1/200), anti H3K27me2 (1/600), anti HP1γ (1/2000), and anti Rb (1/200). Peroxidase-labelled secondary antibodies used in WB: goat-anti mouse and goat-anti rabbit (1/10000; Santa Cruz Biotechnology).

Primary antibodies used in immunocytochemistry: anti hTTLL12 2089 and 2562 (1/400), anti β-tubulin (1/600, clone H-235, Santa Cruz) and anti γ–tubulin (1/600, clone C11, Santa Cruz). Secondary antibodies used**:** Alexa Fluor 488 and 594 conjugated secondary antibodies (1/2000, Molecular Probes, Inc.)

Multiple sequence alignments- Proteins included in the SET domain alignment: hTTLL12, SETD2, SETD3, SETD4, SETD5, SETD6, SETD7, SETD8, PRDM1, PRDM2, PRDM5, PRDM6, SUV41, SUV42, EZH1, EZH2, SETB1, SETB2, SETMR, EHMT1, EHMT2, SUV91, SUV92, ASH1L, NSD1, NSD2, NSD3, HRX, WBP7, SET1A, MLL2, MLL3, MLL5 and an un-annotated set-domain containing protein (accession number http://www.expasy.org/sprot/userman.html - DE_lineQ6ZW69). Proteins included in the TTL domain aligment: TTL, TTLL1, TTLL2, TTLL3, TTLL4, TTLL5, TTLL6, TTLL7, TTLL8, TTLL9, TTLL10, TTLL11, hTTLL12 and TTLL13 (accession numbers Q8NG68, O95922, Q9BWV7, Q9Y4R7, Q14679, Q6EMB2, Q8IYW6, Q6ZT98, XP_104657, Q3SXZ7, Q6ZVT0, Q8NHH1, Q14166 and NP_001025135, respectively).

Mass spectrometry- Bands were excised from the silver stained gel and digested as previously described (62) with 5–10 µl (depending on the gel volume) of 10 ng/µl of freshly diluted trypsin in 25 mM NH_4HCO_3 overnight at 30 °C. Five microliters of 40% H_2O/60% acetonitrile/0.1% TFA was added and

after 4 hours at room temperature, the mixture was sonicated for 2 minutes and centrifuged. 0.5 µl of the supernatant was mixed with an equal volume of saturated DHB (Sigma) dissolved in 20% acetonitrile and applied to the target. MALDI mass measurements were carried out on a Bruker Reflex IV MALDI-TOF spectrometer, using a maximum accelerating potential of 20 kV in the positive reflector mode. The acquisition mass range was 800–3000 Da with the low mass gate set at 700 Da. Internal calibration was performed using autolytic trypsin peptides (MH+: m/z = 842.51 and m/z = 2211.11). Monoisotopic peptide masses were assigned using the Bruker Flex Analysis software. The Profound program was used for database searching (http://prowl.rockefeller.edu/) with the following parameters: database NCBI, taxonomy mammalian, protein mass range 0–150 kDa, trypsin digestion with one missed cleavage allowed, cysteines modified by carbamidomethylation, methionine oxidation, and mass tolerance of 75 ppM.

SDS-PAGE and WB- Cells were washed twice with cold PBS, lysed in Laemmli buffer (62.5 mM Tris-HCl, pH 6.8, 10% glycerol, 2% SDS) supplemented with 1 mM NaF, 1 mM $NaVO_3$, 1 mM PMSF and Complete® protease inhibitors (Roche), sonicated and cleared before protein quantification (D_C BioRad Protein Assay). Samples with equal protein quantity were supplemented with 5% β-mercaptoethanol, heated (95 °C; 12 minutes); size fractionated on a 9% or 12% SDS-PAGE gel and transferred to nitrocullulose membranes. All Blue Precision Plus Protein Standard (Bio-Rad) was used as protein size marker. Membranes were blocked for 45 minutes in PBS-T-milk (0.05% Tween, 5% dried fat-free milk); incubated with primary antibody (3 hours; RT; diluted in PBS-T-milk), washed (PBS-T) and incubated with secondary antibodies (1 hour; RT; diluted in PBS-T-milk). Primary and secondary antibodies used are described above. For TBP detection, no milk was used for antibody incubations and no Tween was used during blocking and primary antibody incubations. After removal of unbound secondary antibodies, signals were revealed using SuperSignal West Pico Chemiluminiscent Substrate (Pierce) and detected using a VersaDoc™ image station (Bio-Rad) or film (KODAK BioMax MR Film). When using image station, TBP and detyrosinated tubulin signals were visualized using SuperSignal West Femto Maximum Sensitivity Substrate (Pierce). Signals were quantified using Quantity One software or densitometry when detected by image station or film, respectively. For synchronised cells protein extracts, the cells were washed twice with cold PBS and lysed immediately with protein loading buffer (125mM Tris base, 141mM SDS, 185mM HCl, 4.3mM glycerol, 0.2mM NaF, 0.1mM NaVO4, 0.5mM PMSF, 10mM DTT, 0.625mM β-mercaptol). Protein quantity was considered to be equal when TBP bands had similar signal intensity. PageRuler™ Plus prestained protein ladder (Fermentas) was used as protein size marker. For TBP and HP1α membranes were blocked in PBS-T-milk (0.05% Tween, 10% dried fat-free milk); for H4K20me3 and detyrosinated tubulin – in TBS-T-BSA (0.05% Tween, 3% dried BSA). Primary antibodies were diluted in corresponding solutions but in 1% milk or 1% BSA accordingly, secondary in 0.5% milk or 0.1% BSA. Primary antibody was applied overnight at +4°C, secondary for 1h at room temperature; in between and after antibody applications the membranes were washed in PBS-T or TBS-T accordingly. Chemiluminiscent signals were detected with ImageQuant LAS 4000 (GE); bands were quantified with ImageQuant TL 7.0 software (GE).

Immunocytochemistry and Confocal Microscopy- HEp-2 cells were plated onto sterilized glass cover slips and after 18 hours (80% confluence) they were incubated for 6 hours in low serum followed by 2 hours in 10% foetal calf serum in the culture medium. Cells were washed (PBS) and fixed using the Cytoskelfix™ kit (Cytoskeleton). Antibody labelling and mounting of the slides was done as described in (33). Details for primary and secondary antibodies are described above. Images were collected using a SP2A confocal microscope (object x100, zoom 5.85; Field 50x50 µm). For each field, 20 to 40 z-sections of 0.137 µm were analyzed and compiled using TIMT software (IGBMC). The images represent the compilation of z-sections corresponding to the centre of the cell.

Tubulin tyrosine ligase (TTL) assay- The reactions contained either transfected cell extracts or purified proteins. To prepare cell extracts, transfected cells were harvested by a brief trypsin treatment, transferred to tubes containing PBS-10% fetal calf serum and counted. The cells were collected by centrifugation at 950 rpm for 5 min at RT, washed twice with PBS, centrifuged and resuspended in TTL buffer (50 mM

Tris-HCl pH 7.4, 12.5 mM $MgCl_2$, 100 mM KCl, 1 mM DTT, 5% glycerol, 50 μg/ml RNase A), subjected to 3 cycles of freezing-thawing (liquid N_2-37°C) and centrifuged at 10000 rpm for 10 min at 4°C. We assayed in triplicate and, typically, the amount of cell extract corresponded to $3x10^5$ cells. In some experiments, cells were washed, scraped directly in TTL buffer and lysed as indicated above. Identical amount of cell extracts were assayed. The assay mixture contains 4 mM ATP, 200 μg/ml purified rabbit tubulin, 0.5 μCi of L-[U-^{14}C]Tyrosine (434 mCi/mmol; Amersham) in addition to the TTL buffer (final volume 50 μl). Rabbit tubulin was purified by two cycles of *in vitro* polymerization (Shelanski et al. 1973). The samples were incubated for 1 hour at 37°C, stopped on ice and 50 μl aliquots immediately pipetted into 1 ml of 10% trichloroacetic acid (TCA) containing 20 μg of unlabelled tyrosine (Sigma). BSA (200 μg) was added as a carrier. The TCA precipitates were kept at 0°C for 30 min and collected on GFC Whatman glass fibre filters. The filters were washed three times with 5 ml of 10% TCA, dried, placed in scintillation liquid cocktail (ReadySafe™, Beckman) and counted in a Beckman scintillation counter.

In vitro histone methyltransferase assays, materials- Histones calf thymus 10223565001 Roche Lot 70212520 Nov 2009. Recombinants: pGEX-2T GST-NSD1 (1700-1987) (1) (gift from R Losson). pGEX GST-hTTLL12 (50-250) (details of construction can be obtained from C Birck). Vaccinia virus Ankara strain (MVA) vectors for TTL (2) and hTTLL12 (details can be obtained from R Drillien). Clones hTTLL12_C, hTTLL12_D, Control_D Methods: Expression and purification of proteins

In vitro histone methyltransferase assays, E. coli expression- Recombinant GST fusion proteins were expressed using the pGEX system in Escherichia coli BL21 and purified on glutathione-Sepharose beads (Pharmacia) using standard procedures. The yield and purity were verified on Coomassie Blue G250 stained 10% SDS-PAGE, using BSA as a standard. The GST fusion proteins were of the expected size and were the only major bands on the stained gels.

In vitro histone methyltransferase assays, Vaccinia virus expression- TTL and hTTLL12 were expressed using vaccinia virus Ankara strain (MVA) in BHK21 cells as described previously (2) and purified on his tag affinity columns (3) followed by gel filtration. Two hTTLL12 preparations were tested, hTTLL12 alone, or in complex with EEF1A1 in an approximate 1:1 ratio (detailed procedures are available from R. Drillien & C. Birck). The proteins were of the expected size and were the only major bands on the stained gels.

In vitro histone methyltransferase assays, mammalian cell expression, using hTTLL12 HEp-2 clones- Cells were initially fractionated into cytoplasmic, nuclear wash and high salt nuclear fractions, essentially as described by Dignam et al (4). About 10^7 cells (2x10 cm plates, 80% confluent) of HEp-2 hTTLL12 or control clones were washed 3 times with PBS + protease inhibitors, scrapped, centrifuged 2 min at 10^3 rpm, resuspended in 1.5 ml buffer A, incubated on ice for 15 min, adjusted to 0.5% NP40, incubated 20 min, centrifuged at 10^4 rpm for 5 min at 4°C. The supernatants were adjusted to 150 mM KCl and considered to be the cytoplasmic fractions. The pellets were washed by twice resuspending in 1 ml PBS and centrifuging 2 min at 2 x 10^3 rpm. Nuclear extracts were made by resuspending the pellets in buffer C, incubating 40 min with vortexing at 10 min intervals, and centrifugation at 12 x 10^3 rpm for 20 min. The supernatant was the nuclear fraction. The pellet was resuspended in 0.3 ml of buffer C-HS (0.4 ml solution C and 1.6 ml of 2 M KCl; to give a final concentration of approximately 1 M KCl), vortexed, sheared by centrifugation through a QIAshredder (Qiagen), incubated 40 min, and centrifuged for 10 min at 10^4 K rpm, washed with 0.15 ml buffer C-HS. The combined initial and wash supernatants were combined to give the 1M fraction. The cytoplasmic, nuclear and 1M fractions were purified with 100 μl of mouse anti-flag® M2 agarose IGgγ1 clone M2 (Sigma-Aldrich Chimie SARL) beads essentially according to the manufacturer's instructions. Briefly, the extracts were incubated with pH3.8 pre-treated beads by rotation overnight at 4°C, washed 4 times with 1.6 ml IP M2 buffer, 2 times with 800 μl TBS containing protease inhibitors and resuspended in 100 μl MAB buffer. The amount of absorbed proteins was estimated on Coomassie Blue stained 10% SDS-PAGE gels in comparison with known quantities of BSA. hTTLL12 Hep-2 clone extracts gave two major bands that were absent in the control clone extracts. They were shown to correspond to hTTLL12 and EEF1A1 by Western blotting and size. The bead-bound

proteins were used for the HMTase assays.

In vitro histone methyltransferase assays, incubations-HMTase assays were performed essentially as described by Rayasam et al., 2003 (1). 20 µl reactions containing substrates [20 µg of native calf thymus histones (Roche Diagnostics)], putative enzymes (1-2 µg of GST-bound proteins, 2-4 µg hTTLL12, 2 µg hTTLL12 + EEF1A1, and 4 µg TTL) and 250 nCi of S-adenosyl- L-[methyl-^{3}H]- methionine (25 mCi/ml) (GE Healthcare Europe) in methyltransferase activity buffer [MAB: 50 mM Tris-HCl pH 9.0, 1 mM phenylmethylsulfonyl fuoride (PMSF), 0.5 mM β-mercaptoethanol] were incubated for 30 min at 30°C. Reactions were stopped by boiling in SDS loading buffer, and reaction products were separated on 15% SDS-PAGE and visualized by PageBlue (Fermentas GMBH) staining and fuorography using Amplify fluorographic reagent (GE Healthcare Europe).

Flag-hTTLL12 pull-down and mass spectrometry- M2 Flagged-hTTLL12 was purified from hTTLL12_E cell line by immunoaffinity with anti-Flag M2 resin (Sigma) following the manufacturer's instructions. As negative control, we performed, in parallel, affinity purification on cell extracts from a Control clone. Proteins were eluted by competition with M2 Flag peptide and fractionated by 8% SDS–PAGE. The gels were silver stained using the ProteoSilver Plus silver stain kit (Sigma-Aldrich) according to the manufacturer's instructions.

Karyotype analysis- Metaphase spreads and Giemsa staining of the parental HEp-2 cell line, five hTTLL12 and five Control clones were made according to standard procedures (21). Chromosomes were counted using SkyView software (Applied Spectral Imaging).
