# Supplementary figures and images for: Tubulin Tyrosine Ligase Like 12, a TTLL Family Member with SET- and TTL-Like Domains and Roles in Histone and Tubulin Modifications and Mitosis
Source: PLoS One. 2012 Dec 12;7(12):e51258. doi: 10.1371/journal.pone.0051258 (PMC3520985; doi:10.1371/journal.pone.0051258)

Figure S1

Purification of flag-hTTLL12 from stable transfectant

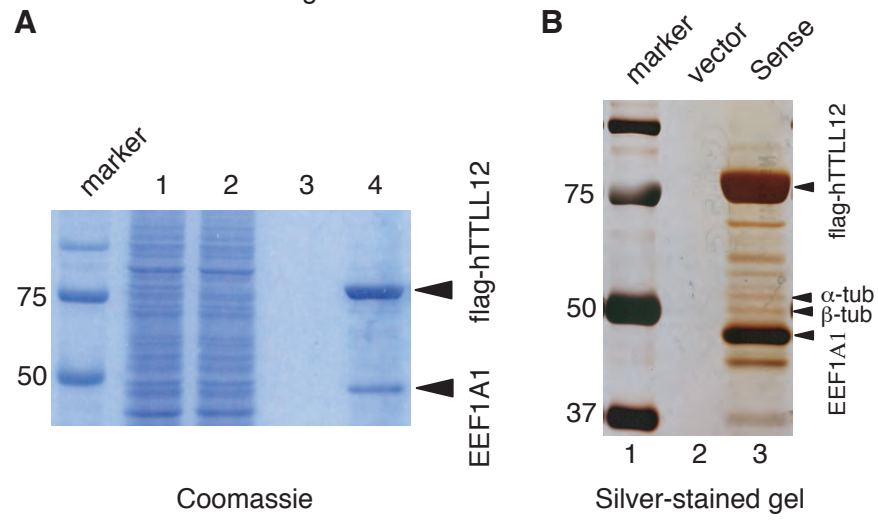

Supplement: Figure S1 — KIAA0153 might be part of a complex containing EF1A1, α- and β-tubulin. A Purification of flag-hTTLL12 by affinity and further Coomassie staining revealed an extra band identified as EF1A1 by Mass Spectrometry. 1: total cell extract, 2: column flow through, 3: column wash, 4: proteins eluted by peptide competition. B Silver stained gels and bands identified by Mass Spectrometry. 1: marker, 2 and 3: proteins eluted from columns loaded with cell extract from vector stable transfectant (2), and sense stable transfectant. Major proteins identified are indicated. The other bands correspond to heat shock proteins and degradation products from either hTTLL12 or EF1A1 (not indicated). 75, 50, 37 correspond to molecular weight markers (×10−3 kD). (PDF) [file pone.0051258.s002.pdf]

**Figure S2**

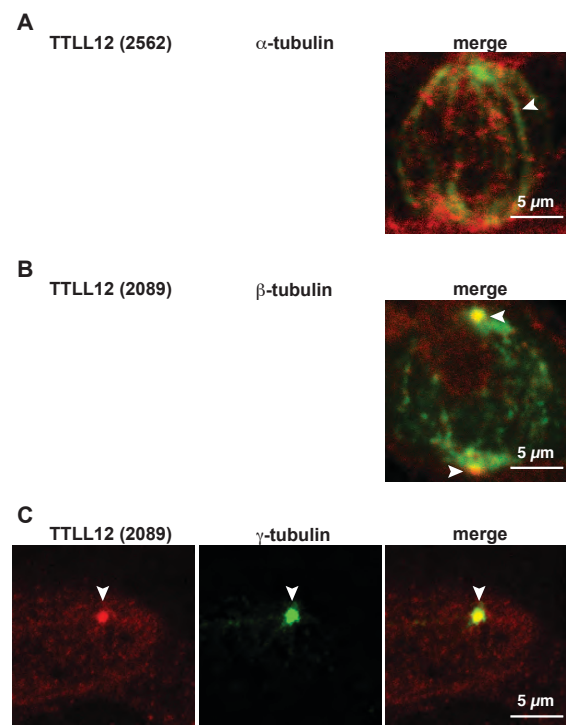

Supplement: Figure S2 — hTTLL12 localises to mitotic spindle microtubules and centrosomes. HEp-2 cells were fixed and co-stained with hTTLL12 (2065, red) and β-tubulin (green) (panel A), hTTLL12 (2089, red) and β-tubulin (green) (panel B) or hTTLL12 (2089, red) and γ-tubulin (green) (panel C) specific antibodies. Images were taken by confocal microscopy. (PDF) [file pone.0051258.s003.pdf]

Figure S3

A

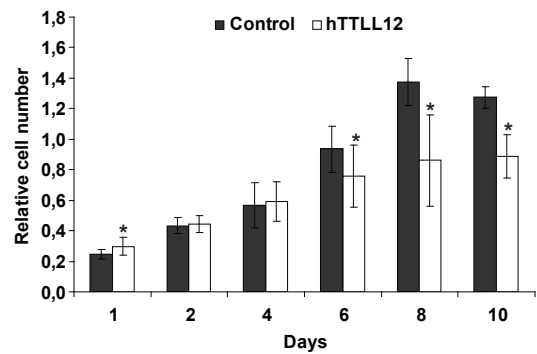

B

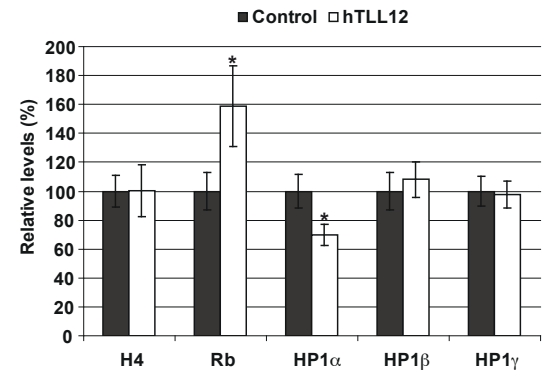

C

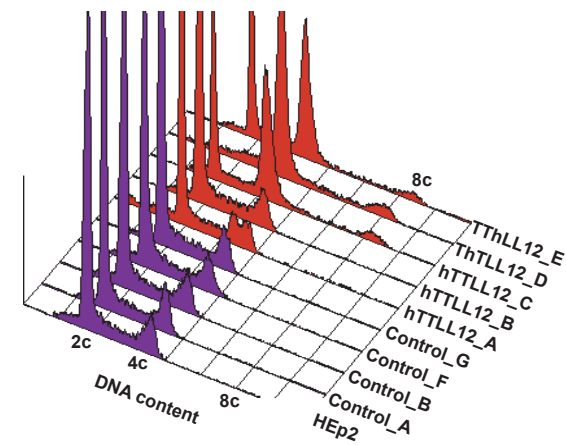

D

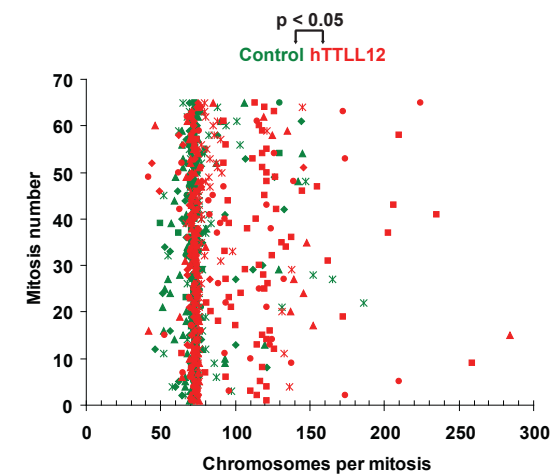

Supplement: Figure S3 — hTTLL12 expression deregulation in HEp-2 cells alters growth and DNA-profiles. A. Growth of Control (Control_A-E) and hTTLL12 (hTTLL12_A-E) clones was measured by MTT in quadruplicate during ten days. Data represent average values ± average deviation of Control (black) and hTTLL12 (white) clones. * Statistically significant difference to Control cells (P<0.05, Student's t-test). B. hTTLL12 (hTTLL12_A-E) or Control (Control_A, _B, _D, _E) clones were seeded, harvested in Laemmli buffer and subjected to SDS-PAGE and WB (20 µg protein/lane). Histone H4 (H4), Rb, HP1α, HP1β and HP1gamma levels were quantified by densitometry and normalized to TBP. Data represent average protein levels ± SEM in hTTLL12 lysates relative to average levels in Control lysates (n = 3 for Rb, HP1β, HP1gamma, n = 4 for H4, HP1α). C. The DNA profile of controls (HEp-2, Control_A, _B, _F, and _G; purple bars) and hTTLL12 clones (hTTLL12_A-E; red bars) was analyzed by FACs after propidium iodide staining. 2c, 4c and 8c: diploid, tetraploid and octaploid DNA contents. D. Chromosome numbers per cell. For each clone (Control_A-E: “triangle” A, “dot” B, “cross” C, “square” D, “diamond” E, green; hTTLL12_A-E: “dot” A, “cross” B, “square” C, “diamond” D, “triangle” E, red), sixty-five metaphase spreads (1–65, y axis) were stained with Giemsa and the number of chromosomes per mitosis was counted (X-axis). The modal chromosome number of HEp-2 cells is 74. * Statistically significant difference between the average chromosome numbers in hTTLL12 and Control clones (P<0.05, Student's t-test). (PDF) [file pone.0051258.s004.pdf]

Figure S4

A

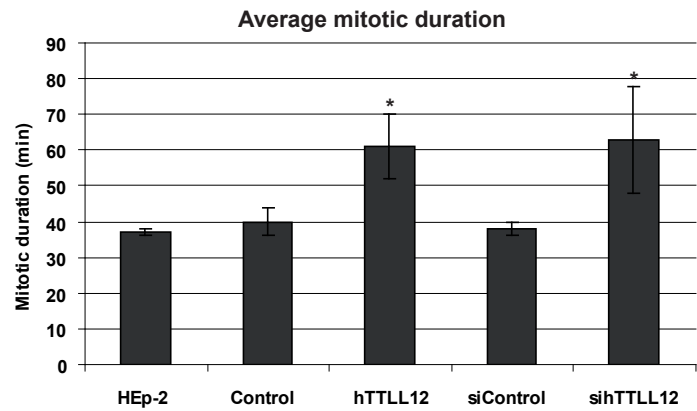

B

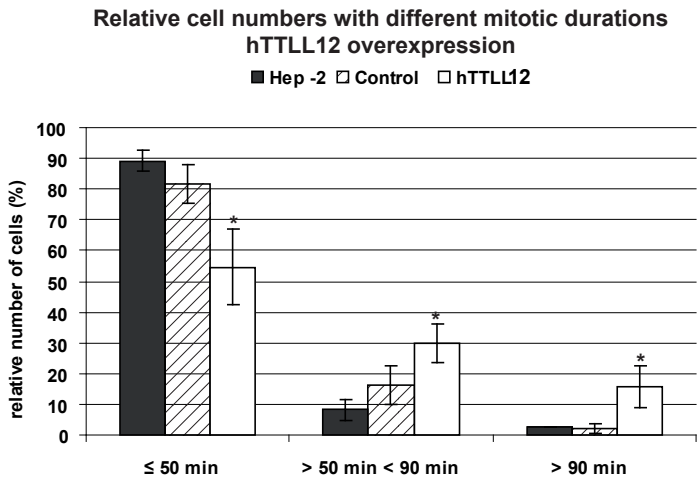

C

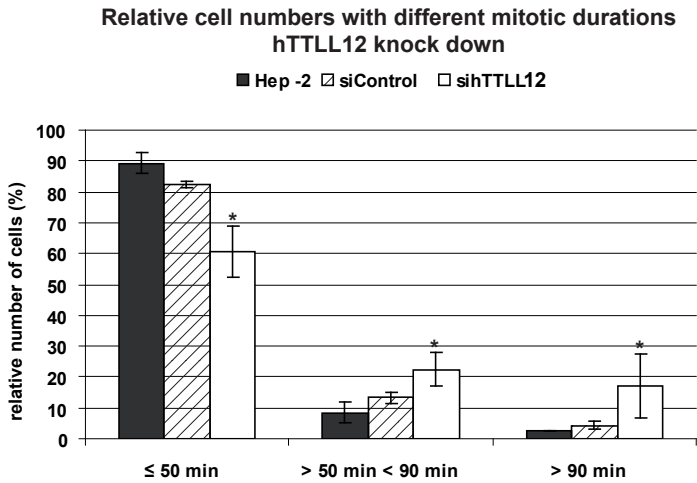

Supplement: Figure S4 — Altering hTTLL12 expression prolongs mitotic duration (see Fig. 5 ). Mitotic duration was scored from time-lapse movies (Experimental Procedures). The total numbers of clone or transfected cells analysed were: 205 HEp-2, 263 Control_A-E, 372 hTTLL12_A-E, 591 control siRNAs (siCtrl, siLuciferase, siScramble, used separately and combined), and 899 sihTTLL12_1-6, used separately). A. Data represent average mitotic duration ± STDEV (standard deviation) for HEp-2, hTTLL12 and Control clones, and control siRNAs (siControl) and sihTTLL12 transfected cells. * Statistically significant difference between hTTLL12 clones and Control clones or Hep-2, as well as between sihTTLL12 and siControl or HEp-2 (P<0.05, Student's t-test). B. Average percentages for each clone type (HEp-2, Control_A-E and hTTLL12_A-E) ± STDEV divided into time intervals. * Statistically significant difference between hTTLL12 clones and Control clones or HEp-2 (P<0.05, Student's t-test). C. Average percentages for each siRNA type (non-transfected (HEp-2), siControl (siCtrl, siLuciferase, siScramble) and siTTLL12 (sihTTLL12_1-6)) ± STDEV divided into time intervals. * Statistically significant difference between sihTTLL12 transfected cells and siControl or HEp-2 (P<0.05, Student's t-test). (PDF) [file pone.0051258.s005.pdf]

Figure S5

A

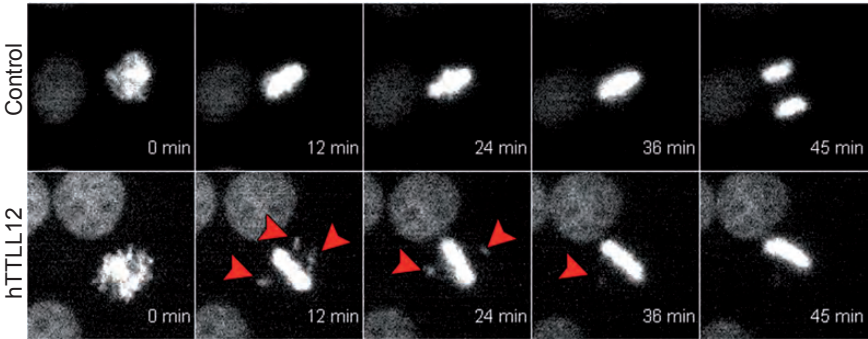

B

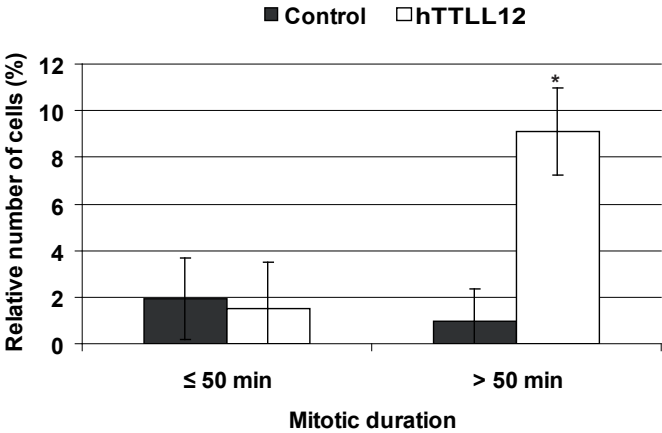

Supplement: Figure S5 — hTTLL12 overexpression leads to chromosome congression delay in HEp-2 cells. The total numbers of cells analysed by time lapse movies (Experimental Procedures) were 420 for Control_A-E and 491 for hTTLL12_A, _B, _D, and _E. A. Representative fluorescent (Hoechst) images of metaphases of a HEp-2 cell (Control), and a hTTLL12 overexpressing cell showing congression delay. Red arrows mark chromosomes that are delayed. 0 min is the time of NEB. B. Average percentages for each clone type ± STDEV having congression delay divided in time intervals (mitotic duration ≤50 min or >50 min) scored from time lapse movies of hTTLL12 and Control clones. * Statistically significant difference between Control and hTTLL12 clones (P<0.05, Student's t-test). (PDF) [file pone.0051258.s006.pdf]

Figure S6

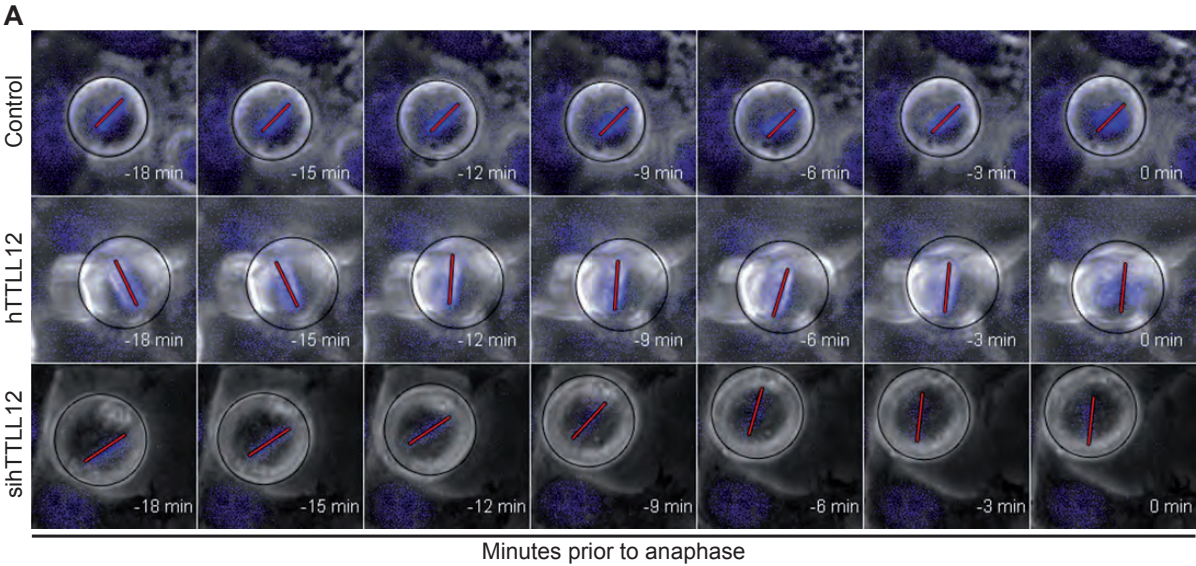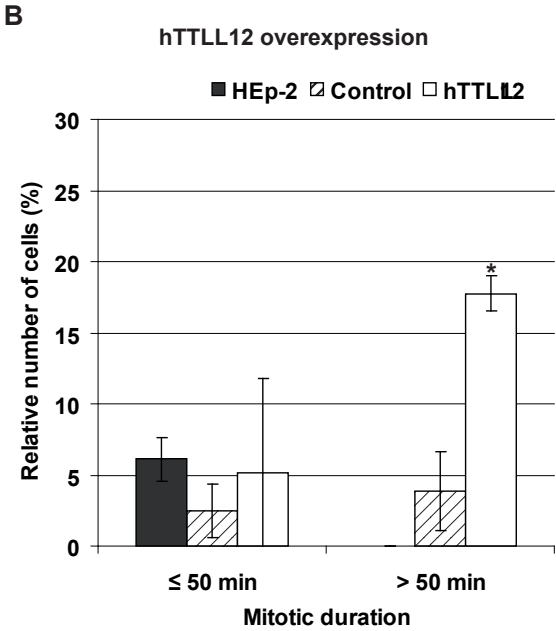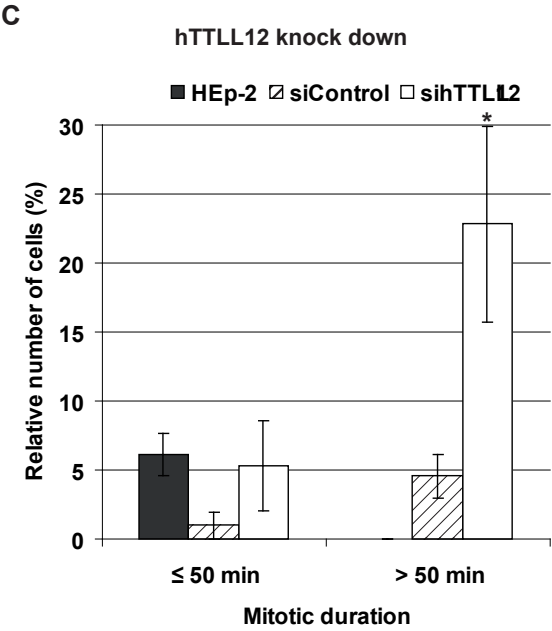

Supplement: Figure S6 — hTTLL12 up and down regulation leads to a spindle positioning phenotype in HEp-2 cells. The orientation and placement of mitotic spindles was scored from time-lapse movies (Experimental Procedures). The numbers of cells analysed were: 262 HEp-2, 420 Control_A-E, 380 hTTLL12_A, _B, _D clones, 502 control siRNAs (siCtrl, siLuciferase, siScramble, used separately and combined), and 973 sihTTLL12 (sihTTLL12_1-6, used separately). A. Phase contrast and fluorescent (Hoechst) image overlays of metaphases from representative cells: a HEp-2 (Control, upper panel), a hTTLL12 clone (hTTLL12, middle panel) and a sihTTLL12 transfected cell (sihTTLL12, lower panel). Red lines and grey circles highlight metaphase plate position with respect to the circumference of the cell. 0 min is the time of anaphase onset. B. Average percentages for each cell type ± STDEV with the spindle positioning phenotype divided into time intervals (mitotic duration ≤50 min or >50 min) for HEp-2 cells, hTTLL12 and Control clones. * Statistically significant (P<0.05, Student's t-test) difference between hTTLL12 clones and Control clones or HEp-2 cells. C. Average percentage for each clone or cell type ± STDEV with the spindle positioning phenotype divided into time intervals (mitotic duration ≤50 min or >50 min) scored from time lapse movies with HEp-2 or cell transfected with control siRNAs or sihTTLL12s. * Statistically significant difference between sihTTLL12 and control siRNA transfected cells or HEp-2 (P<0.05, Student's t-test). (PDF) [file pone.0051258.s007.pdf]
