# Supplementary material for: Tubulin Tyrosine Ligase Like 12, a TTLL Family Member with SET- and TTL-Like Domains and Roles in Histone and Tubulin Modifications and Mitosis
Source: PLoS One. 2012 Dec 12;7(12):e51258. doi: 10.1371/journal.pone.0051258 (PMC3520985; doi:10.1371/journal.pone.0051258)
Supplement: Table S1 — Amino acid conservation in the TTL core domain of TTL family members. The consensus sequence for the human family and the corresponding sequence in hTTLL12 are from Alignment S1. The alignment for different species is not shown. (PDF) [file pone.0051258.s013.pdf]

Tables S1-3

Table S1. Amino acid conservation between TTL family members.

	**Consensus TTLs**	**hTTLL12**
Generally conserved, human TTL family	WIVK	WICK$^{416-419}$
	QKYI	SKYI$^{450-453}$
	DLR+	DIRY$^{470-473}$
	EVN	EVN$^{605-607}$
In human TTL family, other than TTLL12	GXGI	SLDT$^{426-429}$
	HLTN	HFTV$^{511-514}$
	FEψψGFD	RAMYAVD$^{578-584}$
Conserved in TTLL12s of different species (including plants)		PK$^{446-447}$
		W^{492}
		R^{578}
		C^{612}
		C^{616}
