# Supplementary material for: Tubulin Tyrosine Ligase Like 12, a TTLL Family Member with SET- and TTL-Like Domains and Roles in Histone and Tubulin Modifications and Mitosis
Source: PLoS One. 2012 Dec 12;7(12):e51258. doi: 10.1371/journal.pone.0051258 (PMC3520985; doi:10.1371/journal.pone.0051258)
Supplement: Table S2 — The sequences and their positions in hTTLL12 that are highly conserved in other human SET domains (see Alignment S2) are listed. Letters are coloured according to Fig. 1 in Qian et al. (38): red: absolutely conserved; blue: very highly conserved; black: highly conserved). The corresponding conserved structural elements are indicated (38). Amino acids 158–188 are the approximate limit of a predicted insert in the SET domain that is deduced from Alignment S2. Sequence alignments of hTTLL12 with several plant and unicellular ciliate protozoan orthologues (e.g. NP_177879.3, Arabidopsis thaliana; XP_002326123 Populus trichocarpa; ABF94289, Oryza sativa; XP_001020206, Tetrahymena thermophila SB210; XP_001454840, Paramecium tetraurelia strain d4-2; XP_001580810, Trichomonas vaginalis G3) indicate that these orthologues have an insert that has an additional sequence inserted in this position (data not shown). (PDF) [file pone.0051258.s008.pdf]

Table S2. Conserved sequences and corresponding potential structural element

Sequence in hTTLL12	Position in hTTLL12	Structure*
G	94	
V**I**	101-102	β1
G**L**	108-109	β2
**FL**ID	118-121	β3
E**F**H	152-154	β6
P-M	158-188***	insert
F	197	α2
HxxxP	203-207	α2-β8
RD	230-231	β9-β10
GEEV	235-238	β10

The sequences and their positions in hTTLL12 that are highly conserved in other human SET domains (see Alignment S2) are listed. Letters are coloured according to Figure 1 in Qian et al. {Qian, 2006 #87}: red: absolutely conserved; blue: very highly conserved; black: highly conserved). The corresponding conserved structural element are indicated {Qian, 2006 #87}. Amino acids 158-188 is the approximate limit of a predicted insert in the SET domain, that is deduced from Alignment S2. Sequence alignments of hTTLL12 with several plant and unicellular ciliate protozoan orthologues (e.g. NP_177879.3, Arabidopsis thaliana; XP_002326123 Populus trichocarpa; ABF94289, Oryza sativa; XP_001020206, Tetrahymena thermophila SB210; XP_001454840, Paramecium tetraurelia strain d4-2] XP_001580810, Trichomonas vaginalis G3). These alignments indicate that these orthologues have an insert that have an additional sequence inserted in this position (data not shown).
