# Supplementary material for: Tubulin Tyrosine Ligase Like 12, a TTLL Family Member with SET- and TTL-Like Domains and Roles in Histone and Tubulin Modifications and Mitosis
Source: PLoS One. 2012 Dec 12;7(12):e51258. doi: 10.1371/journal.pone.0051258 (PMC3520985; doi:10.1371/journal.pone.0051258)
Supplement: Table S3 — The 55 kDa co-immunoprecipitated protein band ( Fig. 3C ) corresponds to α-tubulin. The table summarizes tryptic peptide sequences, their relative positions on the α-tubulin primary protein sequence, their measured masses and computed masses. * oxidized methionine. (PDF) [file pone.0051258.s009.pdf]

Table S3

Peptide sequence	AA Position		Measured mass	Computed mass
	NH_2	COOH	Da	Da
AVFVDLEPTVIDEVR	65	79	1701.006	1700.898
QLFHPEQLITGK	85	96	1409.837	1409.766
QLFHPEQLITGKEDAANNYAR	85	105	2414.350	2414.197
EDAANNYAR	97	105	1022.481	1022.441
EIIDLVLDR	113	121	1084.652	1084.612
LSVDYGKK	157	164	908.511	908.496
LISQIVSSITASLR	230	243	1486.907	1486.871
FDGALNVDLTEFQTNLVPYPR	244	264	2408.251	2408.200
YMACCLLYR	312	320	1248.620	1248.545
DVNAAIATIK	327	336	1014.589	1014.570
TIQFVDWCPTGFK	340	352	1597.795	1597.759
LDHKFDLMYAK	391	401	1379.771	1379.690
LDHKFDL*MYAK	391	401	1395.764	1395.685
FDLMYAK	395	401	886.459	886.425
AFVHWYVGEGMEEGEFSEAR	403	422	2329.180	2329.010
