# Supplementary material for: Tubulin Tyrosine Ligase Like 12, a TTLL Family Member with SET- and TTL-Like Domains and Roles in Histone and Tubulin Modifications and Mitosis
Source: PLoS One. 2012 Dec 12;7(12):e51258. doi: 10.1371/journal.pone.0051258 (PMC3520985; doi:10.1371/journal.pone.0051258)
Supplement: Alignment S1 — Multiple sequence alignment of the TTL domains of the human TTL protein family members. (PDF) [file pone.0051258.s011.pdf]

ALIGNMENT S1

```
                           10        20        30        40        50
                   ....|....|....|....|....|....|....|....|....|....|..
TTL/1-377          ----------------------------------------------------
TTLL1/1-423        ----------------------------------------------------
TTLL2/1-592        ----------------------------------------------------
TTLL3/1-352        ----------------------------------------------------
TTLL4/1-1199       ----------------------------------------------------
TTLL5/1-1277       ----------------------------------------------------
TTLL6/1-569        ----------------------------------------------------
TTLL7/1-957        ----------------------------------------------------
TTLL8/1-834        ----------------------------------------------------
TTLL9/1-347        ----------------------------------------------------
TTLL10/1-673       ----------------------------------------------------
TTLL11/1-538       ----------------------------------------------------
TTLL12/1-644       --MEAERGPERRPAERSSPGQTPEEGAQALAEFAALHGPALRASGVPERYWG
TTLL13/1-459       ----------------------------------------------------

Conservation
                   ----------------------------------------------------

Quality

Consensus
                   ----------------------------------------------------
```

```
                      60        70        80        90        100
TTL/1-377       ----------------------------------------------------
TTLL1/1-423     ----------------------------------------------------
TTLL2/1-592     ----------------------------------------------------
TTLL3/1-352     ----------------------------------------------------
TTLL4/1-1199    ---------------------------------------------MASAGTQ
TTLL5/1-1277    ----------------------------------------------------
TTLL6/1-569     ----------------------------------------------------
TTLL7/1-957     ----------------------------------------------------
TTLL8/1-834     ----------------------------------------------------
TTLL9/1-347     ----------------------------------------------------
TTLL10/1-673    ----------------------------------------------------
TTLL11/1-538    ----------------------------------------------------
TTLL12/1-644    RLLHKLEHEVFDAGEVFGIMQVEEVEEEE--DEAAREVRKQQPNPGNELCYK
TTLL13/1-459    ----------------------------------------------------

Conservation
                ----------------------------------------------------
```

Quality


```
Consensus
                ----------------------------------------------------
```

```
                110       120       130       140       150
TTL/1-377      ----------------------------------------------------
TTLL1/1-423    ----------------------------------------------------
TTLL2/1-592    ----------------------------------------------------
TTLL3/1-352    ----------------------------------------------------
TTLL4/1-1199   HYSIGLRQKNSFKQSGPSGTVPATPPEKPSEGRVWPQAHQQVKPIWKLEKKQ
TTLL5/1-1277   ----------------------------------------------------
TTLL6/1-569    ----------------------------------------------------
TTLL7/1-957    ----------------------------------------------------
TTLL8/1-834    ----------------------------------------------------
TTLL9/1-347    ----------------------------------------------------
TTLL10/1-673   ----------------------------------------------------
TTLL11/1-538   ----------------------------------------------------
TTLL12/1-644   VIVTRESGLQAAHPNSIFLIDHAWTCRVEHARQQ------------------
TTLL13/1-459   ----------------------------------------------------

Conservation
               ----------------------------------------------------

Quality

Consensus
               ----------------------A-----------------------------
```

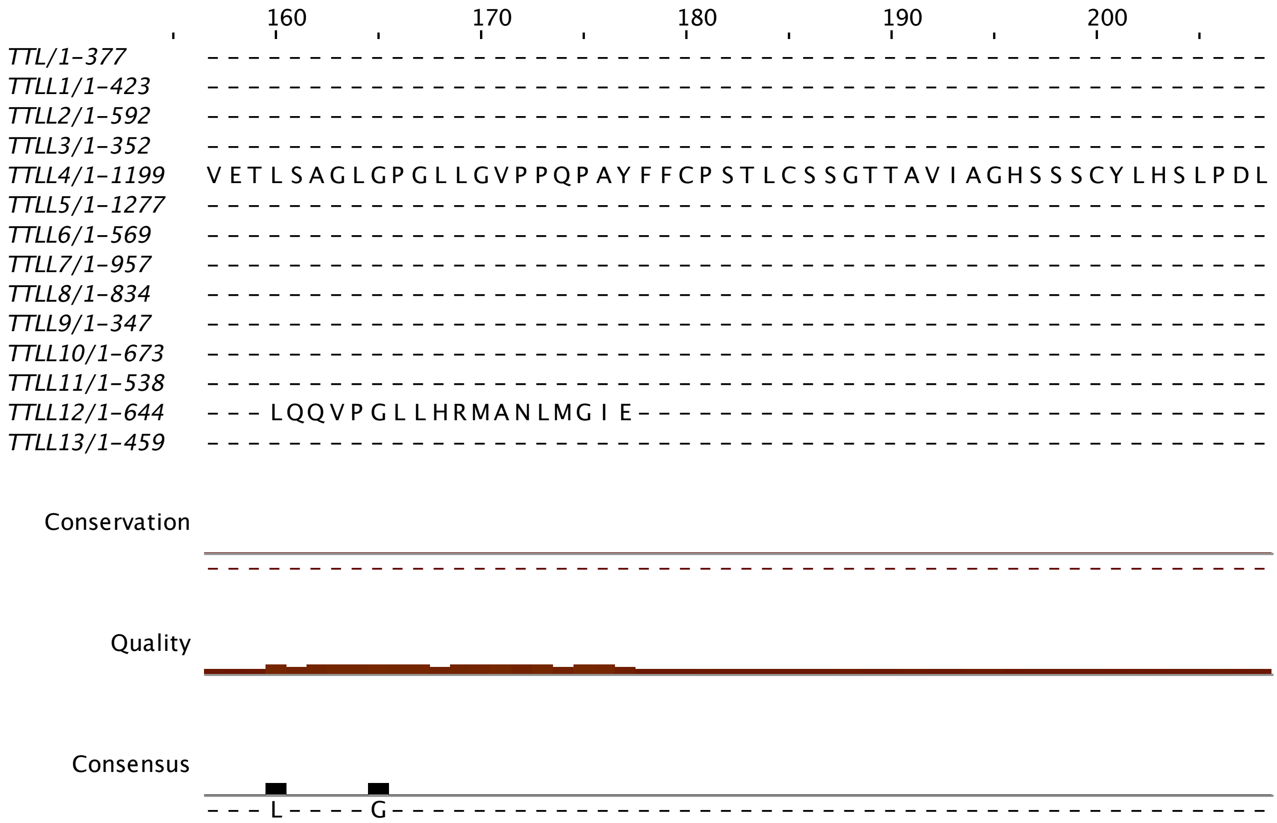
160 170 180 190 200
TTL/1-377 --
TTLL1/1-423 --
TTLL2/1-592 --
TTLL3/1-352 --
TTLL4/1-1199 VETLSAGLGPGLLGVPPQPAYFFCPSTLCSSGTTAVIAGHSSSCYLHSLPDL
TTLL5/1-1277 --
TTLL6/1-569 --
TTLL7/1-957 --
TTLL8/1-834 --
TTLL9/1-347 --
TTLL10/1-673 --
TTLL11/1-538 --
TTLL12/1-644 ---LQQVPGLLHRMANLMGIE-------------------------------
TTLL13/1-459 --
Conservation

Quality
Consensus
---L----G---

```
                 210       220       230       240       250
TTL/1-377        ----------------------------------------------------
TTLL1/1-423      ----------------------------------------------------
TTLL2/1-592      ----------------------------------------------------
TTLL3/1-352      ----------------------------------------------------
TTLL4/1-1199     FNSTLLYRRSSYRQKPYQQLESFCLRSSPSEKSPFSLPQKSLPVSLTANKAT
TTLL5/1-1277     ----------------------------------------------------
TTLL6/1-569      ----------------------------------------------------
TTLL7/1-957      ----------------------------------------------------
TTLL8/1-834      ----------------------------------------------------
TTLL9/1-347      ----------------------------------------------------
TTLL10/1-673     ----------------------------------------------------
TTLL11/1-538     ----------------------------------------------------
TTLL12/1-644     ----------------------------------------------------
TTLL13/1-459     ----------------------------------------------------

Conservation
                 ----------------------------------------------------

Quality

Consensus
                 ----------------------------------------------------
```

```
                270       280       290       300       310
TTL/1-377       ----------------------------------------------------
TTLL1/1-423     ----------------------------------------------------
TTLL2/1-592     ----------------------------------------------------
TTLL3/1-352     ----------------------------------------------------
TTLL4/1-1199    SSMVFSMAQPMASSSTEPYLCLAAAGENPSGKSLASAISGKIPSPLSSSYKP
TTLL5/1-1277    ----------------------------------------------------
TTLL6/1-569     ----------------------------------------------------
TTLL7/1-957     ----------------------------------------------------
TTLL8/1-834     ----------------------------------------------------
TTLL9/1-347     ----------------------------------------------------
TTLL10/1-673    ----------------------------------------------------
TTLL11/1-538    ----------------------------------------------------
TTLL12/1-644    ----------------------------------------------------
TTLL13/1-459    ----------------------------------------------------

Conservation
                ----------------------------------------------------

Quality

Consensus
                ----------------------------------------------------
```

```
                       320       330       340       350       360
TTL/1-377     ----------------------------------------------------
TTLL1/1-423   ----------------------------------------------------
TTLL2/1-592   ----------------------------------------------------
TTLL3/1-352   ----------------------------------------------------
TTLL4/1-1199  MLNNNSFMWPNSTPVPLLQTTQGLKPVSPPKIQPVSWHHSGGTGDCAPQPVD
TTLL5/1-1277  ----------------------------------------------------
TTLL6/1-569   ----------------------------------------------------
TTLL7/1-957   ----------------------------------------------------
TTLL8/1-834   ----------------------------------------------------
TTLL9/1-347   ----------------------------------------------------
TTLL10/1-673  ----------------------------------------------------
TTLL11/1-538  ----------------------------------------------------
TTLL12/1-644  ----------------------------------------------------
TTLL13/1-459  ----------------------------------------------------

Conservation
              ----------------------------------------------------

Quality

Consensus
              ----------------------------------------------------
```

```
                     370       380       390       400       410
TTL/1-377      ----------------------------------------------------
TTLL1/1-423    ----------------------------------------------------
TTLL2/1-592    ----------------------------------------------------
TTLL3/1-352    ----------------------------------------------------
TTLL4/1-1199   HKVPKSIGTVPADASAHIALSTASSHDTSTTSVASSWYNRNNLAMRAEPLSC
TTLL5/1-1277   ----------------------------------------------------
TTLL6/1-569    ----------------------------------------------------
TTLL7/1-957    ----------------------------------------------------
TTLL8/1-834    ----------------------------------------------------
TTLL9/1-347    ----------------------------------------------------
TTLL10/1-673   ----------------------------------------------------
TTLL11/1-538   ----------------------------------------------------
TTLL12/1-644   ----------------------------------------------------
TTLL13/1-459   ----------------------------------------------------

Conservation
               ----------------------------------------------------

Quality

Consensus
               ----------------------------------------------------
```

```
                420       430       440       450       460
TTL/1-377      ----------------------------------------------------
TTLL1/1-423    ----------------------------------------------------
TTLL2/1-592    ----------------------------------------------------
TTLL3/1-352    ----------------------------------------------------
TTLL4/1-1199   ALDDSSDSQDPTKEIRFTEAVRKLTARGFEKMPRQGCQLEQSSFLNPSFQWN
TTLL5/1-1277   ----------------------------------------------------
TTLL6/1-569    ----------------------------------------------------
TTLL7/1-957    ----------------------------------------------------
TTLL8/1-834    ----------------------------------------------------
TTLL9/1-347    ----------------------------------------------------
TTLL10/1-673   ----------------------------------------------------
TTLL11/1-538   ----------------------------------------------------
TTLL12/1-644   ----------------------------------------------------
TTLL13/1-459   ----------------------------------------------------

Conservation
               ----------------------------------------------------

Quality

Consensus
               ----------------------------------------------------
```

```
                 470       480       490       500       510
TTL/1-377        ----------------------------------------------------
TTLL1/1-423      ----------------------------------------------------
TTLL2/1-592      ----------------------------------------------------
TTLL3/1-352      ----------------------------------------------------
TTLL4/1-1199     VLNRSRRWKPPAVNQQFPQEDAGSVRRVLPGASDTLGLDNTVFCTKRISIHL
TTLL5/1-1277     ----------------------------------------------------
TTLL6/1-569      ----------------------------------------------------
TTLL7/1-957      ----------------------------------------------------
TTLL8/1-834      ----------------------------------------------------
TTLL9/1-347      ----------------------------------------------------
TTLL10/1-673     ----------------------------------------------------
TTLL11/1-538     -MAAAASVTGRVTWAASPMRSLGLGRRLSLPGPRLDAVTAAVNPSLSDHGNG
TTLL12/1-644     ----------------------------------------------------
TTLL13/1-459     ----------------------------------------------------

Conservation
                 ----------------------------------------------------

Quality

Consensus
                 -----------------P----G--RR--------------V----------
```

```
                  530       540       550       560       570
TTL/1-377      ----------------------------------------------------
TTLL1/1-423    ----------------------------------------------------
TTLL2/1-592    ----------------------------------------------------
TTLL3/1-352    ----------------------------------------------------
TTLL4/1-1199   LASH---ASGLNHNPACESVIDSSAFGEGKAPGPPFPQTLGIANVATRLSSI
TTLL5/1-1277   ----------------------------------------------------
TTLL6/1-569    ----------------------------------------------------
TTLL7/1-957    ----------------------------------------------------
TTLL8/1-834    ----------------------------------------------------
TTLL9/1-347    ----------------------------------------------------
TTLL10/1-673   -----------------------------------------MDHSCTRFIHR
TTLL11/1-538   LGRGTRGSGCSGGSLVADWGGGAAAAAAVALALAPALSTMRRGSSESELAAR
TTLL12/1-644   --------FHGELPSTEAVALVLEEMWKFNQTYQLAHG---TAEEKMPVWYI
TTLL13/1-459   ----------------------------------------------------

Conservation

Quality

Consensus      L-----------------------A---------PA--T---A-S-TRL--+
```

```
                    580       590       600       610       620
TTL/1-377      ----------------------------------------------------
TTL1/1-423     ----------------------------------------------------
TTL2/1-592     ----------------------------------------------------
TTL3/1-352     ----------------------------------------------------
TTL4/1-1199    QLGQSEKERPEEARELDSSDRDISSATDLQPDQAETEDTEEELVDGLEDCCS
TTL5/1-1277    ----------------------------------------------------
TTL6/1-569     ----------------------------------------------------
TTL7/1-957     ----------------MPSLPQEGVIQGPSPLDLNTELPYQSTMKRKVR--K
TTL8/1-834     ----------------------------------------------------
TTL9/1-347     ----------------------------------------------------
TTL10/1-673    RGPPTRTRAGFKRGKRPRIQQRPRARVSGTIPASRLHPAPASQPGPCPAPGH
TTL11/1-538    WEAEAVAAAKAAAKAEAEATAETVAEQVRVDAGAAGEPECKAGEEQPKVLAP
TTL12/1-644    MDEFGSRIQ-HADVPSFAT-APF-FYMPQQVA-----YTLLWPLRDLDTGEE
TTL13/1-459    -------MEPSTCRTMESEEDYVEEKESEKCVKEGVTNPSNSSQQALLKADY
```

Conservation

Quality

Consensus

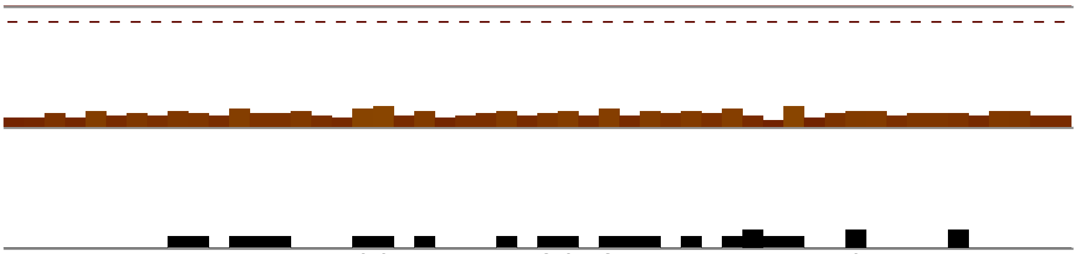

```
               --------AP-AAR---SS-A---A-QS-QPA-A-TEP+--S----L-----
```

```
                 630       640       650       660       670
TTL/1-377       ----------------------------------------------------
TTLL1/1-423     ----------------------------------------------------
TTLL2/1-592     --------MRGRDLCSSTQSQALGSLRTTTPAFTLNIPSEANHTEQPPAGLG
TTLL3/1-352     ----------------------------------------------------
TTLL4/1-1199    RDENEEEEGDSECSSLSAVSP-----SESVAMISRSCMEILTKPLSNHEKVV
TTLL5/1-1277    -------------------------------------MARDLEETASSSEDE
TTLL6/1-569     ----------------------------------------------------
TTLL7/1-957     KKKKGTITANVAGTKFEIVRLVIDEMGFMKTPDEDE----------------
TTLL8/1-834     ----------------------------------------------------
TTLL9/1-347     ----------------------------------------------------
TTLL10/1-673    CPVGPAHERPMGSSQEEGLRCQPSQPDHDADGHCGPDLEGAERASATPGPPG
TTLL11/1-538    APAQPS--AA-EEGNTQVLQRPPPTLPPSKPKPVQGLCPHGKPRDKGRSCKR
TTLL12/1-644    VTRDFAYGET---DPLIRKC-----MLLPWAPTDMLDLSSCT------PEPP
TTLL13/1-459    KALKNGVPS-PIMATKIPKKVIAPVDTGDLEAGRRKRRRKRRSLAINLTNC-

Conservation
                ----------------------------------------------------
Quality
```

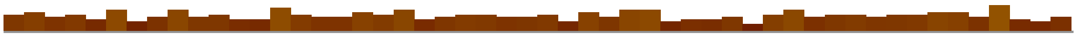

```
Consensus
                KP-KPA-EA--E-S-L+-++--PP-+--+K++--R-D++-AT---ANPSEPG
```

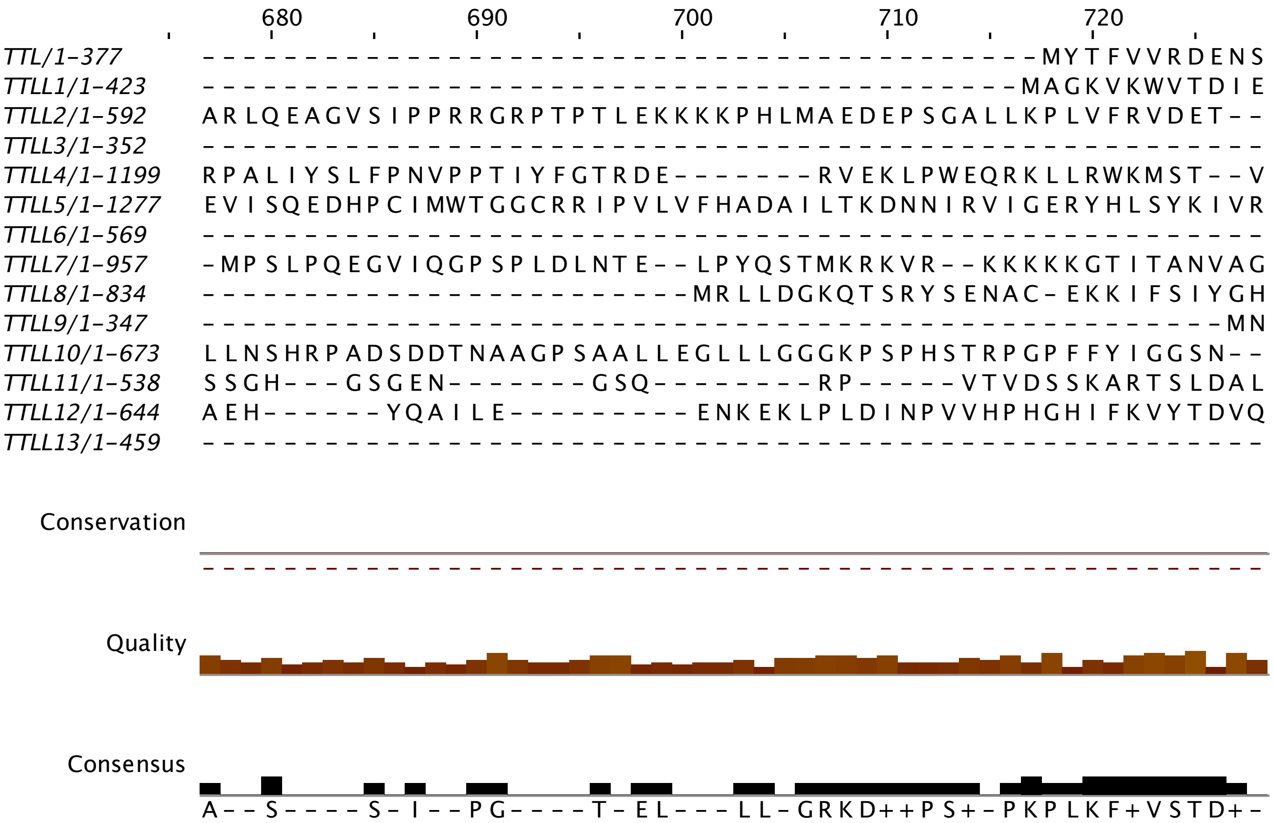
680 690 700 710 720
TTL/1-377 ---MYTFVVRDENS
TTLL1/1-423 --MAGKVKWVTDIE
TTLL2/1-592 ARLQEAGVSIPPRRGRPTPTLEKKKKPHLMAEDEPSGALLKPLVFRVDET--
TTLL3/1-352 --
TTLL4/1-1199 RPALIYSLFPNVPPTIYFGTRDE-------RVEKLPWEQRKLLRWKMST--V
TTLL5/1-1277 EVISQEDHPCIMWTGGCRRIPVLVFHADAILTKDNNIRVIGERYHLSYKIVR
TTLL6/1-569 --
TTLL7/1-957 -MPSLPQEGVIQGPSPLDLNTE--LPYQSTMKRKVR--KKKKKGTITANVAG
TTLL8/1-834 ------------------------MRLLDGKQTSRYSENAC-EKKIFSIYGH
TTLL9/1-347 --MN
TTLL10/1-673 LLNSHRPADSDDTNAAGPSAALLEGLLLGGGKPSPHSTRPGPFFYIGGSN--
TTLL11/1-538 SSGH---GSGEN-------GSQ--------RP-----VTVDSSKARTSLDAL
TTLL12/1-644 AEH------YQAILE---------ENKEKLPLDINPVVHPHGHIFKVYTDVQ
TTLL13/1-459 --
Conservation
Quality
Consensus
A--S----S-I--PG----T-EL---LL-GRKD++PS+-PKPLKF+VSTD+-

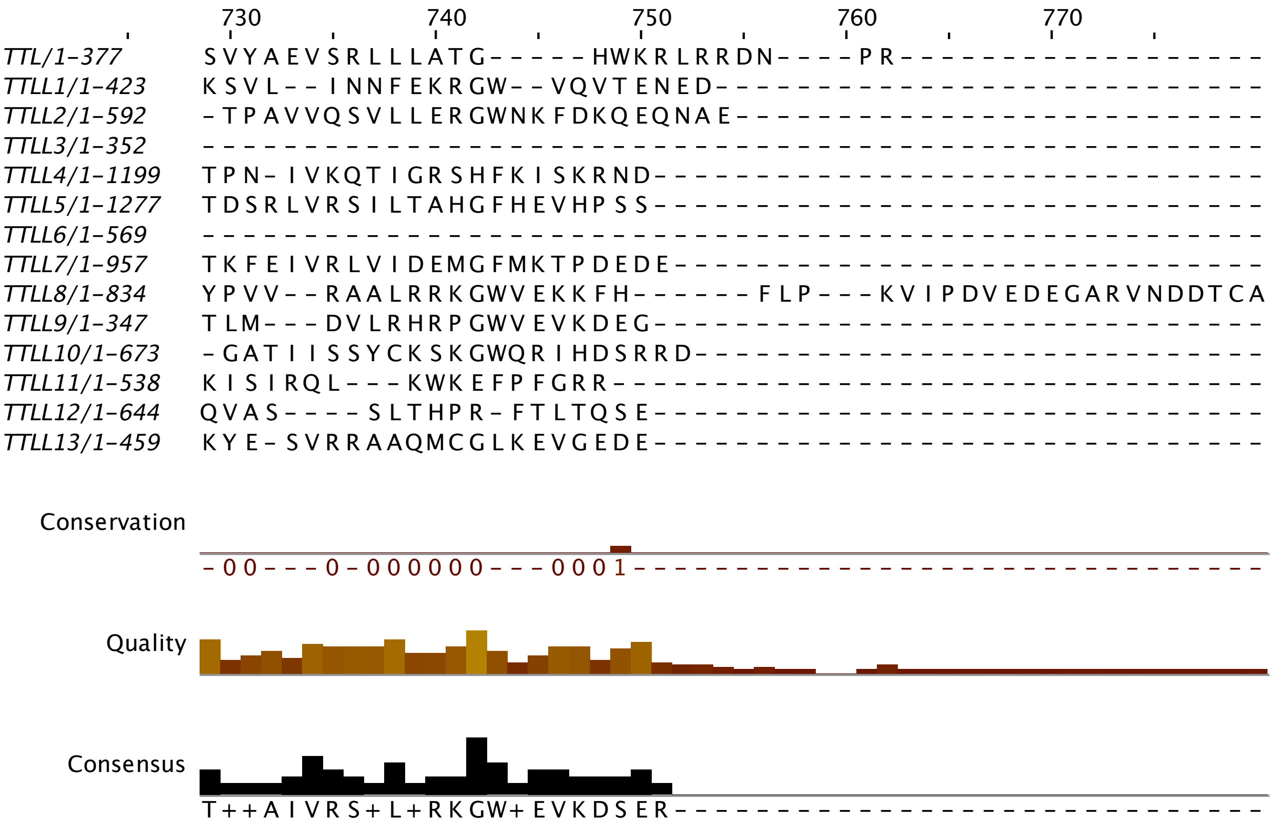
730 740 750 760 770
TTL/1-377 SVYAEVSRLLLATG-----HWKRLRRDN----PR------------------
TTLL1/1-423 KSVL--INNFEKRGW--VQVTENED---------------------------
TTLL2/1-592 -TPAVVQSVLLERGWNKFDKQEQNAE--------------------------
TTLL3/1-352 --
TTLL4/1-1199 TPN-IVKQTIGRSHFKISKRND------------------------------
TTLL5/1-1277 TDSRLVRSILTAHGFHEVHPSS------------------------------
TTLL6/1-569 --
TTLL7/1-957 TKFEIVRLVIDEMGFMKTPDEDE-----------------------------
TTLL8/1-834 YPVV--RAALRRKGWVEKKFH------FLP---KVIPDVEDEGARVNDDTCA
TTLL9/1-347 TLM---DVLRHRPGWVEVKDEG------------------------------
TTLL10/1-673 -GATIISSYCKSKGWQRIHDSRRD----------------------------
TTLL11/1-538 KISIRQL---KWKEFPFGRR--------------------------------
TTLL12/1-644 QVAS----SLTHPR-FTLTQSE------------------------------
TTLL13/1-459 KYE-SVRRAAQMCGLKEVGEDE------------------------------
Conservation
-00---0-000000---0001-------------------------------
Quality
Consensus
T++AIVRS+L+RKGW+EVKDSER-----------------------------

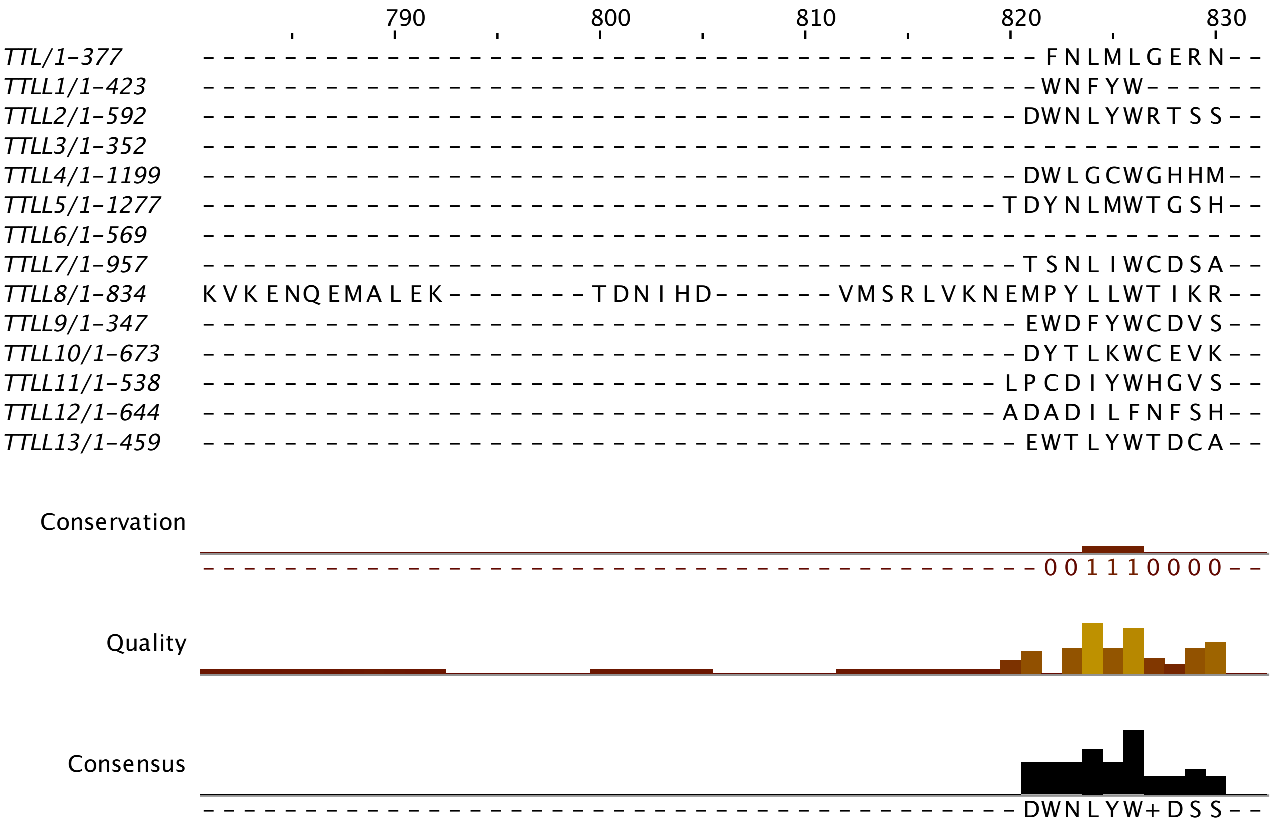
790
800
810
820
830
TTL/1-377 FNLMLGERN
TTL1/1-423 WNFYW
TTLL2/1-592 DWNLYWRTSS
TTLL3/1-352
TTLL4/1-1199 DWLGCWGHHM
TTLL5/1-1277 TDYNLMWTGSH
TTLL6/1-569
TTLL7/1-957 TSNLIWCDSA
TTLL8/1-834 KVKENQEMALEK TDNIHD VMSRLVKNEMPYLLWTIKR
TTLL9/1-347 EWDFYWCDVS
TTLL10/1-673 DYTLKWCEVK
TTLL11/1-538 LPCDIYWHGVS
TTLL12/1-644 ADADILFNFSH
TTLL13/1-459 EWTLYWTDCA
Conservation
001110000
Quality
Consensus
DWNLYW+DSS

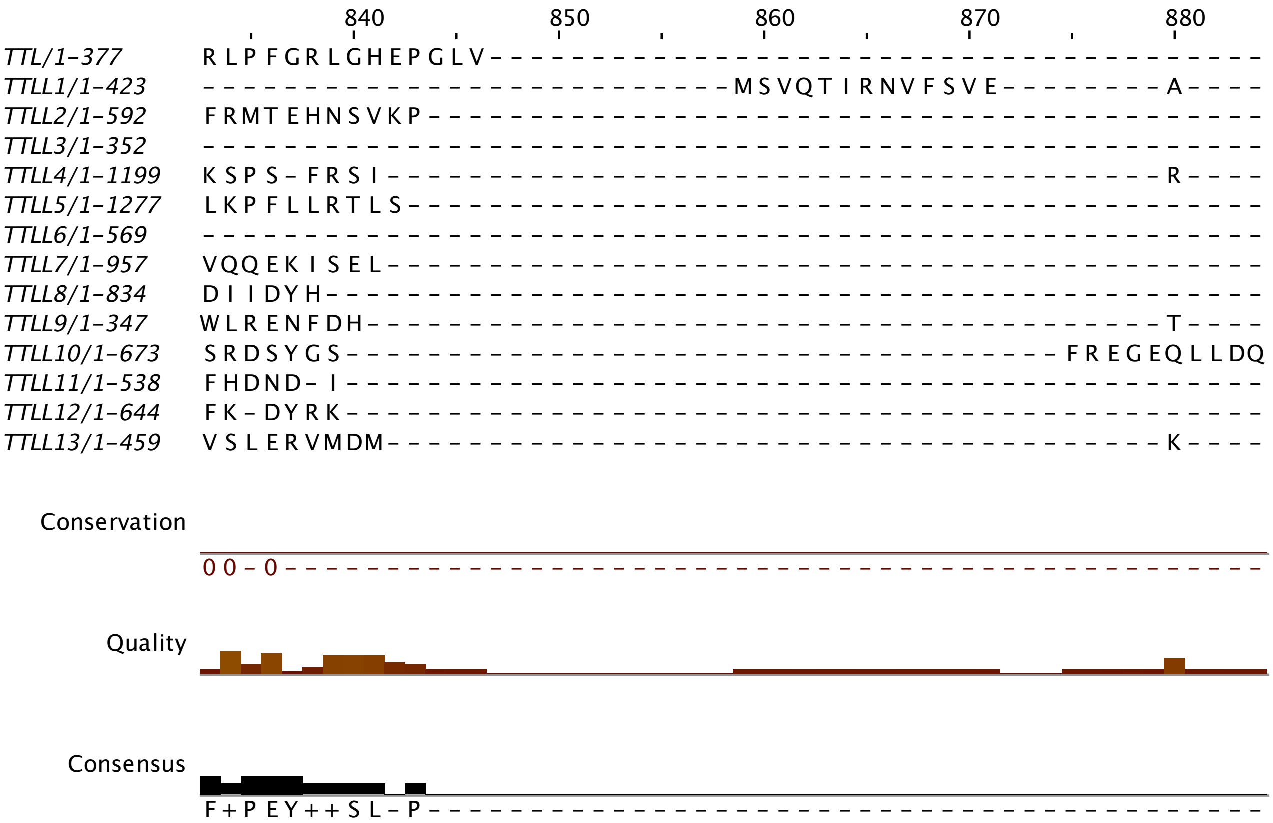
840 850 860 870 880
TTL/1-377 RLPFGRLGHEPGLV--------------------------------------
TTLL1/1-423 --------------------------MSVQTIRNVFSVE--------A----
TTLL2/1-592 FRMTEHNSVKP---
TTLL3/1-352 --
TTLL4/1-1199 KSPS-FRSI--------------------------------------R----
TTLL5/1-1277 LKPFLLRTLS--
TTLL6/1-569 --
TTLL7/1-957 VQQEKISEL---
TTLL8/1-834 DIIDYH--
TTLL9/1-347 WLRENFDH---------------------------------------T----
TTLL10/1-673 SRDSYGS-----------------------------------FREGEQLLDQ
TTLL11/1-538 FHDND-I---
TTLL12/1-644 FK-DYRK---
TTLL13/1-459 VSLERVMDM--------------------------------------K----
Conservation
00-0--
Quality
Consensus
F+PEY++SL-P---

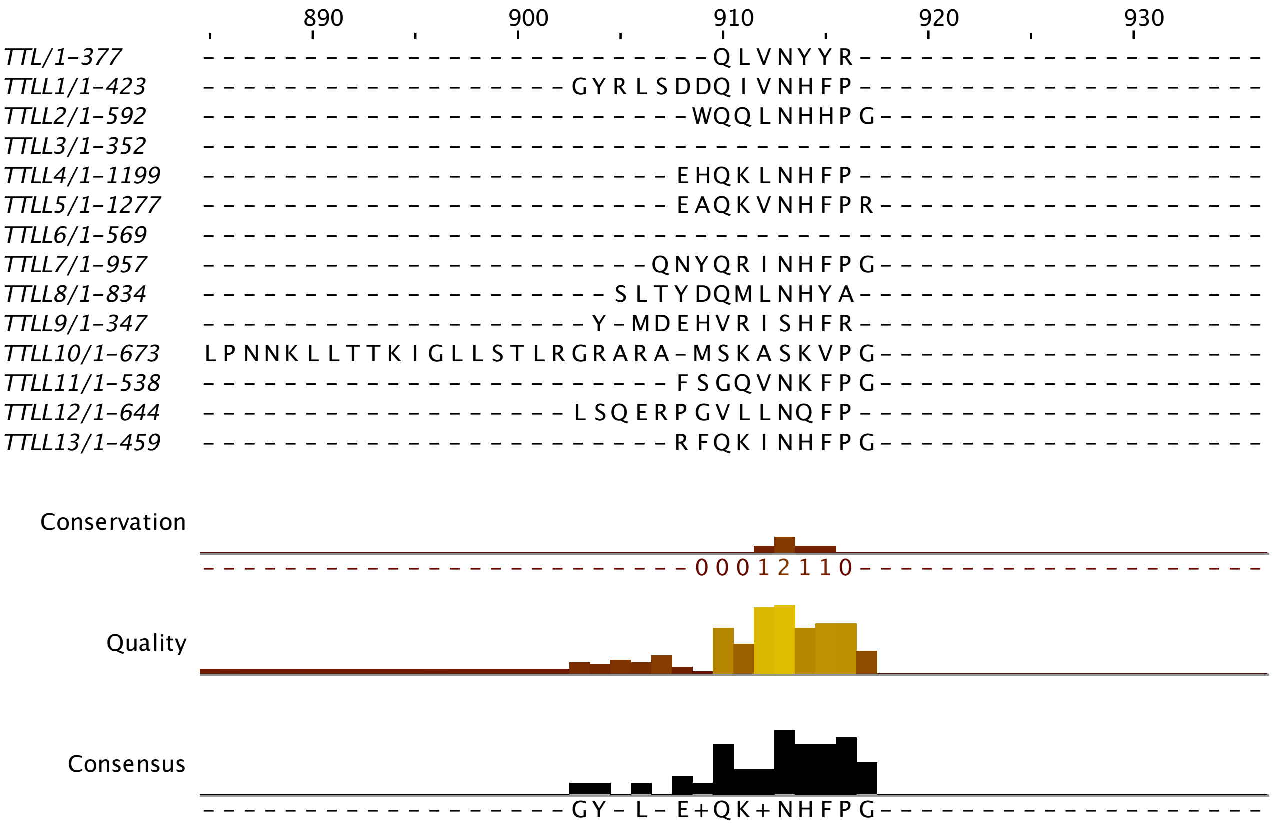
890 900 910 920 930
TTL/1-377 QLVNYYR
TTLL1/1-423 GYRLSDDQIVNHFP
TTLL2/1-592 WQQLNHHPG
TTLL3/1-352
TTLL4/1-1199 EHQKLNHFP
TTLL5/1-1277 EAQKVNHFPR
TTLL6/1-569
TTLL7/1-957 QNYQRINHFPG
TTLL8/1-834 SLTYDQMLNHYA
TTLL9/1-347 Y-MDEHVRISHFR
TTLL10/1-673 LPNNKLLTTKIGLLSTLRGRARA-MSKASKVPG
TTLL11/1-538 FSGQVNKFPG
TTLL12/1-644 LSQERPGVLLNQFP
TTLL13/1-459 RFQKINHFPG
Conservation
00012110
Quality
Consensus
GY-L-E+QK+NHFPG

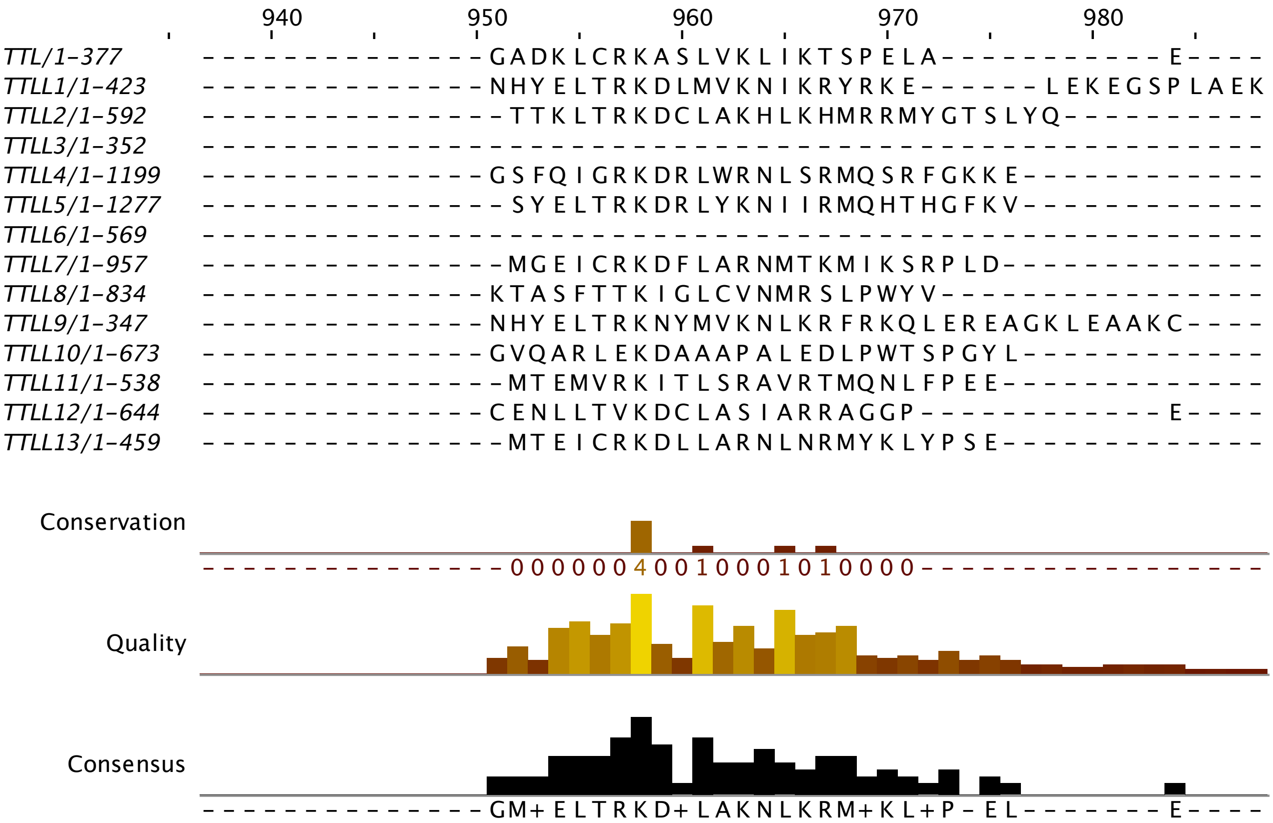
940 950 960 970 980
TTL/1-377 GADKLCRKASLVKLIKTSPELA E
TTLL1/1-423 NHYELTRKDLMVKNIKRYRKE LEKEGSPLAEK
TTLL2/1-592 TTKLTRKDCLAKHLKHMRRMYGTSLYQ
TTLL3/1-352
TTLL4/1-1199 GSFQIGRKDRLWRNLSRMQSRFGKKE
TTLL5/1-1277 SYELTRKDRLYKNIIRMQHTHGFKV
TTLL6/1-569
TTLL7/1-957 MGEICRKDFLARNMTKMIKSRPLD
TTLL8/1-834 KTASFTTKIGLCVNMRSLPWYV
TTLL9/1-347 NHYELTRKNYMVKNLKRFRKQLEREAGKLEAAKC
TTLL10/1-673 GVQARLEKDAAAPALEDLPWTSPGYL
TTLL11/1-538 MTEMVRKITLSRAVRTMQNLFPEE
TTLL12/1-644 CENLLTVKDCLASIARRAGGP E
TTLL13/1-459 MTEICRKDLLARNLNRMYKLYPSE
Conservation
00000040010001010000
Quality
Consensus
GM+ELTRKD+LAKNLKRM+KL+P-EL E

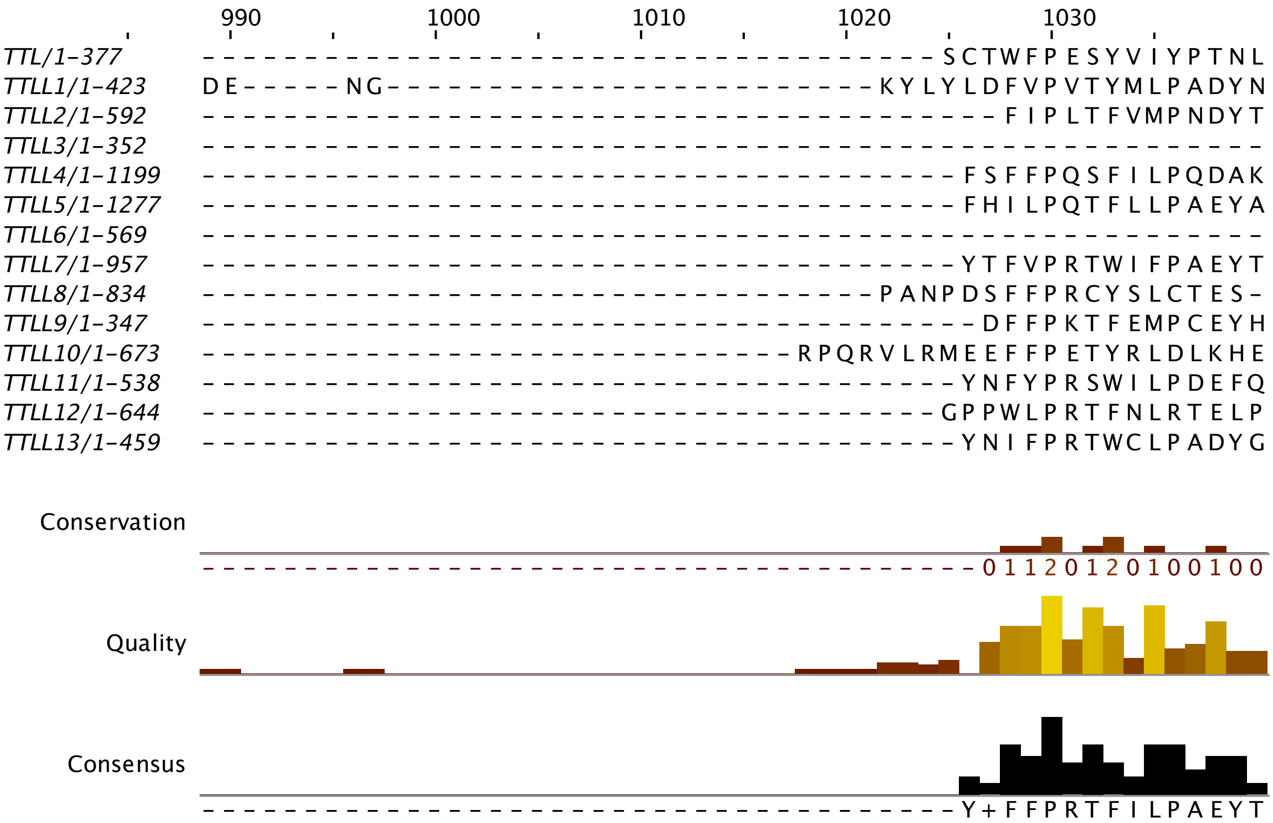
990
1000
1010
1020
1030
TTL/1-377 SCTWFPESYVIYPTNL
TTL1/1-423 DE NG KYLYLDFVPVTYMLPADYN
TTL2/1-592 FIPLTFVMPNDYT
TTL3/1-352
TTL4/1-1199 FSFFPQSFILPQDAK
TTL5/1-1277 FHILPQTFLLPAEYA
TTL6/1-569
TTL7/1-957 YTFVPRTWIFPAEYT
TTL8/1-834 PANPDSFFPRCYSLCTES
TTL9/1-347 DFFPKTFEMPCEYH
TTL10/1-673 RPQRVLRMEEFFPETYRLDLKHE
TTL11/1-538 YNFYPRSWILPDEFQ
TTL12/1-644 GPPWLPRTFNLRTELP
TTL13/1-459 YNIFPRTWCLPADYG
Conservation
0112012010010 0
Quality
Consensus
Y+FFPRTFILPAEYT

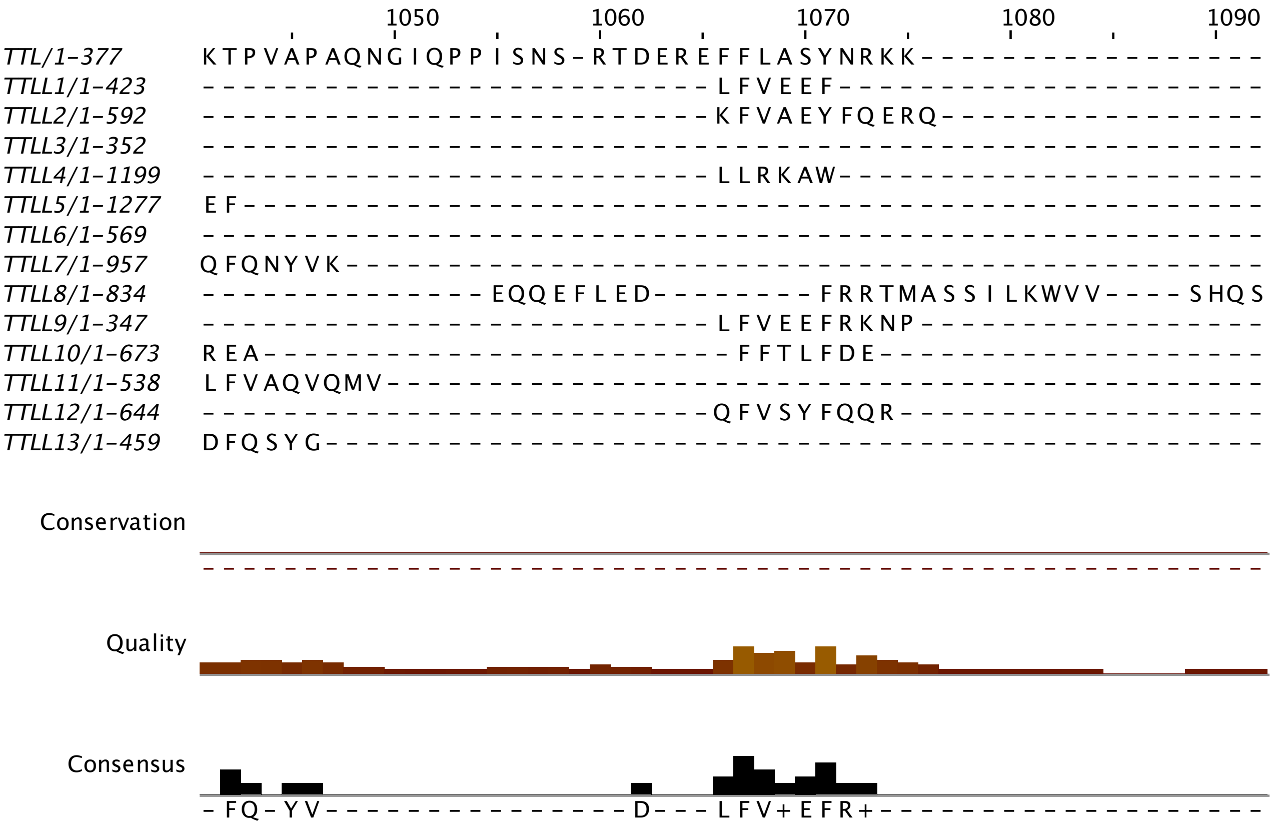
1050
1060
1070
1080
1090
TTL/1-377 KTPVAPAQNGIQPPISNS-RTDEREFFLASYNRKK------------------
TTLL1/1-423 -------------------------LFVEEF------------------------------
TTLL2/1-592 -------------------------KFVAEYFQERQ------------------------
TTLL3/1-352 --
TTLL4/1-1199 -------------------------LLRKAW------------------------------
TTLL5/1-1277 EF--
TTLL6/1-569 --
TTLL7/1-957 QFQNYVK---
TTLL8/1-834 --------------EQQEFLED--------FRRTMASSILKWVV----SHQS
TTLL9/1-347 -------------------------LFVEEFRKNP--------------------------
TTLL10/1-673 REA---------------------FFTLFDE---------------------
TTLL11/1-538 LFVAQVQMV---
TTLL12/1-644 -------------------------QFVSYFQQR--------------------------
TTLL13/1-459 DFQSYG--
Conservation
Quality
Consensus
-FQ-YV---------------D---LFV+EFR+------------------

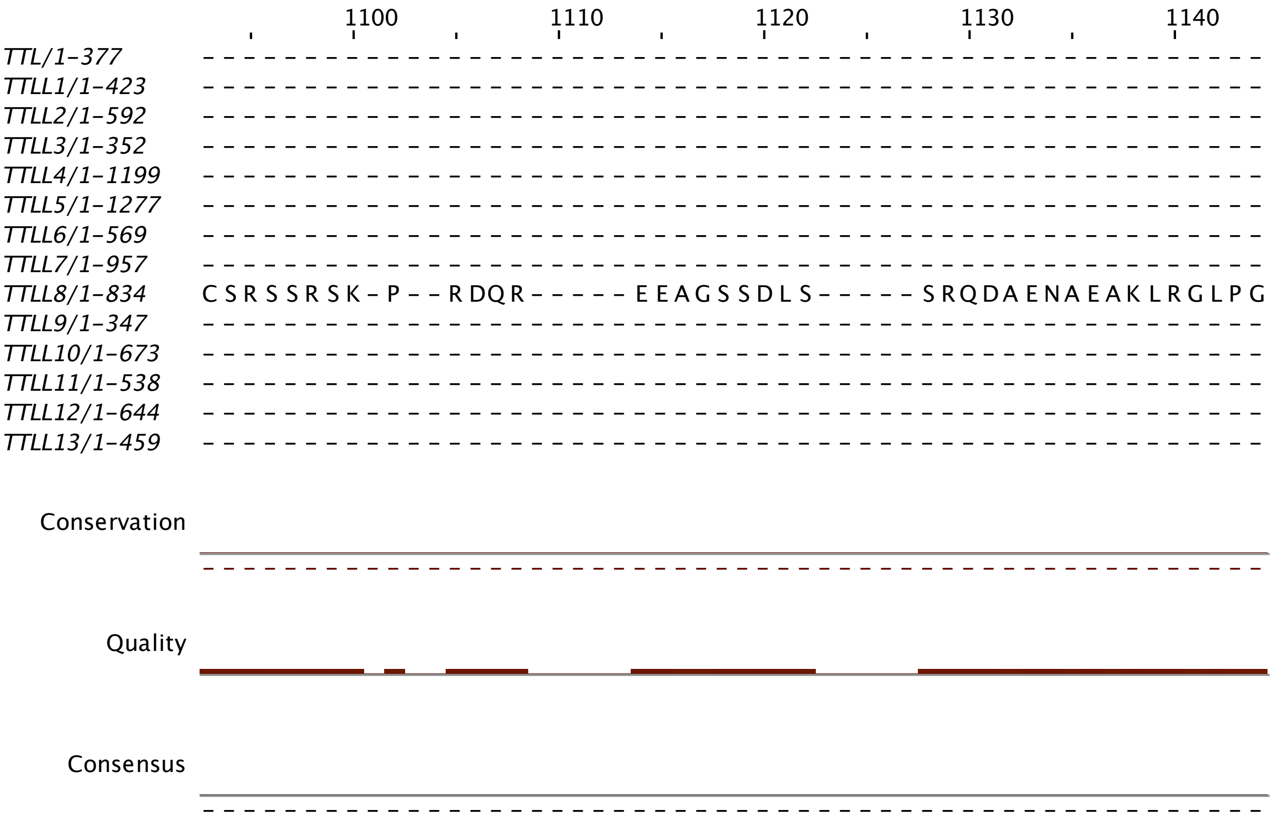

1100
1110
1120
1130
1140
TTL/1-377
TTLL1/1-423
TTLL2/1-592
TTLL3/1-352
TTLL4/1-1199
TTLL5/1-1277
TTLL6/1-569
TTLL7/1-957
TTLL8/1-834 CSRSSRSK-P--RDQR-----EEAGSSDLS-----SRQDAENAEAKLRGLPG
TTLL9/1-347
TTLL10/1-673
TTLL11/1-538
TTLL12/1-644
TTLL13/1-459
Conservation
Quality
Consensus

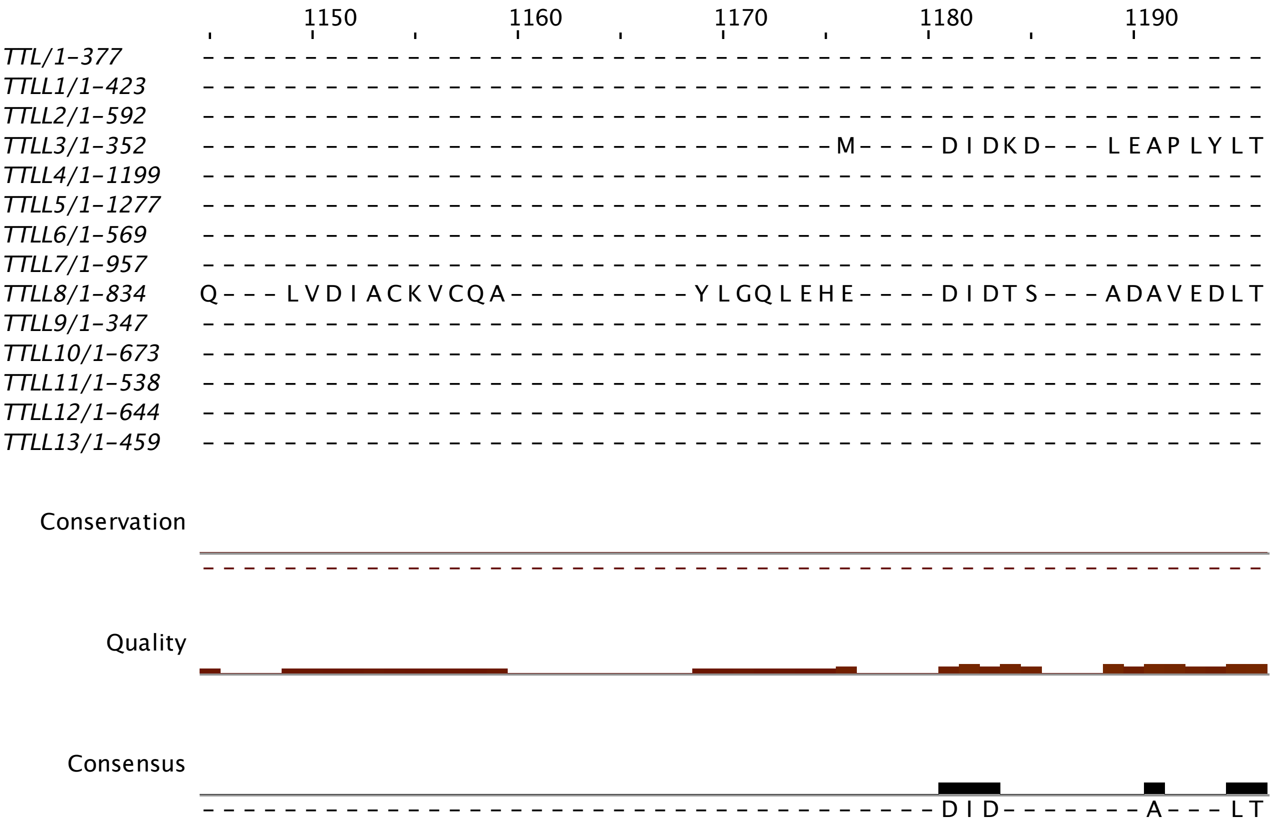
1150
1160
1170
1180
1190
TTL/1-377
TTLL1/1-423
TTLL2/1-592
TTLL3/1-352
TTLL4/1-1199
TTLL5/1-1277
TTLL6/1-569
TTLL7/1-957
TTLL8/1-834
TTLL9/1-347
TTLL10/1-673
TTLL11/1-538
TTLL12/1-644
TTLL13/1-459
------------------------------M----DIDKD---LEAPLYLT
Q---LVDIACKVCQA---------YLGQLEHE----DIDTS---ADAVEDLT
Conservation
Quality
Consensus
-----------------------------------DID-------A---LT

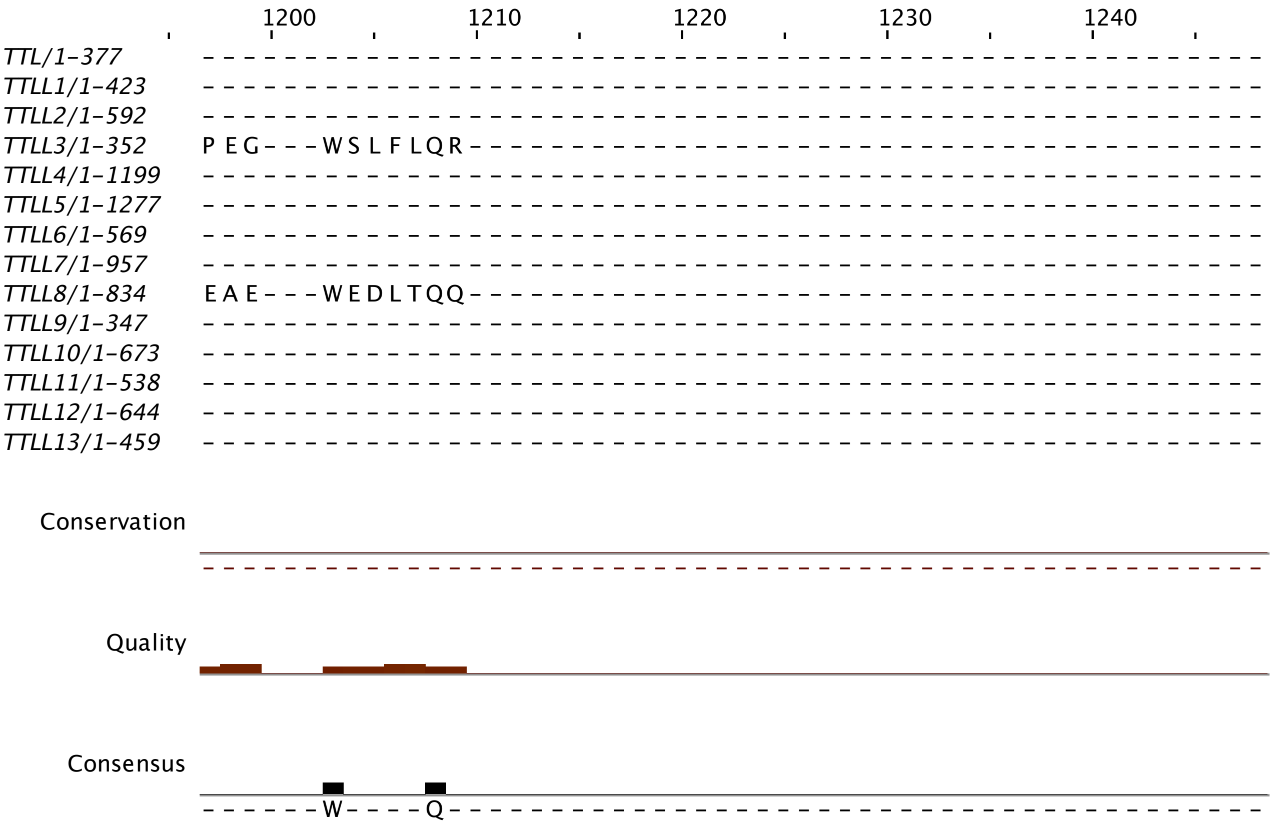
1200
1210
1220
1230
1240
TTL/1-377
TTLL1/1-423
TTLL2/1-592
TTLL3/1-352 PEG---WSLFLQR
TTLL4/1-1199
TTLL5/1-1277
TTLL6/1-569
TTLL7/1-957
TTLL8/1-834 EAE---WEDLTQQ
TTLL9/1-347
TTLL10/1-673
TTLL11/1-538
TTLL12/1-644
TTLL13/1-459
Conservation
Quality
Consensus
------W----Q

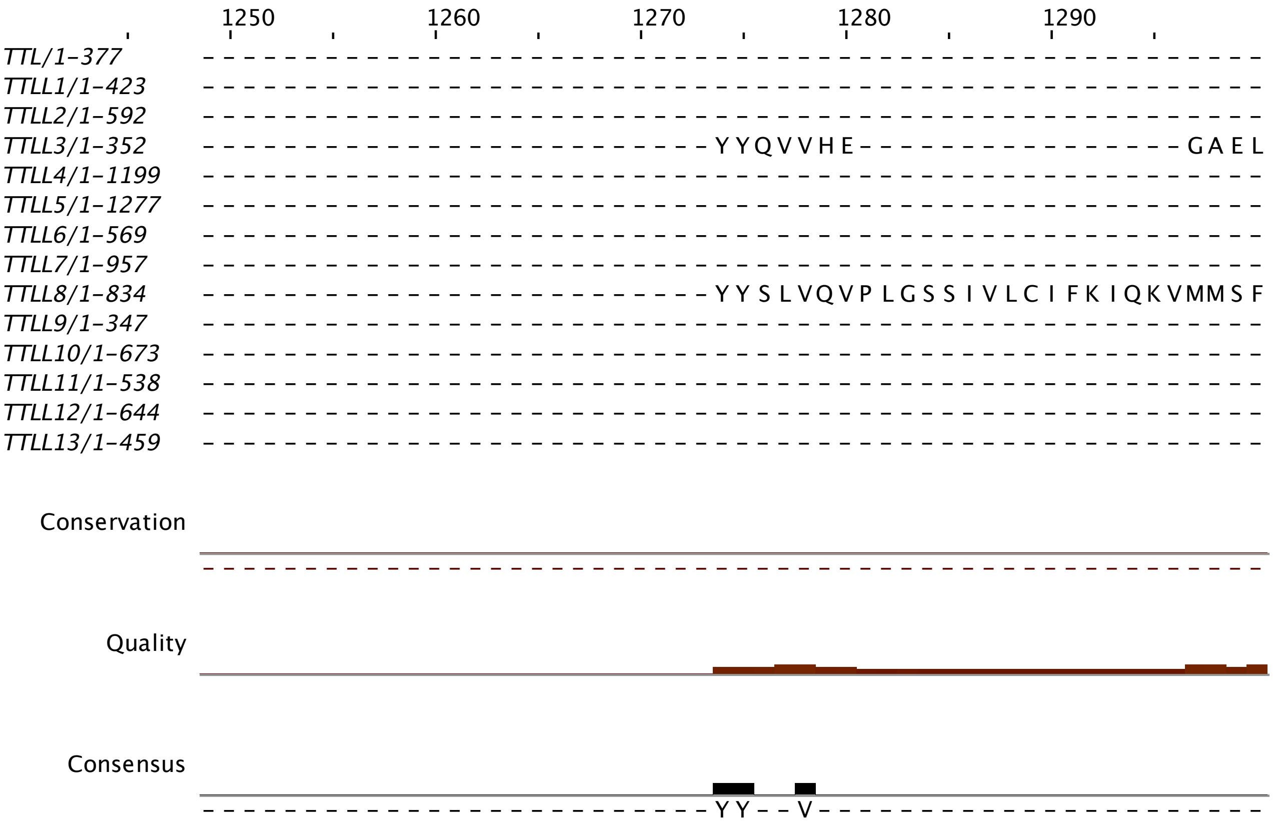
1250
1260
1270
1280
1290
TTL/1-377
TTLL1/1-423
TTLL2/1-592
TTLL3/1-352
YYQVVHE
GAEL
TTLL4/1-1199
TTLL5/1-1277
TTLL6/1-569
TTLL7/1-957
TTLL8/1-834
YYSLVQVPLGSSIVLCIFKIQKVMMSF
TTLL9/1-347
TTLL10/1-673
TTLL11/1-538
TTLL12/1-644
TTLL13/1-459
Conservation
Quality
Consensus
YY--V

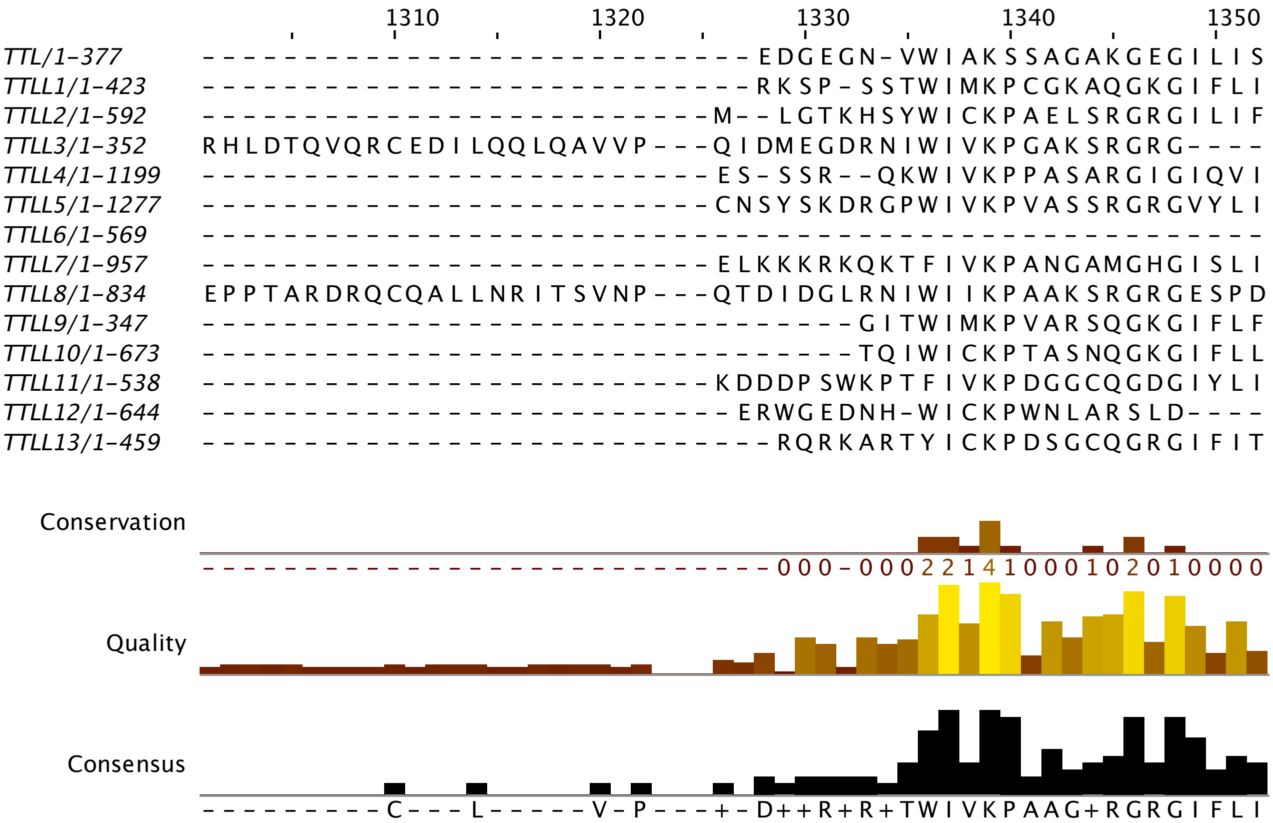
1310 1320 1330 1340 1350
TTL/1-377 ---------------------------EDGEGN-VWIAKSSAGAKGEGILIS
TTLL1/1-423 ---------------------------RKSP-SSTWIMKPCGKAQGKGIFLI
TTLL2/1-592 -------------------------M--LGTKHSYWICKPAELSRGRGILIF
TTLL3/1-352 RHLDTQVQRCEDILQQLQAVVP---QIDMEGDRNIWIVKPGAKSRGRG----
TTLL4/1-1199 -------------------------ES-SSR--QKWIVKPPASARGIGIQVI
TTLL5/1-1277 -------------------------CNSYSKDRGPWIVKPVASSRGRGVYLI
TTLL6/1-569 --
TTLL7/1-957 -------------------------ELKKKRKQKTFIVKPANGAMGHGISLI
TTLL8/1-834 EPPTARDRQCQALLNRITSVNP---QTDIDGLRNIWIIKPAAKSRGRGESPD
TTLL9/1-347 --------------------------------GITWIMKPVARSQGKGIFLF
TTLL10/1-673 --------------------------------TQIWICKPTASNQGKGIFLL
TTLL11/1-538 -------------------------KDDDPSWKPTFIVKPDGGCQGDGIYLI
TTLL12/1-644 --------------------------ERWGEDNH-WICKPWNLARSLD----
TTLL13/1-459 ----------------------------RQRKARTYICKPDSGCQGRGIFIT
Conservation
----------------------------000-000221410001020100000
Quality
Consensus
---------C---L-----V-P---+-D++R+R+TWIVKPAAG+RGRGIFLI

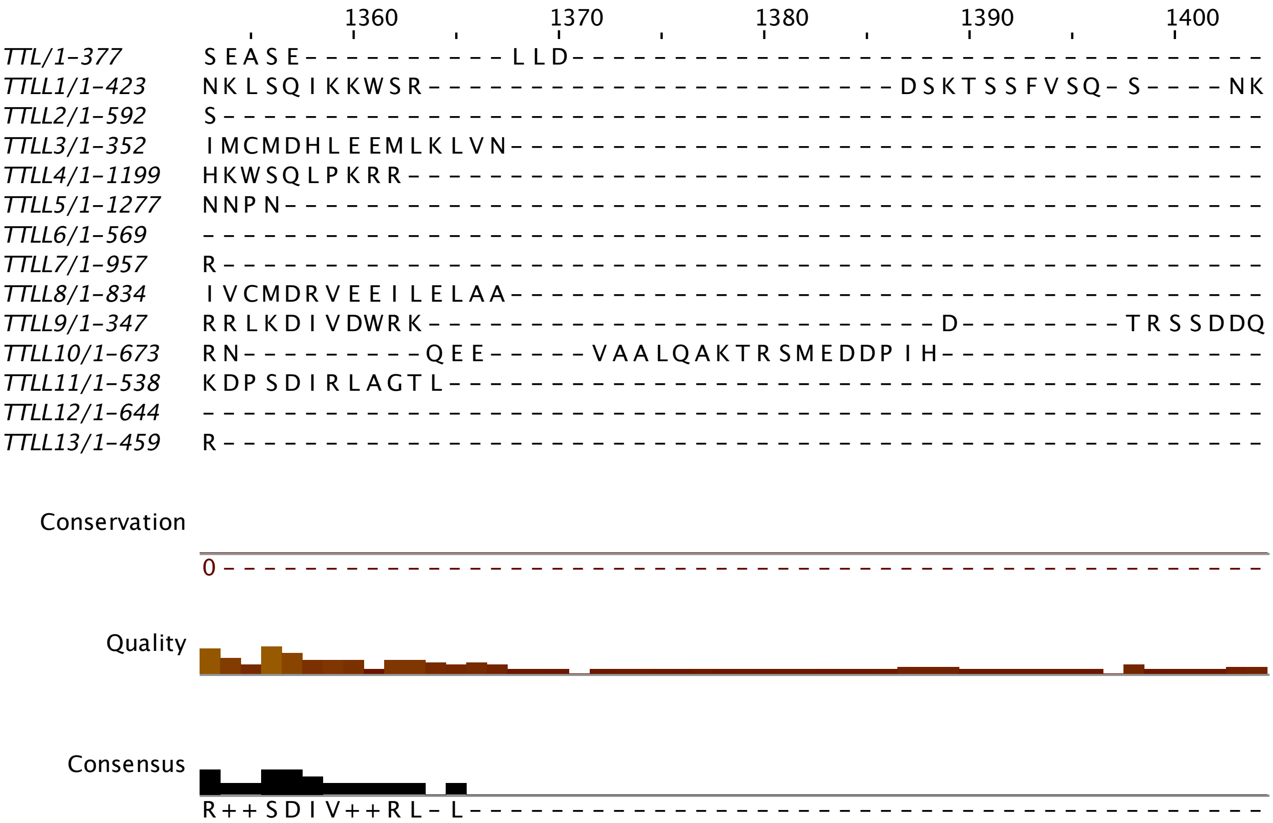
1360 1370 1380 1390 1400
TTL/1-377 SEASE----------LLD-----------------------------------
TTLL1/1-423 NKLSQIKKWSR-----------------------DSKTSSFVSQ-S----NK
TTLL2/1-592 S---
TTLL3/1-352 IMCMDHLEEMLKLVN---------------------------------------
TTLL4/1-1199 HKWSQLPKRR--
TTLL5/1-1277 NNPN--
TTLL6/1-569 --
TTLL7/1-957 R---
TTLL8/1-834 IVCMDRVEEILELAA---------------------------------------
TTLL9/1-347 RRLKDIVDWRK-------------------------D-------TRSSDDQ
TTLL10/1-673 RN---------QEE-----VAALQAKTRSMEDDPIH------------------
TTLL11/1-538 KDPSDIRLAGTL--
TTLL12/1-644 --
TTLL13/1-459 R---
Conservation
0
Quality
Consensus
R++SDIV++RL-L---

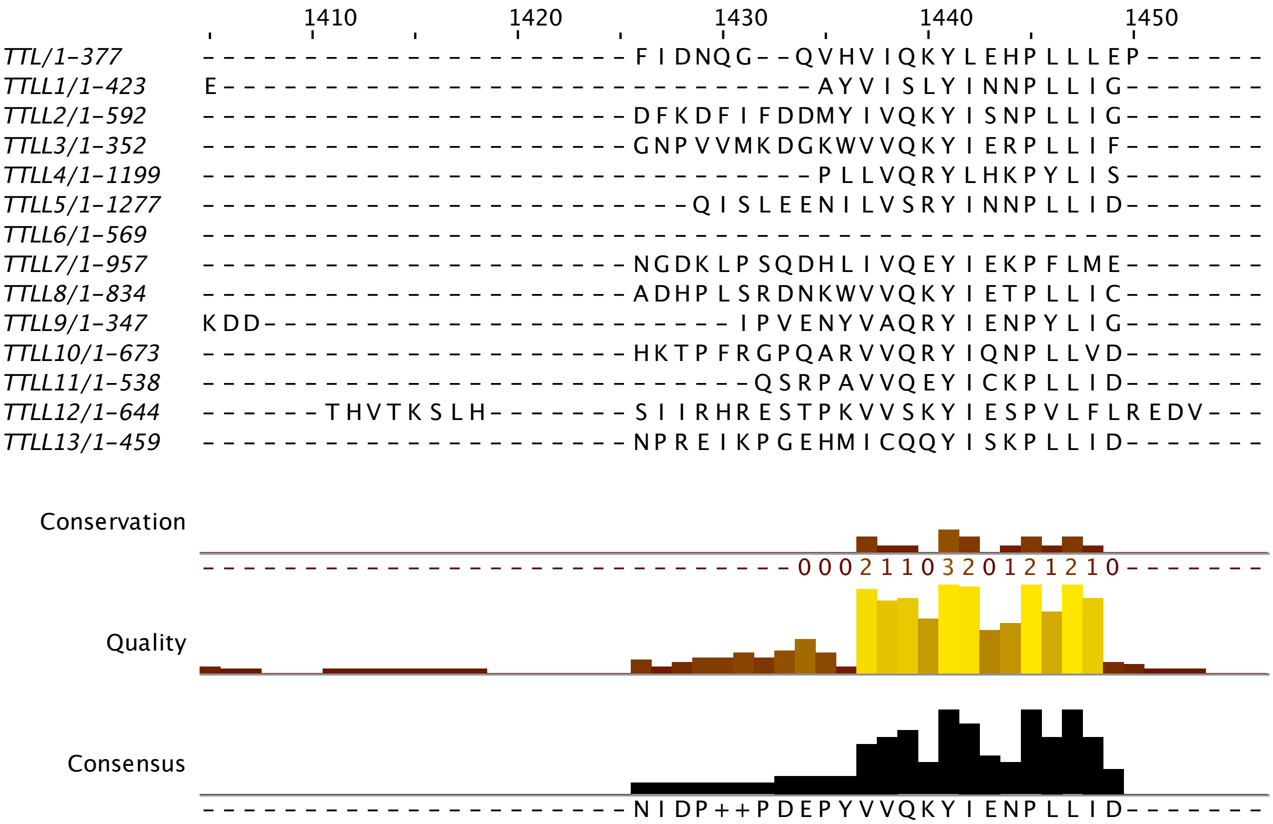
1410 1420 1430 1440 1450
TTL/1-377 ---------------------FIDNQG--QVHVIQKYLEHPLLLEP------
TTLL1/1-423 E------------------------AYVISLYINNPLLIG-------
TTLL2/1-592 ---------------------DFKDFIFDDMYIVQKYISNPLLIG-------
TTLL3/1-352 ---------------------GNPVVMKDGKWVVQKYIERPLLIF-------
TTLL4/1-1199 ------------------------------PLLVQRYLHKPYLIS-------
TTLL5/1-1277 ------------------------QISLEENILVSRYINNPLLID-------
TTLL6/1-569 --
TTLL7/1-957 ---------------------NGDKLPSQDHLIVQEYIEKPFLME-------
TTLL8/1-834 ---------------------ADHPLSRDNKWVVQKYIETPLLIC-------
TTLL9/1-347 KDD-------------------------IPVENYVAQRYIENPYLIG-------
TTLL10/1-673 ---------------------HKTPFRGPQARVVQRYIQNPLLVD-------
TTLL11/1-538 ---------------------------QSRPAVVQEYICKPLLID-------
TTLL12/1-644 ------THVTKSLH-------SIIRHRESTPKVVSKYIESPVLFLREDV---
TTLL13/1-459 ---------------------NPREIKPGEHMICQQYISKPLLID-------
Conservation
-----------------------------0002110320121210-------
Quality
Consensus
---------------------NIDP++PDEPYVVQKYIENPLLID-------

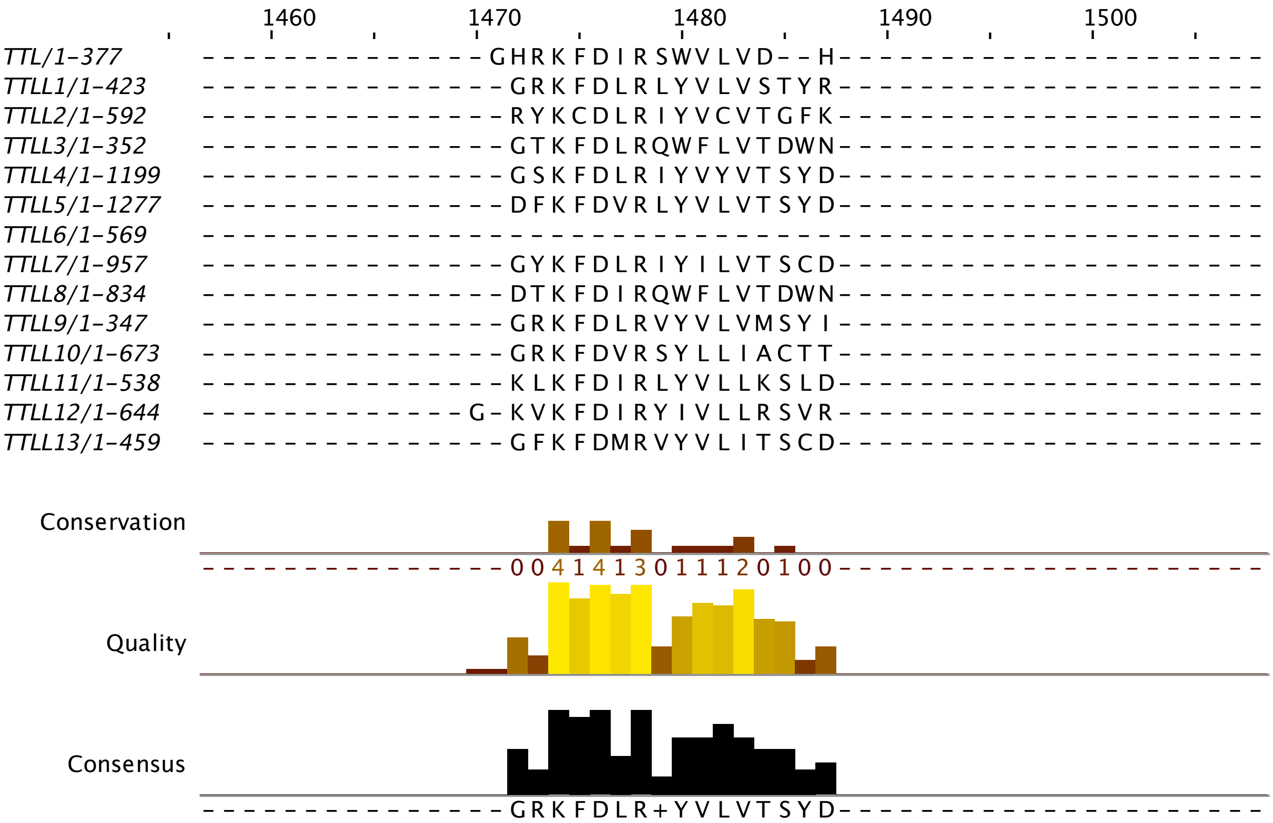
1460
1470
1480
1490
1500
TTL/1-377 --------------GHRKFDIRSWVLVD--H---------------------
TTLL1/1-423 ---------------GRKFDLRLYVLVSTYR---------------------
TTLL2/1-592 ---------------RYKCDLRIYVCVTGFK---------------------
TTLL3/1-352 ---------------GTKFDLRQWFLVTDWN---------------------
TTLL4/1-1199 ---------------GSKFDLRIYVYVTSYD---------------------
TTLL5/1-1277 ---------------DFKFDVRLYVLVTSYD---------------------
TTLL6/1-569 --
TTLL7/1-957 ---------------GYKFDLRIYILVTSCD---------------------
TTLL8/1-834 ---------------DTKFDIRQWFLVTDWN---------------------
TTLL9/1-347 ---------------GRKFDLRVYVLVMSYI---------------------
TTLL10/1-673 ---------------GRKFDVRSYLLIACTT---------------------
TTLL11/1-538 ---------------KLKFDIRLYVLLKSLD---------------------
TTLL12/1-644 -------------G-KVKFDIRYIVLLRSVR---------------------
TTLL13/1-459 ---------------GFKFDMRVYVLITSCD---------------------
Conservation
---------------0041413011120100---------------------
Quality
Consensus
---------------GRKFDLR+YVLVTSYD---------------------

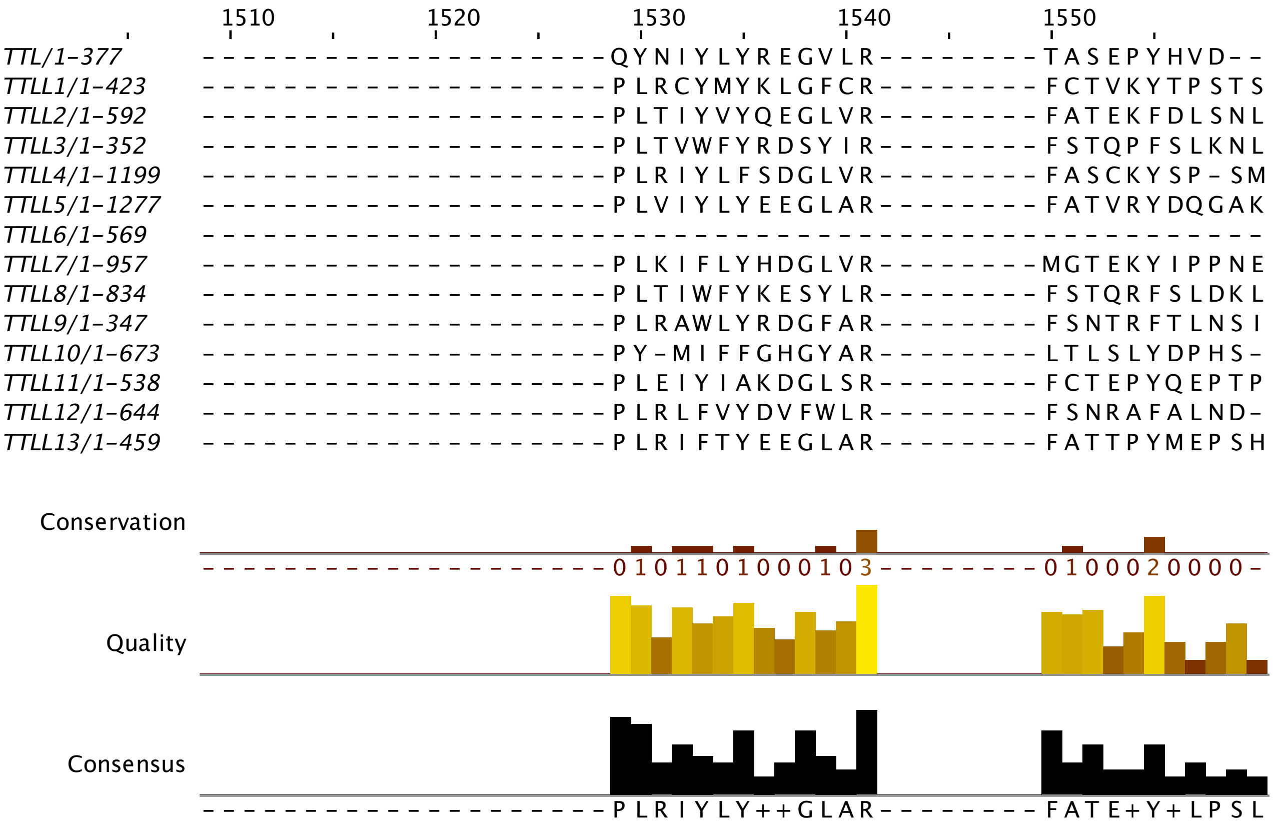
1510
1520
1530
1540
1550
TTL/1-377 QYNIYLYREGVLR TASEPYHVD--
TTLL1/1-423 PLRCYMYKLGFCR FCTVKYTPSTS
TTLL2/1-592 PLTIYVYQEGLVR FATEKFDLSNL
TTLL3/1-352 PLTVWFYRDSYIR FSTQPFSLKNL
TTLL4/1-1199 PLRIYLFSDGLVR FASCKYSP-SM
TTLL5/1-1277 PLVIYLYEEGLAR FATVRYDQGAK
TTLL6/1-569
TTLL7/1-957 PLKIFLYHDGLVR MGTEKYIPPNE
TTLL8/1-834 PLTIWFYKESYLR FSTQRFSLDKL
TTLL9/1-347 PLRAWLYRDGFAR FSNTRFTLNSI
TTLL10/1-673 PY-MIFFGHGYAR LTLSLYDPHS-
TTLL11/1-538 PLEIYIAKDGLSR FCTEPYQEPTP
TTLL12/1-644 PLRLFVYDVFWLR FSNRAFALND-
TTLL13/1-459 PLRIFTYEEGLAR FATTPYMEPSH
Conservation
0101101000103 01000200000
Quality
Consensus
PLRIYLY++GLAR FATE+Y+LPSL

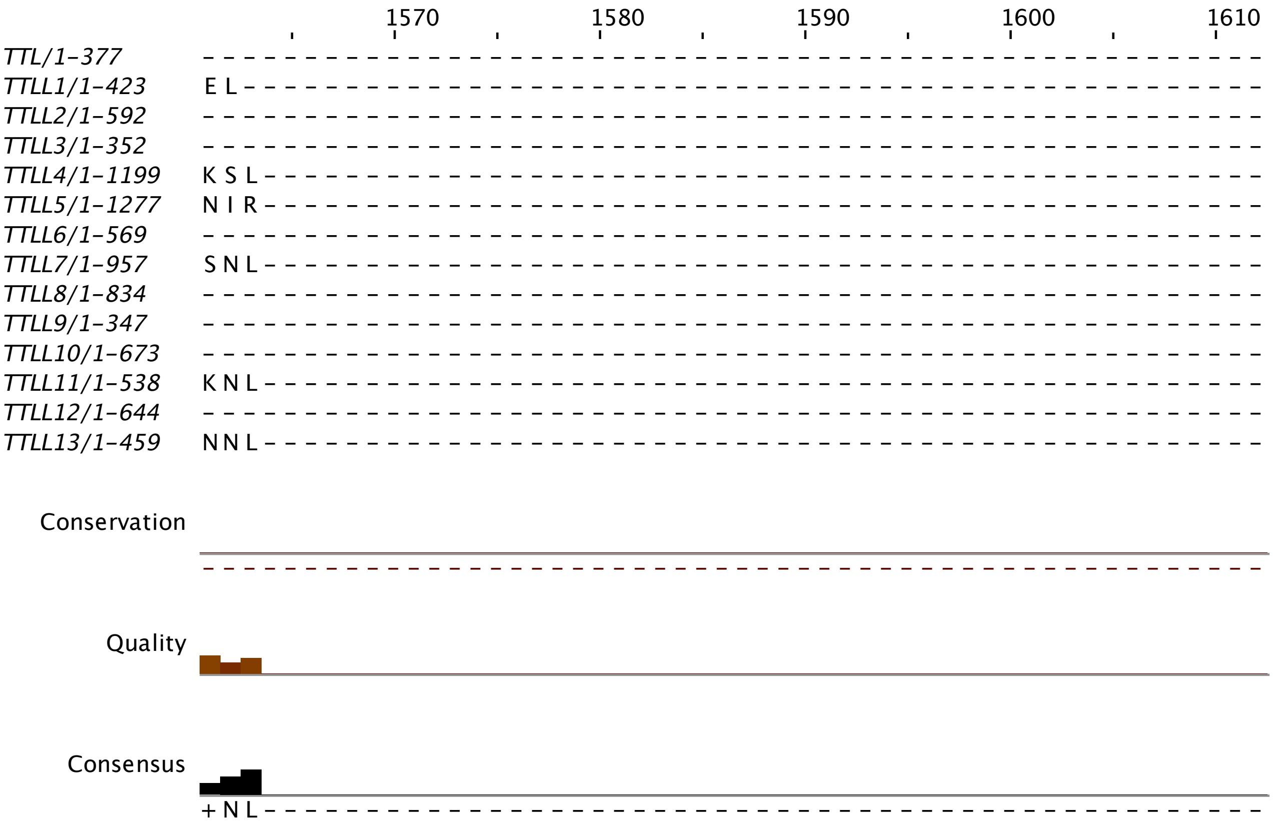
1570
1580
1590
1600
1610
TTL/1-377
TTLL1/1-423 E L
TTLL2/1-592
TTLL3/1-352
TTLL4/1-1199 K S L
TTLL5/1-1277 N I R
TTLL6/1-569
TTLL7/1-957 S N L
TTLL8/1-834
TTLL9/1-347
TTLL10/1-673
TTLL11/1-538 K N L
TTLL12/1-644
TTLL13/1-459 N N L
Conservation
Quality
Consensus
+ N L

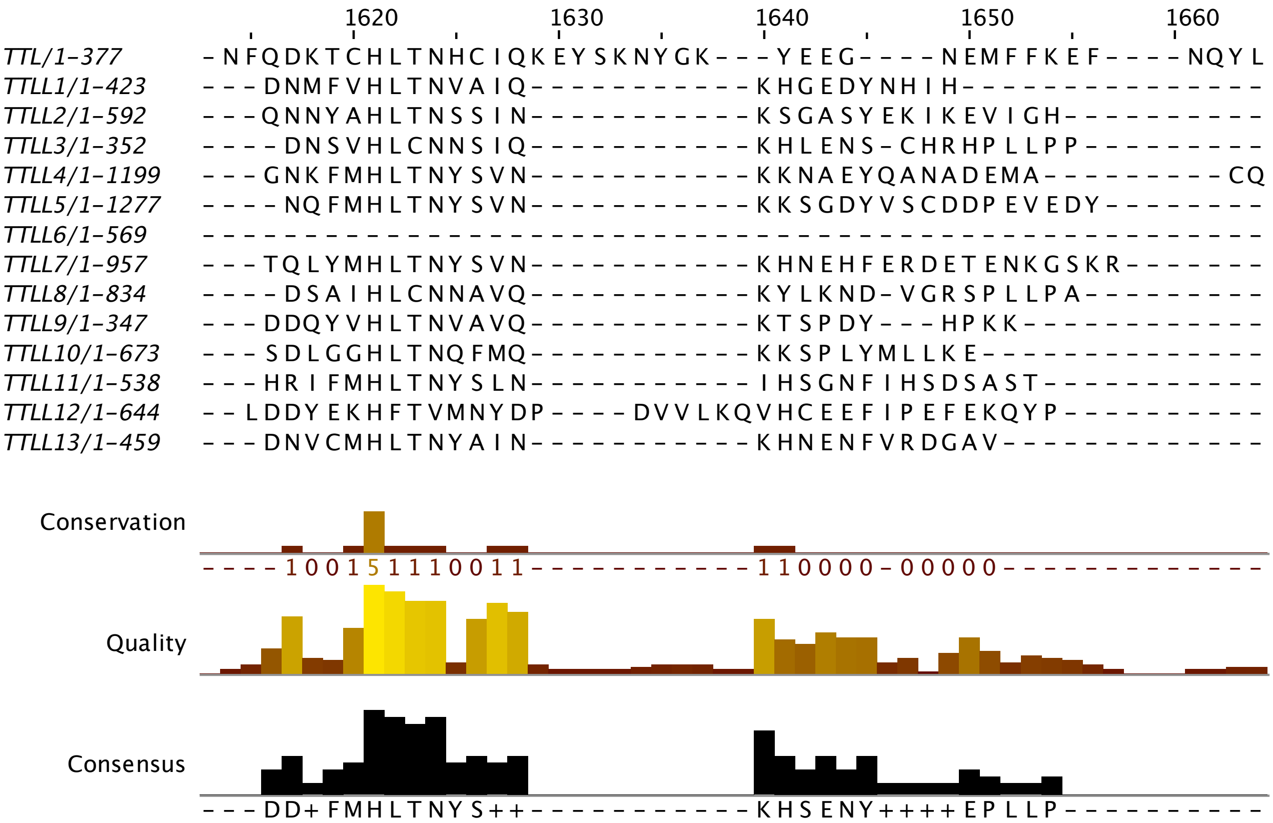

1620 1630 1640 1650 1660
TTL/1-377 -NFQDKTCHLTNHCIQKEYSKNYGK---YEEG----NEMFFKEF----NQYL
TTL1/1-423 ---DNMFVHLTNVAIQ-----------KHGEDYNHIH---------------
TTL2/1-592 ---QNNYAHLTNSSIN-----------KSGASYEKIKEVIGH----------
TTL3/1-352 ----DNSVHLCNNSIQ-----------KHLENS-CHRHPLLPP---------
TTL4/1-1199 ---GNKFMHLTNYSVN-----------KKNAEYQANADEMA---------CQ
TTL5/1-1277 ----NQFMHLTNYSVN-----------KKSGDYVSCDDPEVEDY--------
TTL6/1-569 --
TTL7/1-957 ---TQLYMHLTNYSVN-----------KHNEHFERDETENKGSKR-------
TTL8/1-834 ----DSAIHLCNNAVQ-----------KYLKND-VGRSPLLPA---------
TTL9/1-347 ---DDQYVHLTNVAVQ-----------KTSPDY---HPKK------------
TTL10/1-673 ---SDLGGHLTNQFMQ-----------KKSPLYMLLKE--------------
TTL11/1-538 ---HRIFMHLTNYSLN-----------IHSGNFIHSDSAST-----------
TTL12/1-644 --LDDYEKHFTVMNYDP----DVVLKQVHCEEFIPEFEKQYP----------
TTL13/1-459 ---DNVCMHLTNYAIN-----------KHNENFVRDGAV-------------
Conservation
----100151110011-----------110000-00000-------------
Quality
Consensus
---DD+FMHLTNYS++-----------KHSENY++++EPLLP----------

```
             1670      1680      1690      1700      1710
TTL/1-377    TSA-LNI---------------------------------------------
TTLL1/1-423  ----------------------------------------------------
TTLL2/1-592  ----------------------------------------------------
TTLL3/1-352  ----------------------------------------------------
TTLL4/1-1199 ----------------------------------------------------
TTLL5/1-1277 ----------------------------------------------------
TTLL6/1-569  ----------------------------------------------------
TTLL7/1-957  ----------------------------------------------------
TTLL8/1-834  ----------------------------------------------------
TTLL9/1-347  ----------------------------------------------------
TTLL10/1-673 ----------------------------------------------------
TTLL11/1-538 ----------------------------------------------------
TTLL12/1-644 ----------------------------------------------------
TTLL13/1-459 ----------------------------------------------------

Conservation
             ----------------------------------------------------

Quality

Consensus
             ----------------------------------------------------
```

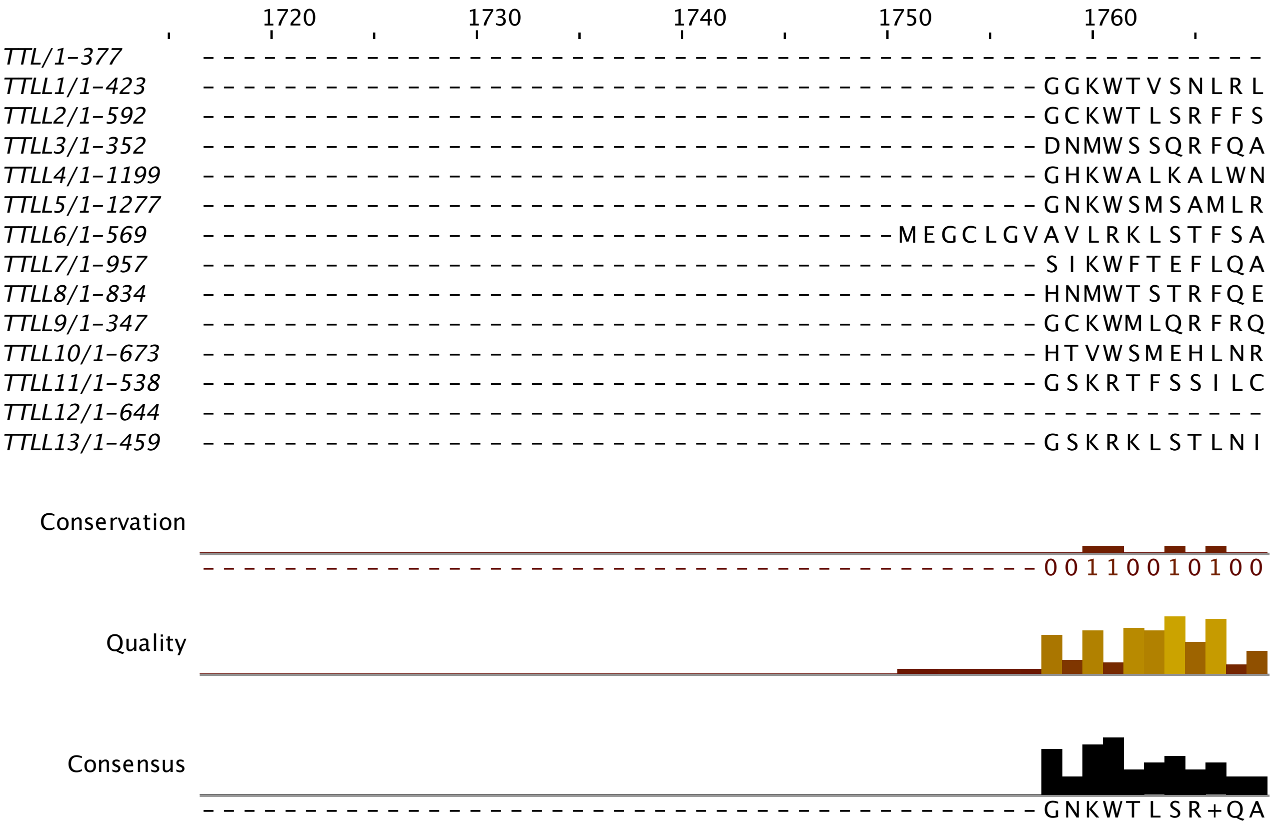
1720 1730 1740 1750 1760
TTL/1-377
TTLL1/1-423 GGKWTVSNLRL
TTLL2/1-592 GCKWTLSRFFS
TTLL3/1-352 DNMWSSQRFQA
TTLL4/1-1199 GHKWALKALWN
TTLL5/1-1277 GNKWSMSAMLR
TTLL6/1-569 MEGCLGVAVLRKLSTFSA
TTLL7/1-957 SIKWFTEFLQA
TTLL8/1-834 HNMWTSTRFQE
TTLL9/1-347 GCKWMLQRFRQ
TTLL10/1-673 HTVWSMEHLNR
TTLL11/1-538 GSKRTFSSILC
TTLL12/1-644
TTLL13/1-459 GSKRKLSTLNI
Conservation
00110010100
Quality
Consensus
GNKWTLSR+QA

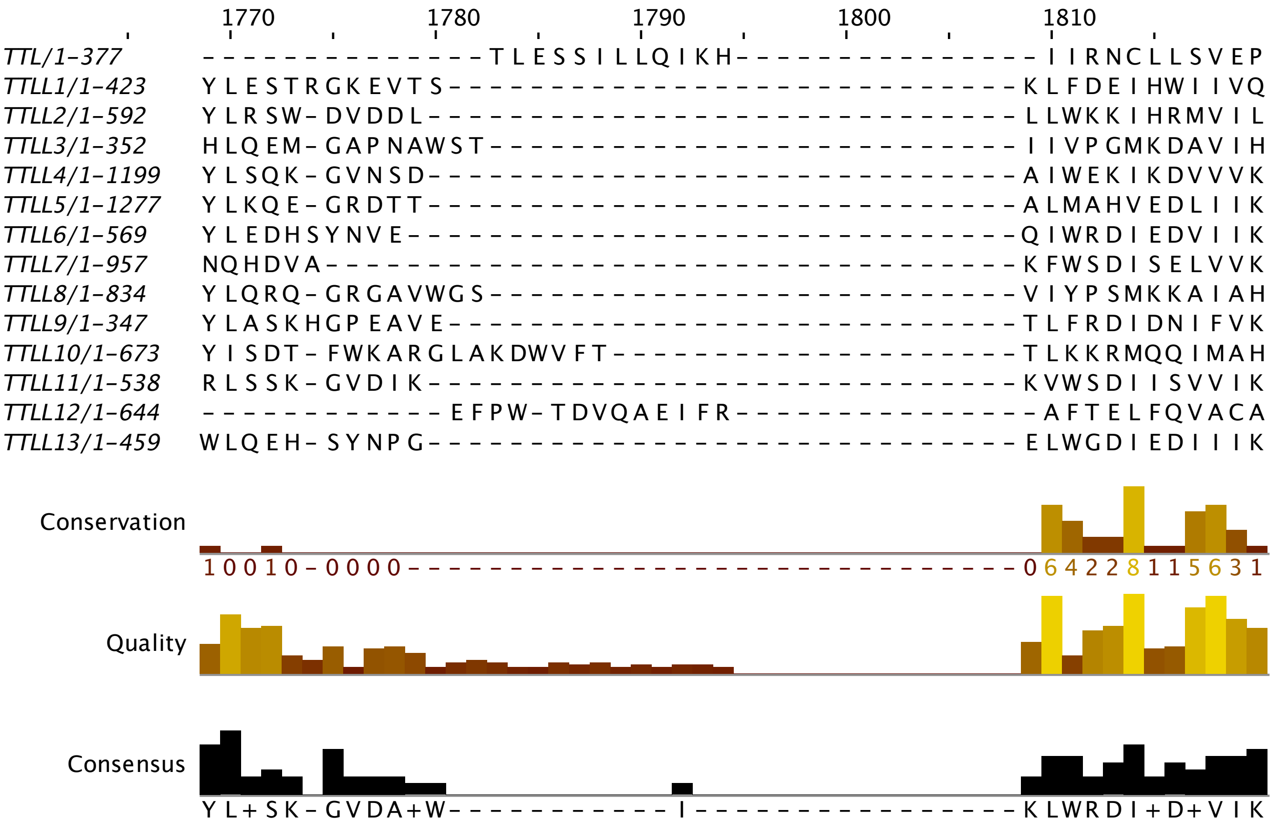
1770
1780
1790
1800
1810
TTL/1-377 --------------TLESSILLQIKH---------------IIRNCLLSVEP
TTLL1/1-423 YLESTRGKEVTS-----------------------------KLFDEIHWIIVQ
TTLL2/1-592 YLRSW-DVDDL------------------------------LLWKKIHRMVIL
TTLL3/1-352 HLQEM-GAPNAWST---------------------------IIVPGMKDAVIH
TTLL4/1-1199 YLSQK-GVNSD------------------------------AIWEKIKDVVVK
TTLL5/1-1277 YLKQE-GRDTT------------------------------ALMAHVEDLIIK
TTLL6/1-569 YLEDHSYNVE-------------------------------QIWRDIEDVIIK
TTLL7/1-957 NQHDVA-----------------------------------KFWSDISELVVK
TTLL8/1-834 YLQRQ-GRGAVWGS---------------------------VIYPSMKKAIAH
TTLL9/1-347 YLASKHGPEAVE-----------------------------TLFRDIDNIFVK
TTLL10/1-673 YISDT-FWKARGLAKDWVFT---------------------TLKKRMQQIMAH
TTLL11/1-538 RLSSK-GVDIK------------------------------KVWSDIISVVIK
TTLL12/1-644 ------------EFPW-TDVQAEIFR---------------AFTELFQVACA
TTLL13/1-459 WLQEH-SYNPG------------------------------ELWGDIEDIIIK
Conservation
10010-0000------------------------------064228115631
Quality
Consensus
YL+SK-GVDA+W----------I---------------KLWRDI+D+VIK

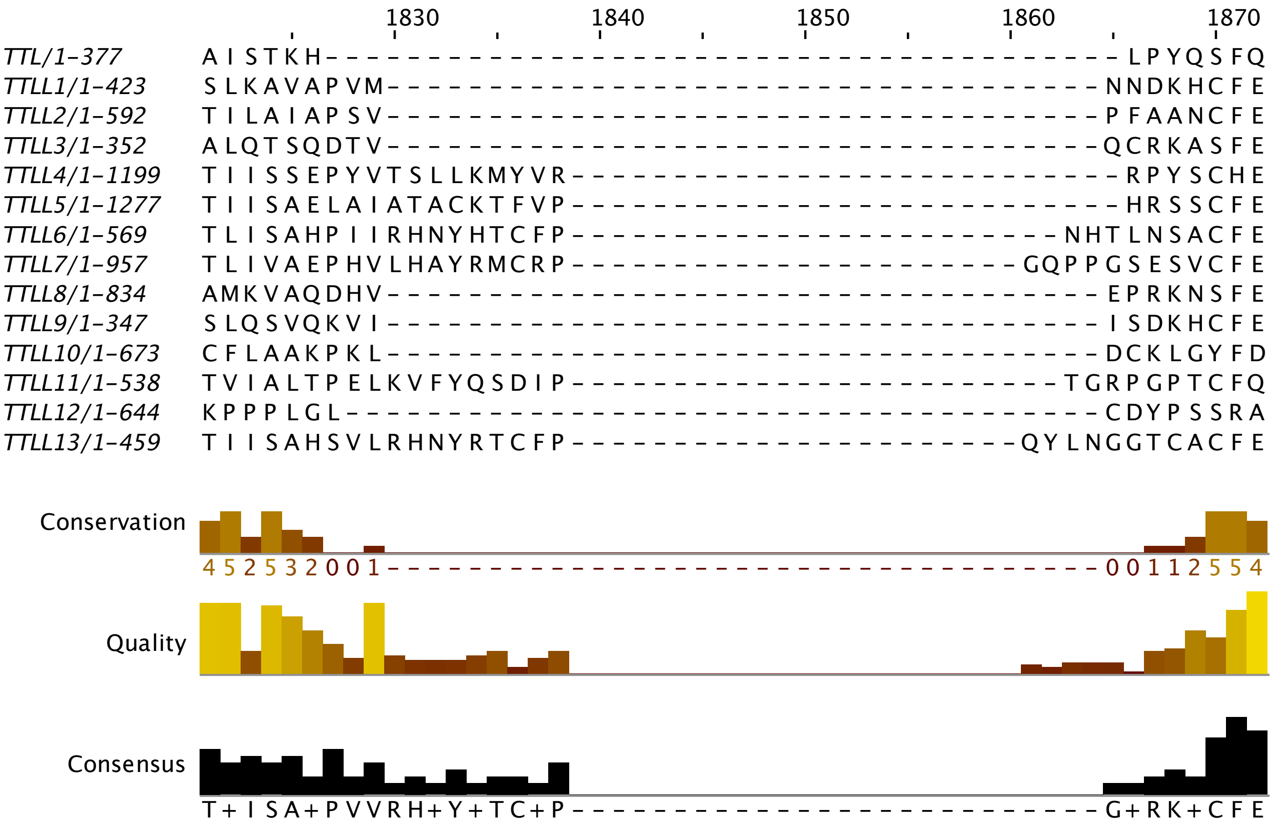
1830 1840 1850 1860 1870
TTL/1-377 AISTKH------------------------------------LPYQSFQ
TTLL1/1-423 SLKAVAPVM----------------------------------NNDKHCFE
TTLL2/1-592 TILAIAPSV----------------------------------PFAANCFE
TTLL3/1-352 ALQTSQDTV----------------------------------QCRKASFE
TTLL4/1-1199 TIISSEPYVTSLLKMYVR--------------------------RPYSCHE
TTLL5/1-1277 TIISAELAIATACKTFVP--------------------------HRSSCFE
TTLL6/1-569 TLISAHPIIRHNYHTCFP------------------------NHTLNSACFE
TTLL7/1-957 TLIVAEPHVLHAYRMCRP----------------------GQPPGSESVCFE
TTLL8/1-834 AMKVAQDHV----------------------------------EPRKNSFE
TTLL9/1-347 SLQSVQKVI----------------------------------ISDKHCFE
TTLL10/1-673 CFLAAKPKL----------------------------------DCKLGYFD
TTLL11/1-538 TVIALTPELKVFYQSDIP------------------------TGRPGPTCFQ
TTLL12/1-644 KPPPLGL------------------------------------CDYPSSRA
TTLL13/1-459 TIISAHSVLRHNYRTCFP----------------------QYLNGGTCACFE
Conservation
4525320 01----------------------------------0011 2554
Quality
Consensus
T+ISA+PVVRH+Y+TC+P--------------------------G+RK+CFE

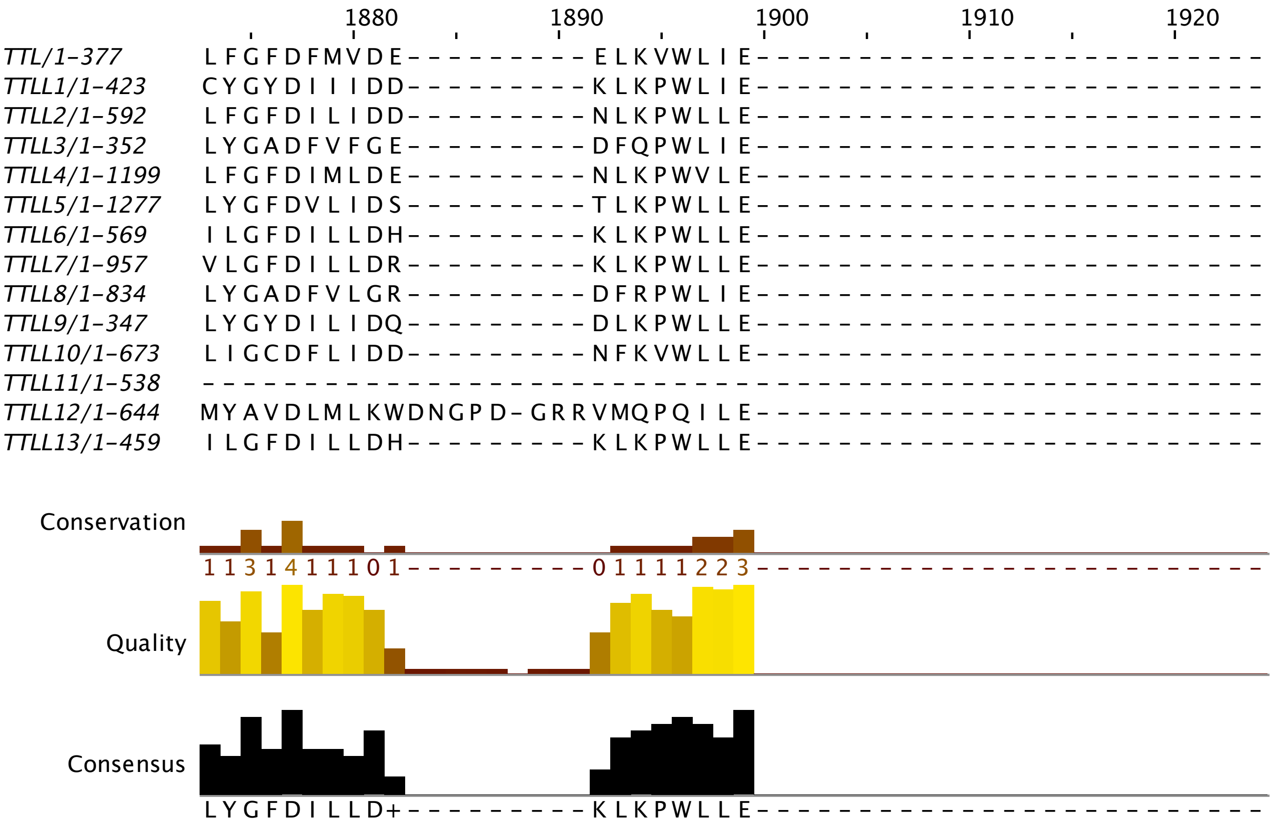
1880 1890 1900 1910 1920
TTL/1-377 LFGFDFMVDE---------ELKVWLIE-----------------------
TTLL1/1-423 CYGYDIIIDD---------KLKPWLIE-----------------------
TTLL2/1-592 LFGFDILIDD---------NLKPWLLE-----------------------
TTLL3/1-352 LYGADFVFGE---------DFQPWLIE-----------------------
TTLL4/1-1199 LFGFDIMLDE---------NLKPWVLE-----------------------
TTLL5/1-1277 LYGFDVLIDS---------TLKPWLLE-----------------------
TTLL6/1-569 ILGFDILLDH---------KLKPWLLE-----------------------
TTLL7/1-957 VLGFDILLDR---------KLKPWLLE-----------------------
TTLL8/1-834 LYGADFVLGR---------DFRPWLIE-----------------------
TTLL9/1-347 LYGYDILIDQ---------DLKPWLLE-----------------------
TTLL10/1-673 LIGCDFLIDD---------NFKVWLLE-----------------------
TTLL11/1-538 ---
TTLL12/1-644 MYAVDLMLKWDNGPD-GRRVMQPQILE-----------------------
TTLL13/1-459 ILGFDILLDH---------KLKPWLLE-----------------------
Conservation
1131411101---------011112223-----------------------
Quality
Consensus
LYGFDILLD+---------KLKPWLLE-----------------------

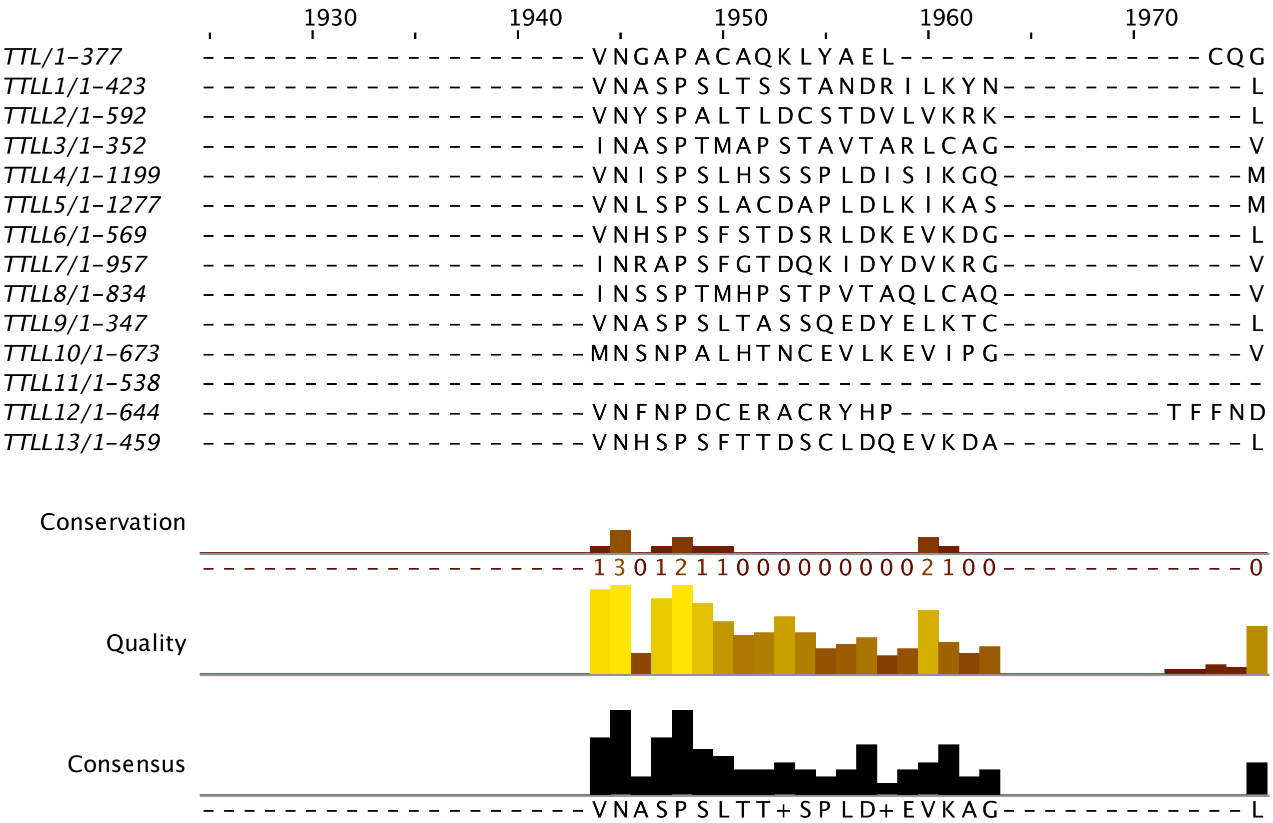
1930
1940
1950
1960
1970
TTL/1-377 VNGAPACAQKLYAEL CQG
TTLL1/1-423 VNASPSLTSSTANDRILKYN L
TTLL2/1-592 VNYSPALTLDCSTDVLVKRK L
TTLL3/1-352 INASPTMAPSTAVTARLCAG V
TTLL4/1-1199 VNISPSLHSSSPLDISIKGQ M
TTLL5/1-1277 VNLSPSLACDAPLDLKIKAS M
TTLL6/1-569 VNHSPSFSTDSRLDKEVKDG L
TTLL7/1-957 INRAPSFGTDQKIDYDVKRG V
TTLL8/1-834 INSSPTMHPSTPVTAQLCAQ V
TTLL9/1-347 VNASPSLTASSQEDYELKTC L
TTLL10/1-673 MNSNPALHTNCEVLKEVIPG V
TTLL11/1-538
TTLL12/1-644 VNFNPDCERACRYHP TFFND
TTLL13/1-459 VNHSPSFTTDSCLDQEVKDA L
Conservation
13012110000000002100 0
Quality
Consensus
VNASPSLTT+SPLD+EVKAG L

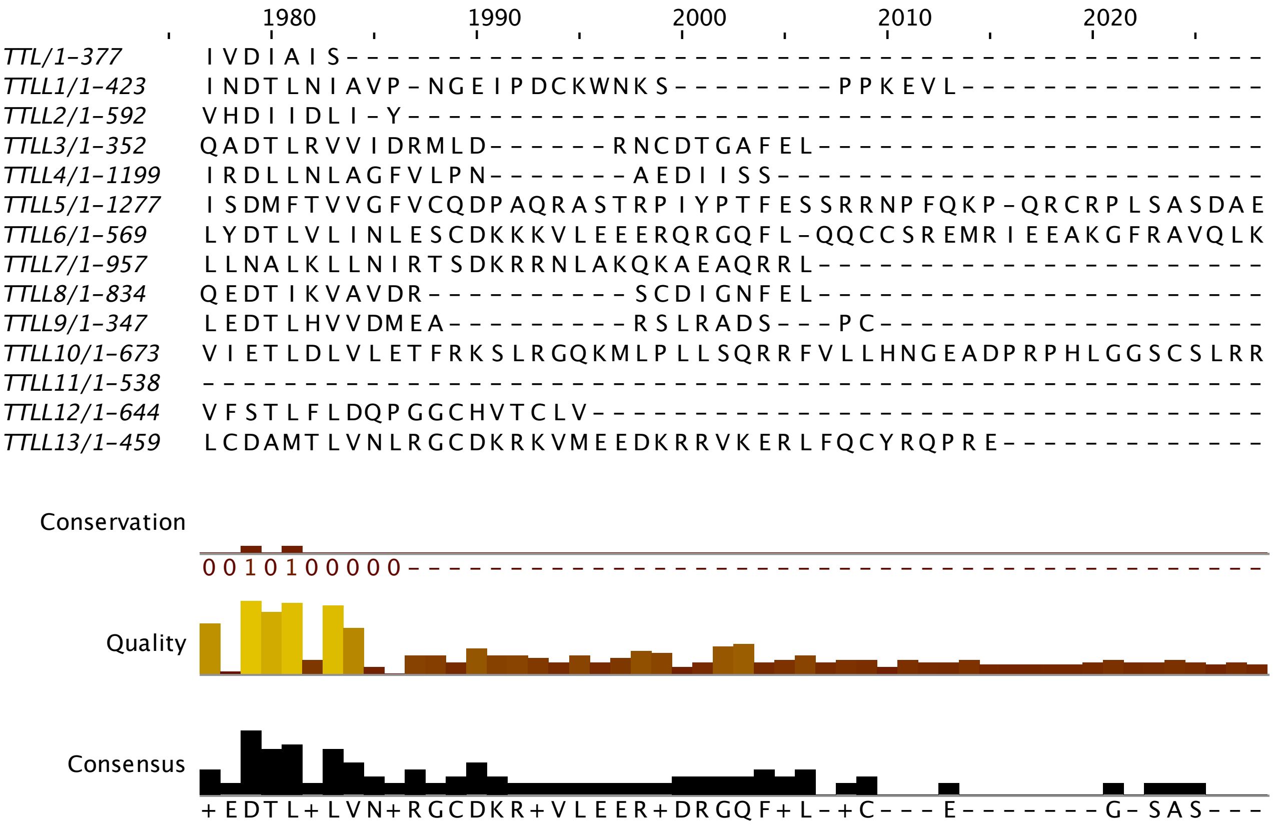
1980 1990 2000 2010 2020
TTL/1-377 IVDIAIS---
TTLL1/1-423 INDTLNIAVP-NGEIPDCKWNKS--------PPKEVL--------------
TTLL2/1-592 VHDIIDLI-Y---------------------------------------
TTLL3/1-352 QADTLRVVIDRMLD------RNCDTGAFEL-----------------------
TTLL4/1-1199 IRDLLNLAGFVLPN-------AEDIISS------------------------
TTLL5/1-1277 ISDMFTVVGFVCQDPAQRASTRPIYPTFESSRRNPFQKP-QRCRPLSASDAE
TTLL6/1-569 LYDTLVLINLESCDKKKVLEEERQRGQFL-QQCCSREMRIEEAKGFRAVQLK
TTLL7/1-957 LLNALKLLNIRTSDKRRNLAKQKAEAQRRL----------------------
TTLL8/1-834 QEDTIKVAVDR----------SCDIGNFEL----------------------
TTLL9/1-347 LEDTLHVVDMEA---------RSLRADS---PC-------------------
TTLL10/1-673 VIETLDLVLETFRKSLRGQKMLPLLSQRRFVLLHNGEADPRPHLGGSCSLRR
TTLL11/1-538 --
TTLL12/1-644 VFSTLFLDQPGGCHVTCLV---------------------------------
TTLL13/1-459 LCDAMTLVNLRGCDKRKVMEEDKRRVKERLFQCYRQPRE-------------
Conservation
0010100000--
Quality
Consensus
+EDTL+LVN+RGCDKR+VLEER+DRGQF+L-+C---E-------G-SAS---

```
                 2030      2040      2050      2060      2070
TTL/1-377      ------------------------------SVFPPPDVE----QPQTQP---
TTLL1/1-423    ----------------------------------------------------
TTLL2/1-592    ----------------------------------------------------
TTLL3/1-352    ----------------------------------------------------
TTLL4/1-1199   ----------------------------------------------------
TTLL5/1-1277   MKNLVGSAREKGPGKLGGSVLGLSMEEIKVLRRVKEENDRRGGFIRIFPTSE
TTLL6/1-569    KTET------------------------------------------------
TTLL7/1-957    ----------------------------------------------------
TTLL8/1-834    ----------------------------------------------------
TTLL9/1-347    ----------------------------------------------------
TTLL10/1-673   WPPLPTRQAKSSGPPM------------------------------------
TTLL11/1-538   ----------------------------------------------------
TTLL12/1-644   ----------------------------------------------------
TTLL13/1-459   ----------------------------------------------------

Conservation
               ----------------------------------------------------

Quality

Consensus
               ---L--------------------------------------------P---
```

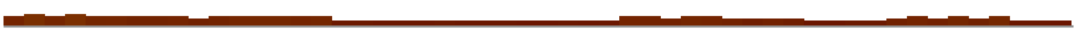

```
              2090      2100      2110      2120      2130
TTL/1-377     ---------AAFIKL-------------------------------------
TTLL1/1-423   ----------------------------------------------------
TTLL2/1-592   ----------------------------------------------------
TTLL3/1-352   ----------------------------------------------------
TTLL4/1-1199  ----------------------------------------------------
TTLL5/1-1277  TWEIYGSYLEHKTSMNYMLATRLFQDR-------------MTADGAPELKIE
TTLL6/1-569   ----------------------------------------------------
TTLL7/1-957   ----------------------------------------------------
TTLL8/1-834   ----------------------------------------------------
TTLL9/1-347   ----------------------------------------------------
TTLL10/1-673  ----------------------------------------------------
TTLL11/1-538  -----VTIASSQPAFP---------ALTGLKRA-LWLR-VG-----------
TTLL12/1-644  ----------------------------------------------------
TTLL13/1-459  ----------------------------------------------------

Conservation
              ----------------------------------------------------

Quality
```

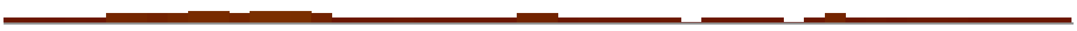

```
Consensus
              ----------------------------------------------------
```

```
                  2140      2150      2160      2170      2180
TTL/1-377     ----------------------------------------------------
TTLL1/1-423   ----------------------------------------------------
TTLL2/1-592   ----------------------------------------------------
TTLL3/1-352   ----------------------------------------------------
TTLL4/1-1199  ----------------------------------------------------
TTLL5/1-1277  SLNSKAKLHAALYERKLLSLEVRKRRRRSSRLRAMRPKYPVITQPAEMNVK-
TTLL6/1-569   ----------------------------------------------------
TTLL7/1-957   ----------------------------------------------------
TTLL8/1-834   ----------------------------------------------------
TTLL9/1-347   ----------------------------------------------------
TTLL10/1-673  ----------------------------------------------------
TTLL11/1-538  ----------------------------------------------------
TTLL12/1-644  ----------------------------------------------------
TTLL13/1-459  ----------------------------------------------------

Conservation
              ----------------------------------------------------

Quality

Consensus
              ----------------------------------------------------
```

```
                 2190      2200      2210      2220      2230
TTL/1-377      ----------------------------------------------------
TTLL1/1-423    ----------------------------------------------------
TTLL2/1-592    ----------------------------------------------------
TTLL3/1-352    ----------------------------------------------------
TTLL4/1-1199   ----------------------------------------------------
TTLL5/1-1277   -------------------TETESEEEEEVALDNEDEEQEASQEESAGFLRE
TTLL6/1-569    ----------------------------------------------------
TTLL7/1-957    ----------------------------------------------------
TTLL8/1-834    ----------------------------------------------------
TTLL9/1-347    ----------------------------------------------------
TTLL10/1-673   P--HAPD---------------------------------------------
TTLL11/1-538   ----------------------------------------------------
TTLL12/1-644   ----------------------------------------------------
TTLL13/1-459   ----------------------------------------------------

Conservation
               ----------------------------------------------------

Quality

Consensus
               ----------------------------------------------------
```

```
                 2240      2250      2260      2270      2280
TTL/1-377       ----------------------------------------------------
TTLL1/1-423     GNYEILYDEELAQGDGADRELRSRQGQS---------------LGPRA----
TTLL2/1-592     --------------------LNGLRNEG-----------GEASNATH-----
TTLL3/1-352     IYKQPVTTSP--------------------------ASTPRPSCLLPMYSDT
TTLL4/1-1199    -PSSCSSSTTSLPTSPGDKCRMAPEHVT---------------AQKMK----
TTLL5/1-1277    NQAKYTPSLTALVENTPKENSMKVREWNNKGGHCCKLETQELEPKFNLMQIL
TTLL6/1-569     ----------------------------------------------------
TTLL7/1-957     YGQNSIKRLLPGSSDWEQQRHQLERRKE---------------ELKER----
TTLL8/1-834     LWRQPVVEPPPFSGSDLCVAG----------------VSVRRARRQVLPVCN
TTLL9/1-347     ------W---------------------------------------------
TTLL10/1-673    QPGARRPAPPPLVPQRPRPPGPDLDSAHDGEPQAPGTEQSGT--GNRH-PAQ
TTLL11/1-538    ----------------------------------------------------
TTLL12/1-644    ----------------------------------------------------
TTLL13/1-459    -SRC----ARCLACV-------------------------------------
```

Conservation

Quality

Consensus

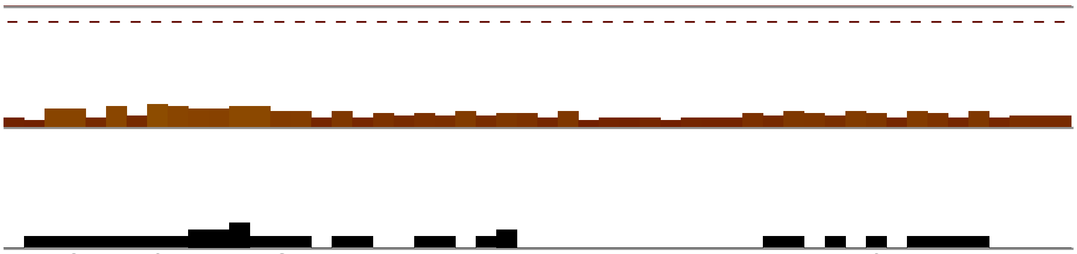

```
                -PRQPVPS+PPL+G+-PD--+M-LR------------ET-+-S-+KRL----
```

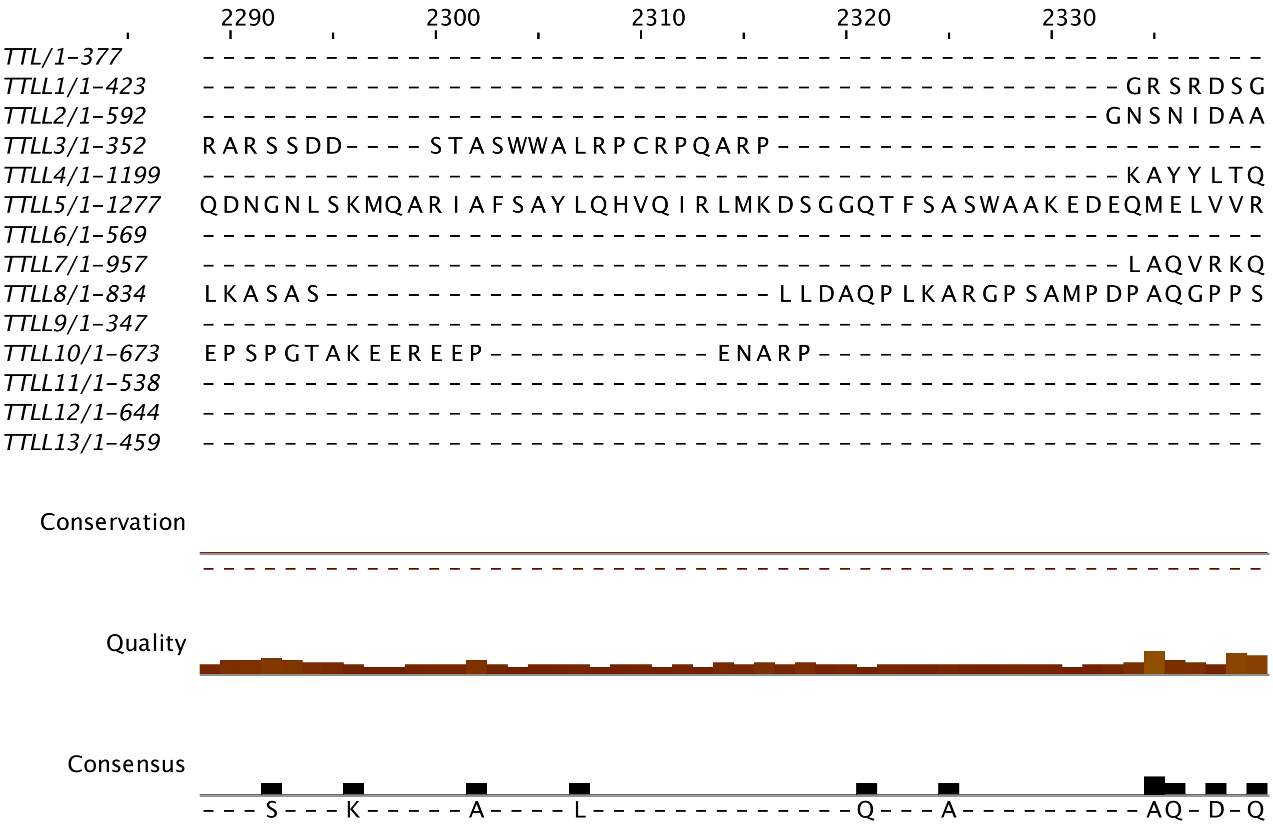
2290
2300
2310
2320
2330
TTL/1-377 ---
TTLL1/1-423 --GRSRDSG
TTLL2/1-592 ---GNSNIDAA
TTLL3/1-352 RARSSDD----STASWWALRPCRPQARP-------------------------
TTLL4/1-1199 --KAYYLTQ
TTLL5/1-1277 QDNGNLSKMQARIAFSAYLQHVQIRLMKDSGGQTFSASWAAKEDEQMELVVR
TTLL6/1-569 ---
TTLL7/1-957 --LAQVRKQ
TTLL8/1-834 LKASAS----------------------LLDAQPLKARGPSAMPDPAQGPPS
TTLL9/1-347 ---
TTLL10/1-673 EPSPGTAKEEREEP-----------ENARP-----------------------
TTLL11/1-538 ---
TTLL12/1-644 ---
TTLL13/1-459 ---
Conservation
Quality
Consensus
---S---K-----A----L-------------Q---A---------AQ-D-Q

```
                   2350      2360      2370      2380      2390
TTL/1-377      ----------------------------------------------------
TTLL1/1-423    RAVLTTWK--------------------------------------------
TTLL2/1-592    K-----SDRGGLDAPDCLPYD----SLSFTSRMYNEDDS-------------
TTLL3/1-352    ----------------------------------------------------
TTLL4/1-1199   KIPDQ--------DFYAS----------------------------------
TTLL5/1-1277   FLKRASNNLQHSLRMVLPSRRLALLERRRILAHQLGDFIIVYNKETEQMAEK
TTLL6/1-569    ---YEKENCGGFRLIYPS--------LNSE--KYEKFFQDNNSLFQNTVASR
TTLL7/1-957    ISREEHENRHMGNYRRIYPPEDKALLEKYENLLAVAFQTFLSGRAASFQREL
TTLL8/1-834    PALQRDLGLKEEKGLPLALLAPLRGAAESGGAAQPTRTKAAGKVELPACPCR
TTLL9/1-347    ----------------------------------------------------
TTLL10/1-673   ----------------------------------------------------
TTLL11/1-538   ----------------------------------------------------
TTLL12/1-644   ----------------------------------------------------
TTLL13/1-459   ----------------------------------------------------

Conservation
               ----------------------------------------------------

Quality

Consensus
               KA--E-EN+GG----YLSP-----L-L-SE-A-+--+F------E----AER
```

```
                 2400      2410      2420      2430      2440
TTL/1-377      ----------------------------------------------------
TTLL1/1-423    ----------------------------------------------------
TTLL2/1-592    ----------------------------------------------------
TTLL3/1-352    ----------------------------------------------------
TTLL4/1-1199   ----------------------------------------------------
TTLL5/1-1277   KSKKKVEEEEEDGVNMENFQEFIRQASEAELEEVLTFYTQKNKSASVFLGTH
TTLL6/1-569    AREEYARQ-LIQELRLKREKKPFQMKKKVEM--QGESAGEQVRKKGMRGWQQ
TTLL7/1-957    NNPLK-RMKEEDILDLLEQCEIDDEKLMGKTTKTR-----------------
TTLL8/1-834    HVDSQAPNTGVPVAQPAKSWDPNQLNAHPLEPVLRGLKTAEGALRPPPGGKG
TTLL9/1-347    ----------------------------------------------------
TTLL10/1-673   ----------------------------------------------------
TTLL11/1-538   ----------------------------------------------------
TTLL12/1-644   ----------------------------------------------------
TTLL13/1-459   ----------------------------------------------------

Conservation
               ----------------------------------------------------
```

Quality

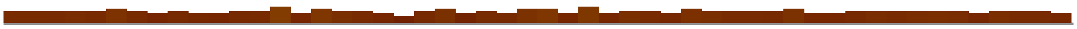

Consensus

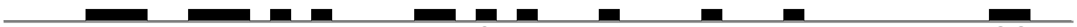

```
               ----KAR--EED-L-L----EP-Q-K---E----R---T---------GG--
```

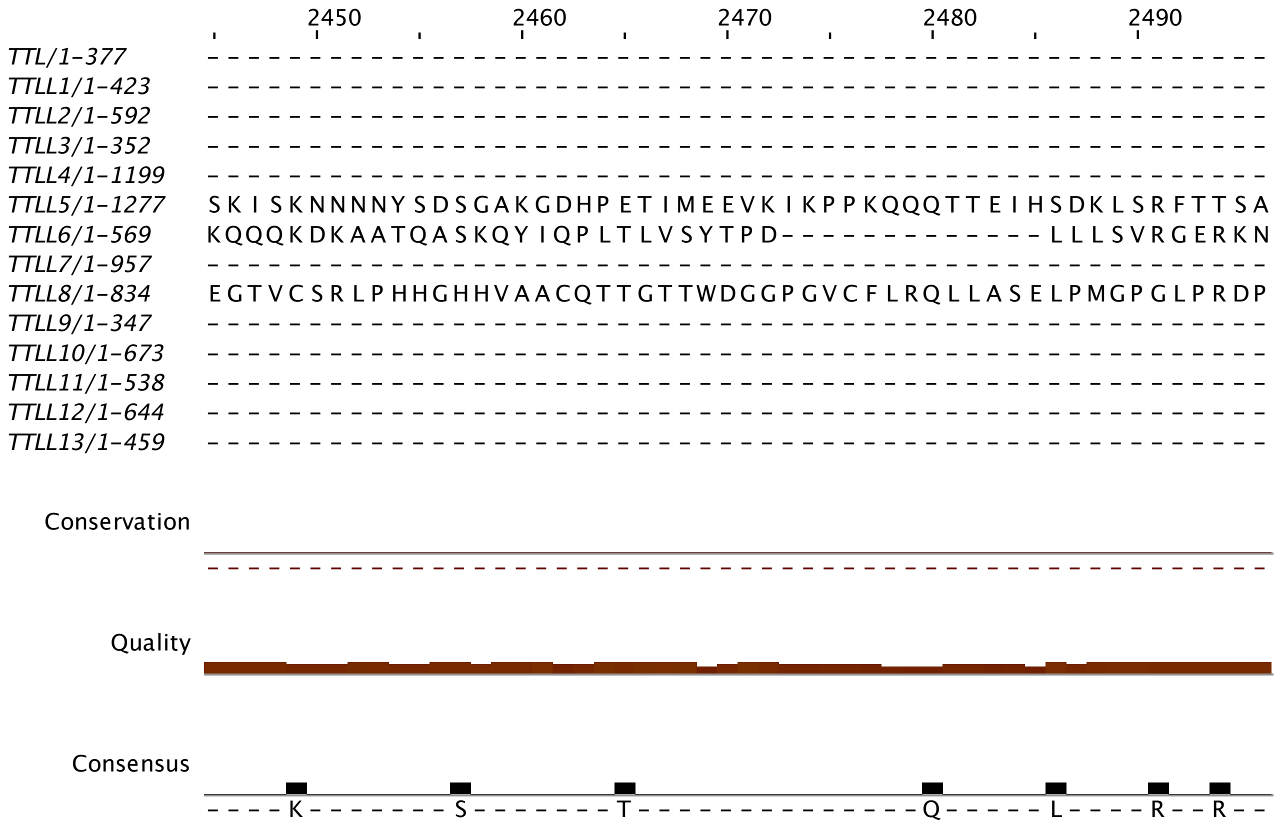

2450
2460
2470
2480
2490
TTL/1-377 --
TTLL1/1-423 --
TTLL2/1-592 --
TTLL3/1-352 --
TTLL4/1-1199 --
TTLL5/1-1277 SKISKNNNNYSDSGAKGDHPETIMEEVKIKPPKQQQTTEIHSDKLSRFTTSA
TTLL6/1-569 KQQQKDKAATQASKQYIQPLTLVSYTPD-------------LLLSVRGERKN
TTLL7/1-957 --
TTLL8/1-834 EGTVCSRLPHHGHHVAACQTTGTTWDGGPGVCFLRQLLASELPMGPGLPRDP
TTLL9/1-347 --
TTLL10/1-673 --
TTLL11/1-538 --
TTLL12/1-644 --
TTLL13/1-459 --
Conservation
Quality
Consensus
----K-------S-------T--------------Q-----L----R--R--

```
                 2500      2510      2520      2530      2540
TTL/1-377       ----------------------------------------------------
TTLL1/1-423     ----------------------------------------------------
TTLL2/1-592     ----------------------------------------------------
TTLL3/1-352     ----------------------------------------------------
TTLL4/1-1199    ----------------------------------------------------
TTLL5/1-1277    EKEAKLVYSNSSSGPTATLQKIPNTHLSSVTTSDLSPGPCHHSSLSQIPSAI
TTLL6/1-569     ETDSSLNQEAPTEEASSVFPKLTSAKPFSSLPDLRNINLSSSKLEPSKPNFS
TTLL7/1-957     ----------------------------------------------------
TTLL8/1-834     RAPPCLVCRGLLPPAGPCKRCRSFCAAVLQGASFVRLGGRSCSPRTP-----
TTLL9/1-347     ----------------------------------------------------
TTLL10/1-673    ----------------------------------------------------
TTLL11/1-538    ----------------------------------------------------
TTLL12/1-644    ----------------------------------------------------
TTLL13/1-459    ----------------------------------------------------

Conservation
                ----------------------------------------------------

Quality

Consensus
                E----LV-------A-----K-------S---S----G--S-S-----P---
```

```
               2550      2560      2570      2580      2590
TTL/1-377      ----------------------------------------------------
TTLL1/1-423    ----------------------------------------------------
TTLL2/1-592    ----------------------------------------------------
TTLL3/1-352    ----------------------------------------------------
TTLL4/1-1199   ----------------------------------------------------
TTLL5/1-1277   PSMPHQPTILLNTVSASASPCLHPGAQNIPSPTGLPRCR----SGSHTIGPF
TTLL6/1-569    IKEAKSASAVNVFTGTVHLTSVETTPESTTQLSISPKSPPTLAVTASSEYSG
TTLL7/1-957    ----------------------------------------------------
TTLL8/1-834    ----------------------------------------------------
TTLL9/1-347    ----------------------------------------------------
TTLL10/1-673   ----------------------------------------------------
TTLL11/1-538   ----------------------------------------------------
TTLL12/1-644   ----------------------------------------------------
TTLL13/1-459   ----------------------------------------------------

Conservation
               ----------------------------------------------------

Quality

Consensus
               -----------------------------------P----------------
```

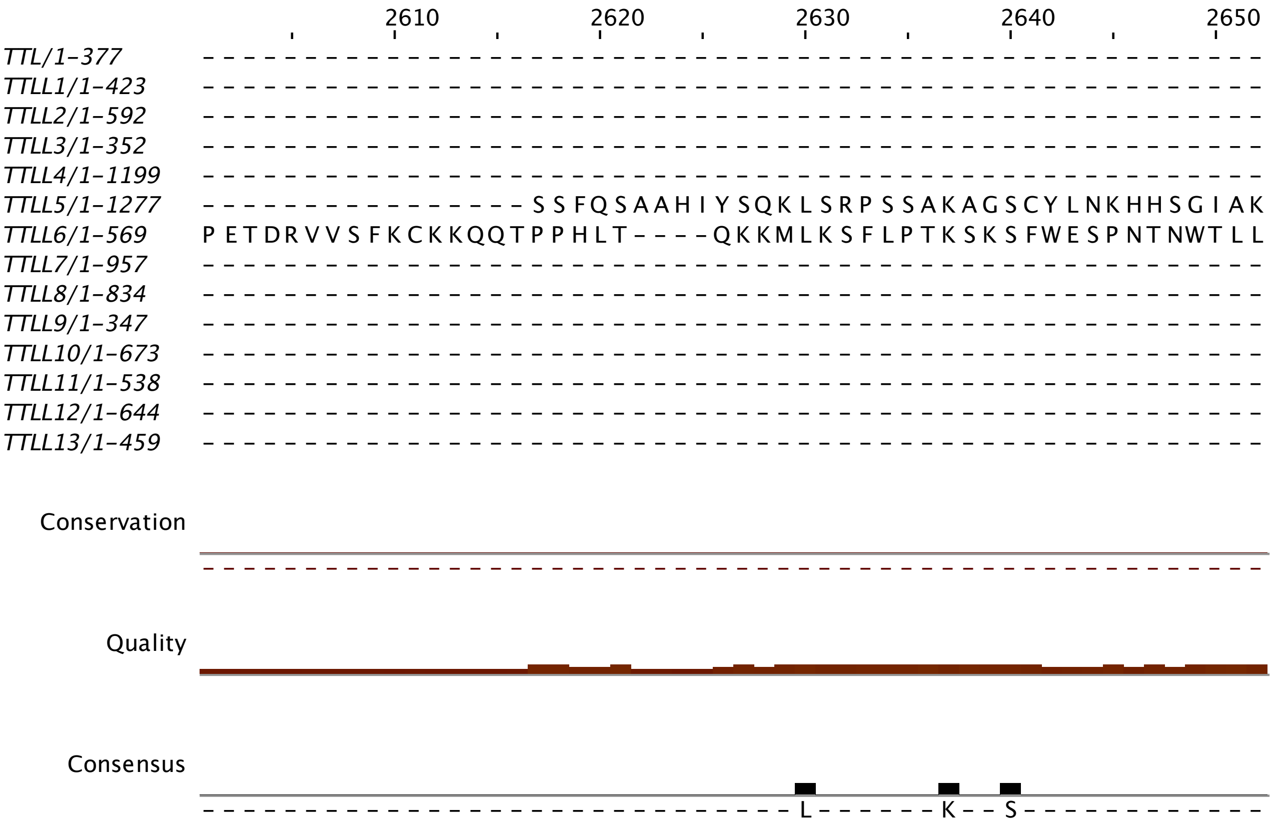
2610
2620
2630
2640
2650
TTL/1-377
TTLL1/1-423
TTLL2/1-592
TTLL3/1-352
TTLL4/1-1199
TTLL5/1-1277 ----------------SSFQSAAHIYSQKLSRPSSAKAGSCYLNKHHSGIAK
TTLL6/1-569 PETDRVVSFKCKKQQTPPHLT----QKKMLKSFLPTKSKSFWESPNTNWTLL
TTLL7/1-957
TTLL8/1-834
TTLL9/1-347
TTLL10/1-673
TTLL11/1-538
TTLL12/1-644
TTLL13/1-459
Conservation
Quality
Consensus
L
K
S

```
                 2660      2670      2680      2690      2700
TTL/1-377      ----------------------------------------------------
TTLL1/1-423    ----------------------------------------------------
TTLL2/1-592    ----------------------------------------------------
TTLL3/1-352    ----------------------------------------------------
TTLL4/1-1199   ----------------------------------------------------
TTLL5/1-1277   TQKEGEDASLYSK----RYNQSMVTAELQRLAEKQAARQYSPSSHINLLTQQ
TTLL6/1-569    KSDMNKPHLISELLTKLQLSGKLSFFPAHYNPKLGMNNLSQNPSLPGECHSR
TTLL7/1-957    ----------------------------------------------------
TTLL8/1-834    ----------------------------------------------------
TTLL9/1-347    ----------------------------------------------------
TTLL10/1-673   ----------------------------------------------------
TTLL11/1-538   ----------------------------------------------------
TTLL12/1-644   ----------------------------------------------------
TTLL13/1-459   ----------------------------------------------------

Conservation
               ----------------------------------------------------

Quality
```



```
Consensus
               -------------------------------------------S--------
```

```
                     2710      2720      2730      2740      2750
                ....'....|....'....|....'....|....'....|....'....|
TTL/1-377       ----------------------------------------------------
TTLL1/1-423     ----------------------------------------------------
TTLL2/1-592     ----------------------------------------------------
TTLL3/1-352     ----------------------------------------------------
TTLL4/1-1199    ----------------------------------------------------
TTLL5/1-1277    VTNLNLATGIIN------------------------RSSASAPPTLRPIISP
TTLL6/1-569     SDSSGEKRQLDVSSLLL-----------------------------------
TTLL7/1-957     ----------------------------------------------------
TTLL8/1-834     ----------------------------------------------------
TTLL9/1-347     ----------------------------------------------------
TTLL10/1-673    ----------------------------------------------------
TTLL11/1-538    ----------------------------------------------------
TTLL12/1-644    ----------------------------------------------------
TTLL13/1-459    ----------------------------------------------------

Conservation
                ----------------------------------------------------

Quality

Consensus
                ----------------------------------------------------
```

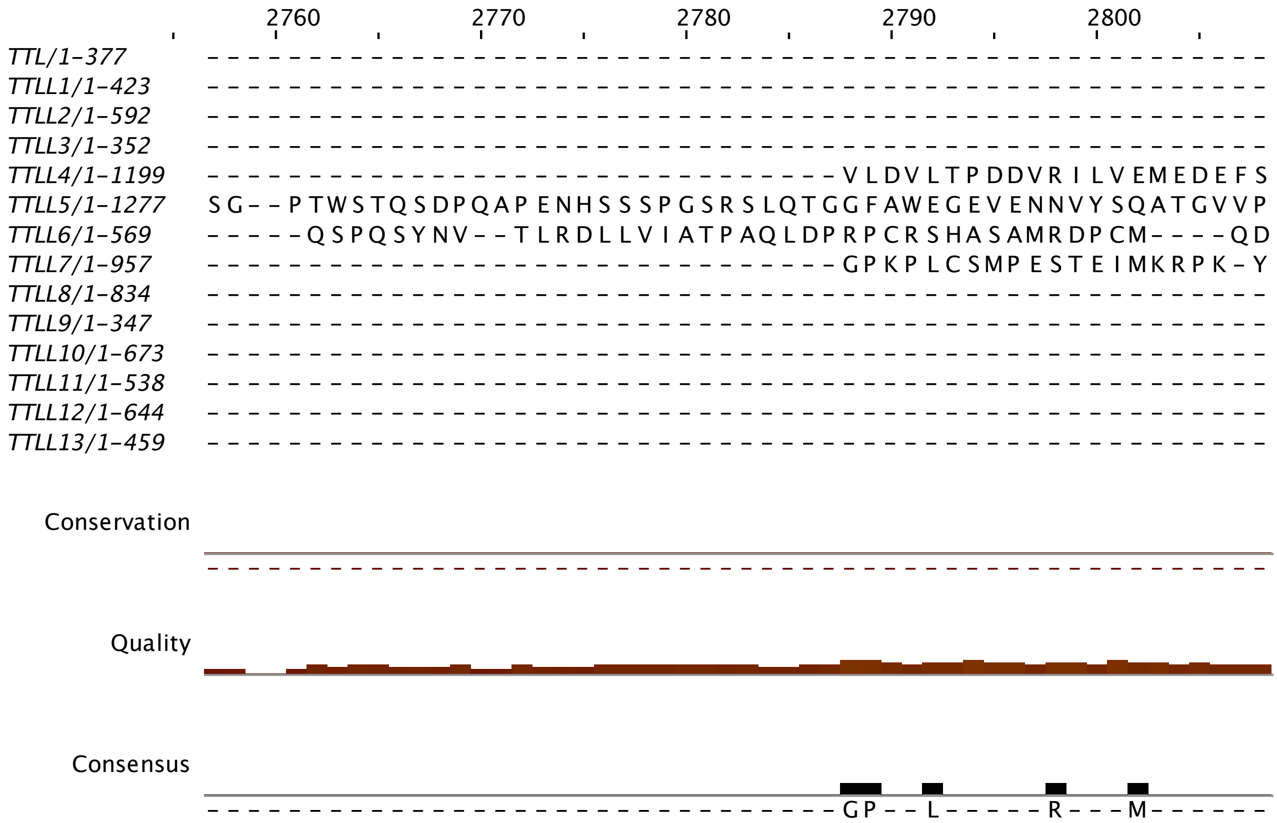
2760
2770
2780
2790
2800
TTL/1-377
TTLL1/1-423
TTLL2/1-592
TTLL3/1-352
TTLL4/1-1199 VLDVLTPDDVRILVEMEDEFS
TTLL5/1-1277 SG--PTWSTQSDPQAPENHSSSPGSRSLQTGGFAWEGEVENNVYSQATGVVP
TTLL6/1-569 -----QSPQSYNV--TLRDLLVIATPAQLDPRPCRSHASAMRDPCM----QD
TTLL7/1-957 GPKPLCSMPESTEIMKRPK-Y
TTLL8/1-834
TTLL9/1-347
TTLL10/1-673
TTLL11/1-538
TTLL12/1-644
TTLL13/1-459
Conservation
Quality
Consensus
GP--L-----R---M------

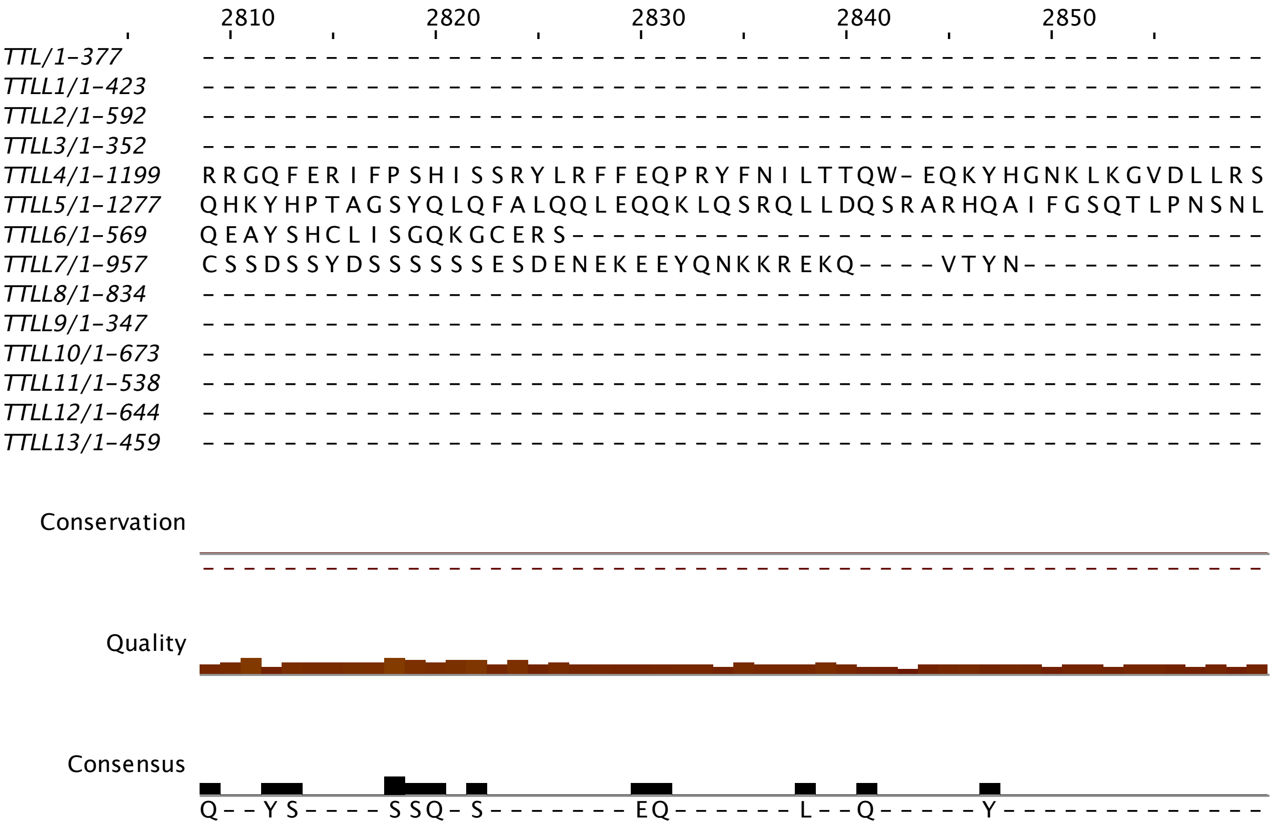
2810 2820 2830 2840 2850
TTL/1-377 --
TTLL1/1-423 --
TTLL2/1-592 --
TTLL3/1-352 --
TTLL4/1-1199 RRGQFERIFPSHISSRYLRFFEQPRYFNILTTQW-EQKYHGNKLKGVDLLRS
TTLL5/1-1277 QHKYHPTAGSYQLQFALQQLEQQKLQSRQLLDQSRARHQAIFGSQTLPNSNL
TTLL6/1-569 QEAYSHCLISGQKGCERS-------------------------------------
TTLL7/1-957 CSSDSSYDSSSSSSESDENEKEEYQNKKREKQ----VTYN------------
TTLL8/1-834 --
TTLL9/1-347 --
TTLL10/1-673 --
TTLL11/1-538 --
TTLL12/1-644 --
TTLL13/1-459 --
Conservation
Quality
Consensus
Q--YS----SSQ-S--------EQ------L--Q-----Y------------

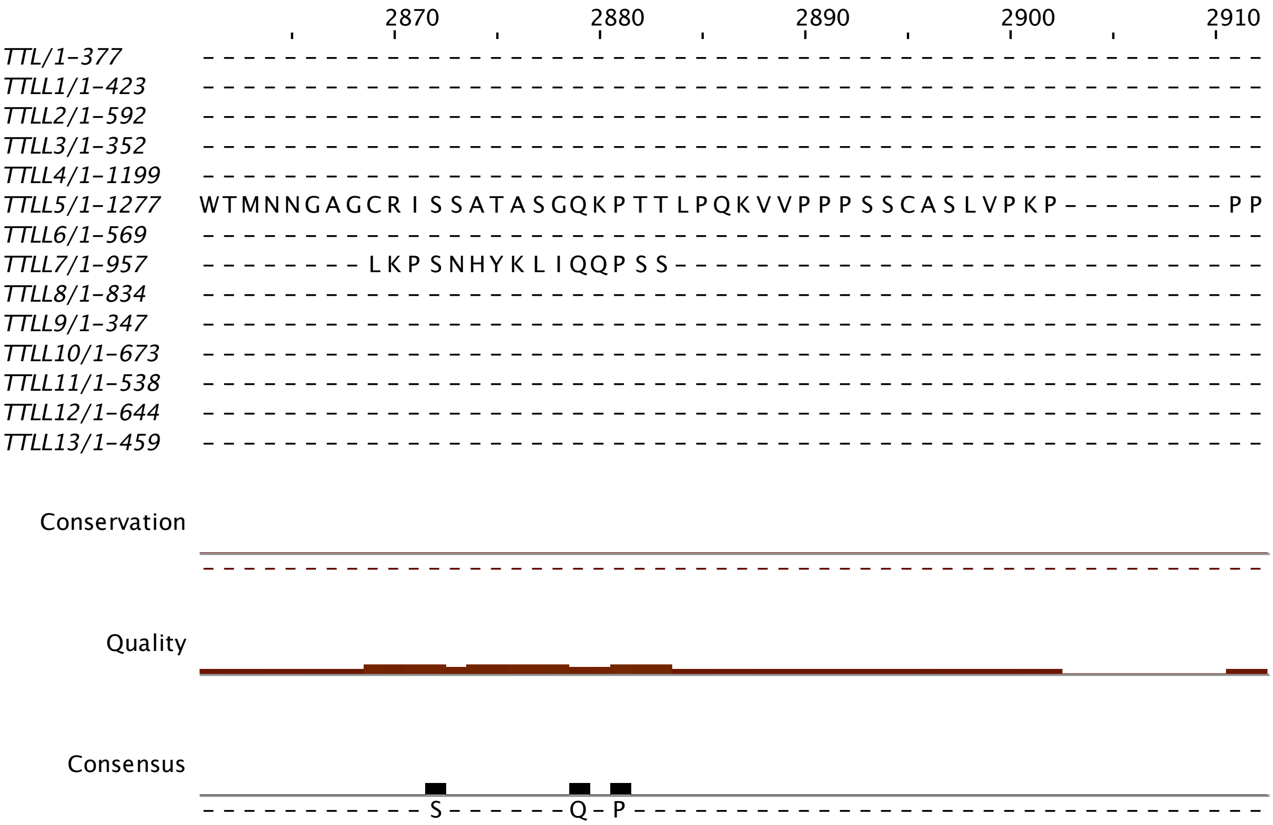
2870 2880 2890 2900 2910
TTL/1-377 --
TTLL1/1-423 --
TTLL2/1-592 --
TTLL3/1-352 --
TTLL4/1-1199 --
TTLL5/1-1277 WTMNNGAGCRISSATASGQKPTTLPQKVVPPPSSCASLVPKP--------PP
TTLL6/1-569 --
TTLL7/1-957 --------LKPSNHYKLIQQPSS-----------------------------
TTLL8/1-834 --
TTLL9/1-347 --
TTLL10/1-673 --
TTLL11/1-538 --
TTLL12/1-644 --
TTLL13/1-459 --
Conservation
Quality
Consensus
-----------S------Q-P-------------------------------

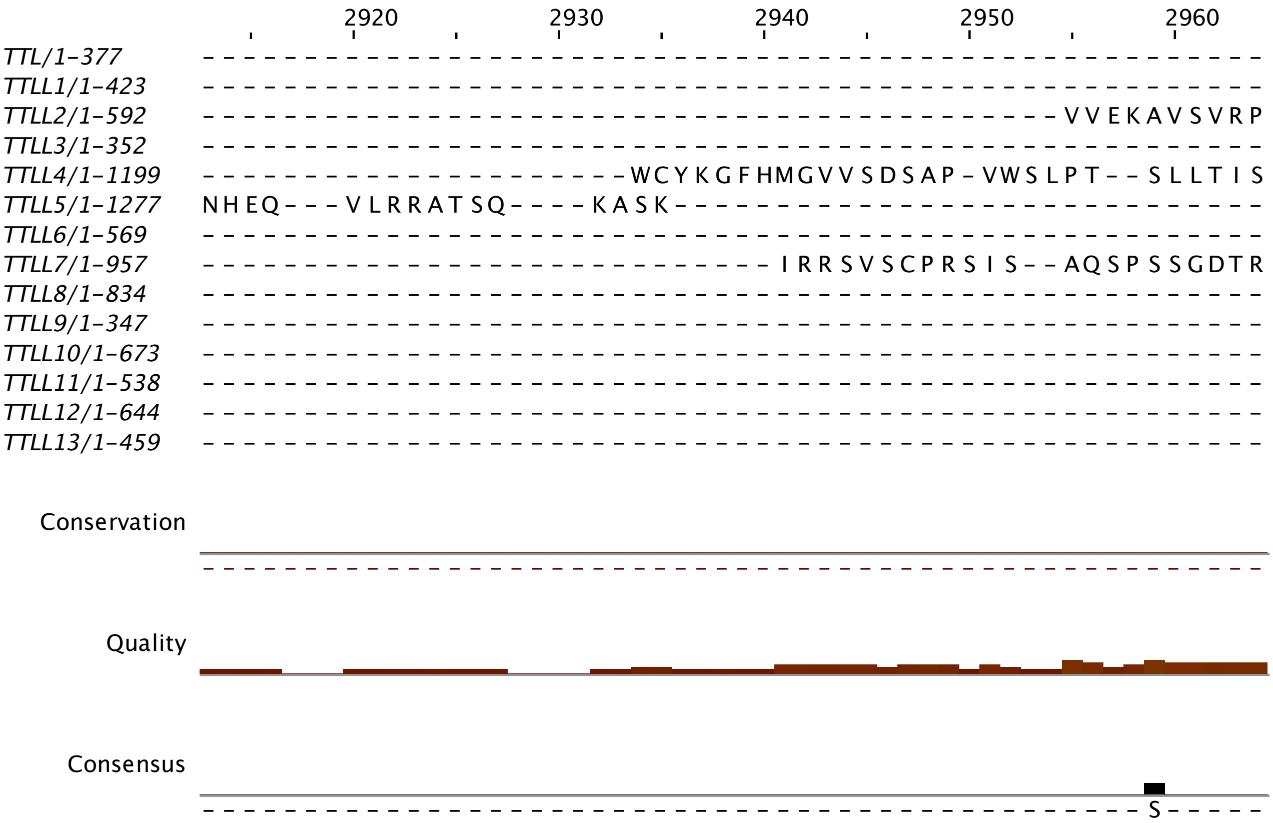

2920
2930
2940
2950
2960
TTL/1-377
TTL1/1-423
TTLL2/1-592 VVEKAVSVRP
TTLL3/1-352
TTLL4/1-1199 WCYKGFHMGVVSDSAP-VWSLPT--SLLTIS
TTLL5/1-1277 NHEQ---VLRRATSQ----KASK
TTLL6/1-569
TTLL7/1-957 IRRSVSCPRSIS--AQSPSSGDTR
TTLL8/1-834
TTLL9/1-347
TTLL10/1-673
TTLL11/1-538
TTLL12/1-644
TTLL13/1-459
Conservation
Quality
Consensus
S

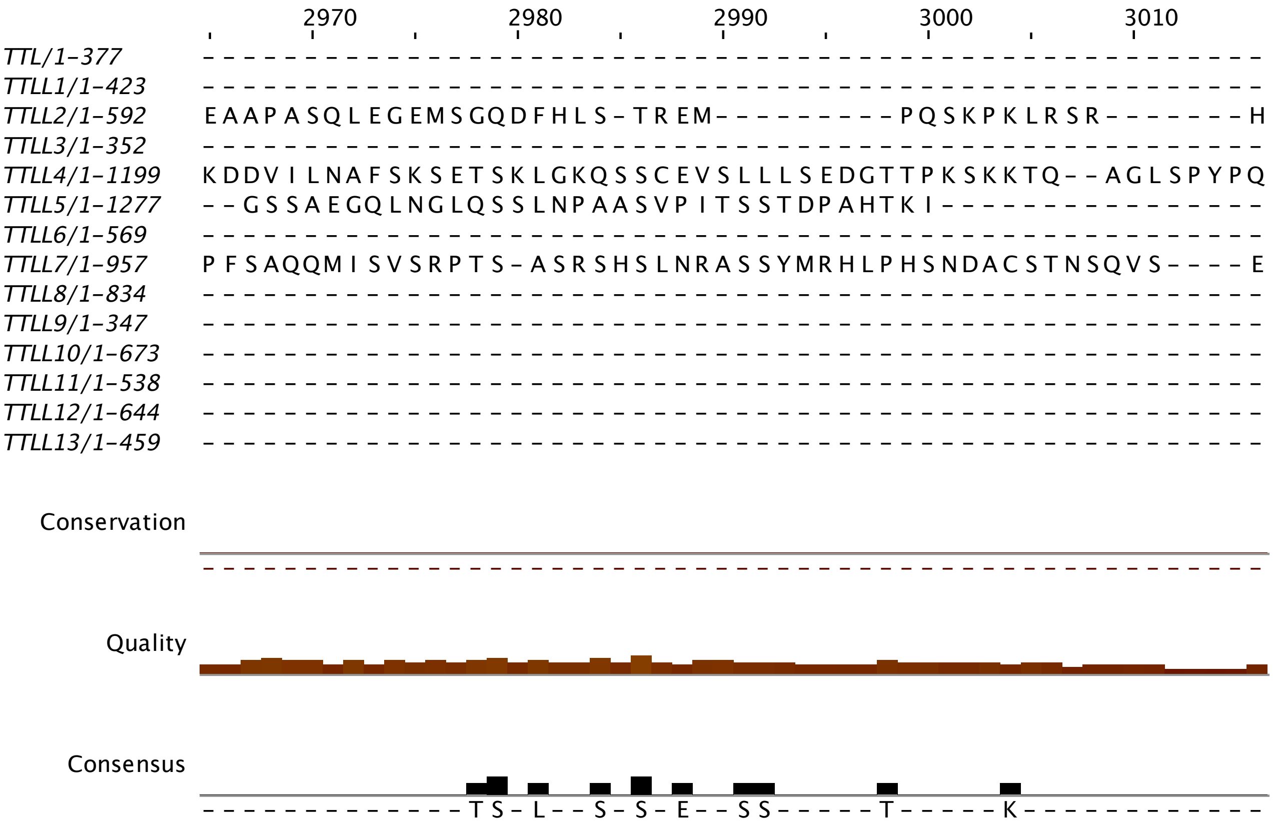
2970 2980 2990 3000 3010
TTL/1-377 --
TTLL1/1-423 --
TTLL2/1-592 EAAPASQLEGEMSGQDFHLS-TREM---------PQSKPKLRSR-------H
TTLL3/1-352 --
TTLL4/1-1199 KDDVILNAFSKSETSKLGKQSSCEVSLLLSEDGTTPKSKKTQ--AGLSPYPQ
TTLL5/1-1277 --GSSAEGQLNGLQSSLNPAASVPITSSTDPAHTKI----------------
TTLL6/1-569 --
TTLL7/1-957 PFSAQQMISVSRPTS-ASRSHSLNRASSYMRHLPHSNDACSTNSQVS----E
TTLL8/1-834 --
TTLL9/1-347 --
TTLL10/1-673 --
TTLL11/1-538 --
TTLL12/1-644 --
TTLL13/1-459 --
Conservation

Quality
Consensus
-------------TS-L--S-S-E--SS-----T-----K------------

```
              3020      3030      3040      3050      3060
TTL/1-377     ----------------------------------------------------
TTLL1/1-423   ----------------------------------------------------
TTLL2/1-592   TP--HKTLMPYASLFQSHSCKTK-------------------------TS--
TTLL3/1-352   ----------------------------------------------------
TTLL4/1-1199  KPSSSKDSEDTSKEPSLSTQTLPVIKCSGQTSRLSASSTFQSISDSLLAVSP
TTLL5/1-1277  ----------------------------------------------------
TTLL6/1-569   ----------------------------------------------------
TTLL7/1-957   SLRQLKTKEQEDDLTSQTLFVLKDMKIR-------------FPGKSDAESEL
TTLL8/1-834   ----------------------------------------------------
TTLL9/1-347   ----------------------------------------------------
TTLL10/1-673  ----------------------------------------------------
TTLL11/1-538  ----------------------------------------------------
TTLL12/1-644  ----------------------------------------------------
TTLL13/1-459  ----------------------------------------------------

Conservation
              ----------------------------------------------------

Quality

Consensus
              -P---KT-E----L-S-----LK--K-------------------S---S--
```

```
                3070      3080      3090      3100      3110
TTL/1-377       ----------------------------------------------------
TTLL1/1-423     ----------------------------------------------------
TTLL2/1-592     ----------------------------------------PCVLSDRGK---
TTLL3/1-352     ----------------------------------------------------
TTLL4/1-1199    ----------------------------------------------------
TTLL5/1-1277    ----------------------------------------------------
TTLL6/1-569     ----------------------------------------------------
TTLL7/1-957     L-IEDIIDNWKYHKTKVASYWLIKLDSVKQRK-------------------V
TTLL8/1-834     ----------------------------------------------------
TTLL9/1-347     ----------------------------------------------------
TTLL10/1-673    ----------------------------------------------------
TTLL11/1-538    ----------------------------------------------------
TTLL12/1-644    ----------------------------------------------------
TTLL13/1-459    ----------------------------------------------------

Conservation
                ----------------------------------------------------

Quality

Consensus
                ----------------------------------------------------
```

```
                 3130      3140      3150      3160      3170
TTL/1-377     ----------------------------------------------------
TTLL1/1-423   ----------------------------------------------------
TTLL2/1-592   ----------------------------------------------------
TTLL3/1-352   ----------------------------------------------------
TTLL4/1-1199  ----------------------------------------------------
TTLL5/1-1277  ----------------------------------------------------
TTLL6/1-569   ----------------------------------------------------
TTLL7/1-957   LDIVKTSIRTVLPRIWKVPDVEEVNLYRIFNRVFNRLLWSR-----------
TTLL8/1-834   ----------------------------------------------------
TTLL9/1-347   ----------------------------------------------------
TTLL10/1-673  ----------------------------------------------------
TTLL11/1-538  ----------------------------------------------------
TTLL12/1-644  ----------------------------------------------------
TTLL13/1-459  ----------------------------------------------------
```

Conservation

```
              ----------------------------------------------------
```

Quality

Consensus

```
              ----------------------------------------------------
```

	3180 3190 3200 3210 3220
TTL/1-377	--
TTLL1/1-423	--
TTLL2/1-592	--
TTLL3/1-352	--
TTLL4/1-1199	--
TTLL5/1-1277	--
TTLL6/1-569	--
TTLL7/1-957	-----GQGLWNCFCDSGSSWESIFNKSPEVVTPLQLQCCQRLVELCKQCLLV
TTLL8/1-834	--
TTLL9/1-347	--
TTLL10/1-673	--
TTLL11/1-538	--
TTLL12/1-644	--
TTLL13/1-459	--
Conservation	--
Quality	
Consensus	--

```
                3230      3240      3250      3260      3270
TTL/1-377       ----------------------------------------------------
TTLL1/1-423     ----------------------------------------------------
TTLL2/1-592     --APDPQAGNFVLVFPFNEATLGASRNGLNVKRIIQELQKLMNKQHS-----
TTLL3/1-352     ----------------------------------------------------
TTLL4/1-1199    ----------------------------------------------------
TTLL5/1-1277    ----------------------------------------------------
TTLL6/1-569     ----------------------------------------------------
TTLL7/1-957     VYKYATDKRGSLSGIGPDWGNSRYLLPGSTQFFLRTPTYNLKYNSPG-----
TTLL8/1-834     ----------------------------------------------------
TTLL9/1-347     ----------------------------------------------------
TTLL10/1-673    ----------------------------------------------------
TTLL11/1-538    ----------------------------------------------------
TTLL12/1-644    ----------------------------------------------------
TTLL13/1-459    ----------------------------------------------------

Conservation
                ----------------------------------------------------

Quality

Consensus
                ---------------------------G------------L-----------
```

```
                 3280       3290
           '    '    '    '    '
TTL/1-377        ----------------------
TTLL1/1-423      ----------------------
TTLL2/1-592      ----------------------
TTLL3/1-352      ----------------------
TTLL4/1-1199     ----------------------
TTLL5/1-1277     ----------------------
TTLL6/1-569      ----------------------
TTLL7/1-957      -------MTRSNVLFTSRYGHL
TTLL8/1-834      ----------------------
TTLL9/1-347      ----------------------
TTLL10/1-673     ----------------------
TTLL11/1-538     ----------------------
TTLL12/1-644     ----------------------
TTLL13/1-459     ----------------------

Conservation
                 ----------------------

Quality

Consensus
                 ----------------------
```
