# Supplementary material for: Tubulin Tyrosine Ligase Like 12, a TTLL Family Member with SET- and TTL-Like Domains and Roles in Histone and Tubulin Modifications and Mitosis
Source: PLoS One. 2012 Dec 12;7(12):e51258. doi: 10.1371/journal.pone.0051258 (PMC3520985; doi:10.1371/journal.pone.0051258)
Supplement: Alignment S2 — Multiple sequence alignment of the hTTLL12 SET-like domain and the SET domains of human proteins. (PDF) [file pone.0051258.s012.pdf]

ALIGNMENT S2

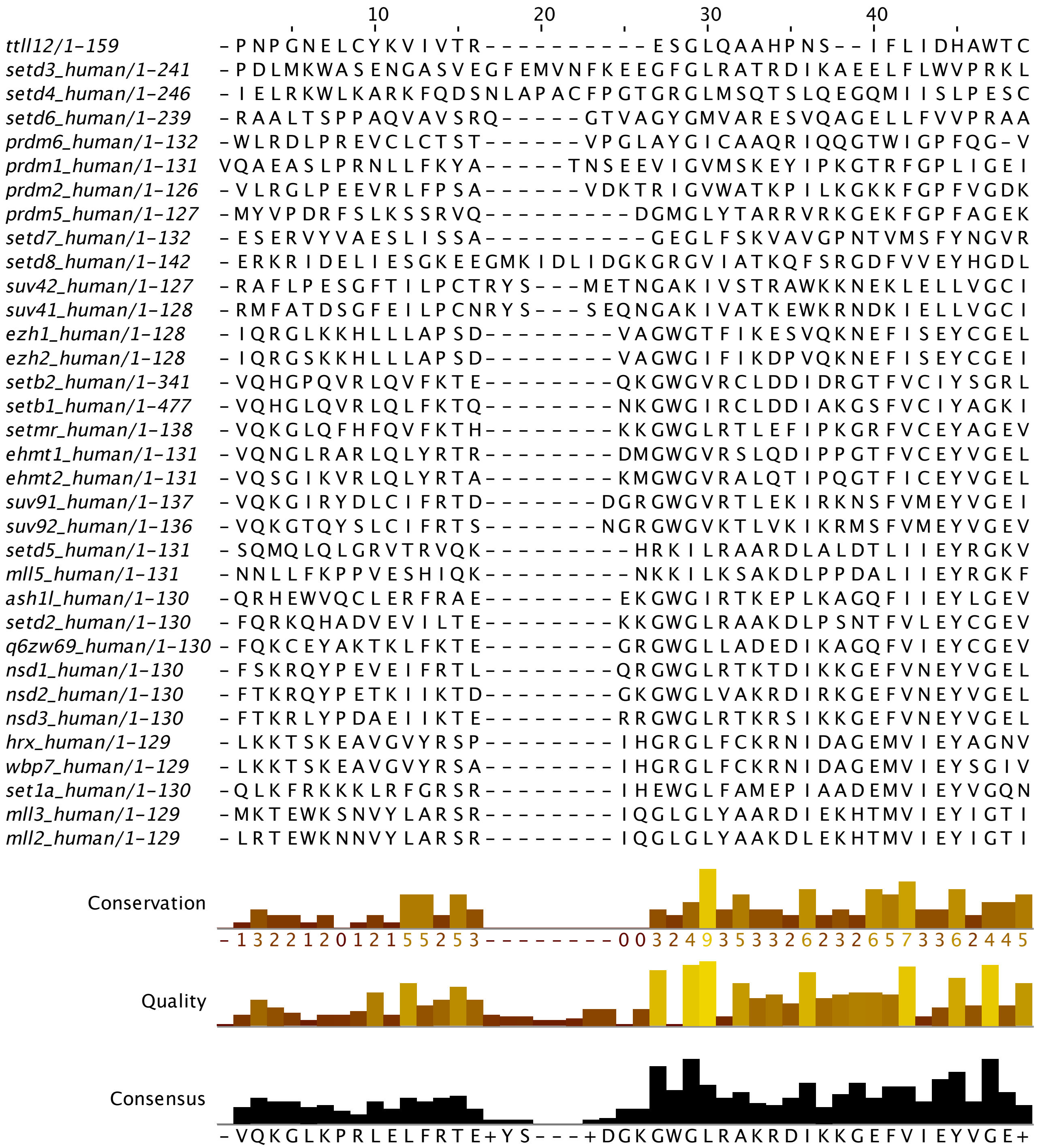
10 20 30 40
ttll12/1-159 -PNPGNELCYKVIVTR----------ESGLQAAHPNS--IFLIDHAWTC
setd3_human/1-241 -PDLMKWASENGASVEGFEMVNFKEEGFGLRATRDIKAEELFLWVPRKL
setd4_human/1-246 -IELRKWLKARKFQDSNLAPACFPGTGRGLMSQTSLQEGQMIISLPESC
setd6_human/1-239 -RAALTSPPAQVAVSRQ-----GTVAGYGMVARESVQAGELLFVVPRAA
prdm6_human/1-132 -WLRDLPREVCLCTST------VPGLAYGICAAQRIQQGTWIGPFQG-V
prdm1_human/1-131 VQAEASLPRNLLFKYA-----TNSEEVIGVMSKEYIPKGTRFGPLIGEI
prdm2_human/1-126 -VLRGLPEEVRLFPSA------VDKTRIGVWATKPILKGKKFGPFVGDK
prdm5_human/1-127 -MYVPDRFSLKSSRVQ---------DGMGLYTARRVRKGEKFGPFAGEK
setd7_human/1-132 -ESERVYYVAESLISSA----------GEGLFSKVAVGPNTVMSFYNGVR
setd8_human/1-142 -ERKRIDELIESGKEEGMKIDLIDGKGRGVIATKQFSRGDFVVEYHGDL
suv42_human/1-127 -RAFLPESGFTILPCTRYS---METNGAKIVSTRAWKKNEKLELLVGCI
suv41_human/1-128 -RMFATDSGFEILPCNRYS---SEQNGAKIVATKEWKRNDKIELLVGCI
ezh1_human/1-128 -IQRGLKKHLLLAPSD--------VAGWGTFIKESVQKNEFISEYCGEL
ezh2_human/1-128 -IQRGSKKHLLLAPSD--------VAGWGIFIKDPVQKNEFISEYCGEI
setb2_human/1-341 -VQHGPQVRLQVFKTE--------QKGWGVRCLDDIDRGTFVCIYSGRL
setb1_human/1-477 -VQHGLQVRLQLFKTQ--------NKGWGIRCLDDIAKGSFVCIYAGKI
setmr_human/1-138 -VQKGLQFHFQVFKTH--------KKGWGLRTLEFIPKGRFVCEYAGEV
ehmt1_human/1-131 -VQNGLRARLQLYRTR--------DMGWGVRSLQDIPPGTFVCEYVGEL
ehmt2_human/1-131 -VQSGIKVRLQLYRTA--------KMGWGVRALQTIPQGTFICEYVGEL
suv91_human/1-137 -VQKGIRYDLCIFRTD-------DGRGWGVRTLEKIRKNSFVMEYVGEI
suv92_human/1-136 -VQKGTQYSLCIFRTS-------NGRGWGVKTLVKIKRMSFVMEYVGEV
setd5_human/1-131 -SQMQLQLGRVTRVQK---------HRKILRAARDLALDTLIIEYRGKV
mll5_human/1-131 -NNLLFKPPVESHIQK---------NKKILKSAKDLPPDALIIEYRGKF
ash1l_human/1-130 -QRHEWVQCLERFRAE--------EKGWGIRTKEPLKAGQFIIEYLGEV
setd2_human/1-130 -FQRKQHADVEVILTE--------KKGWGLRAAKDLPSNTFVLEYCGEV
q6zw69_human/1-130 -FQKCEYAKTKLFKTE--------GRGWGLLADEDIKAGQFVIEYCGEV
nsd1_human/1-130 -FSKRQYPEVEIFRTL--------QRGWGLRTKTDIKKGEFVNEYVGEL
nsd2_human/1-130 -FTKRQYPETKIIKTD--------GKGWGLVAKRDIRKGEFVNEYVGEL
nsd3_human/1-130 -FTKRLYPDAEIIKTE--------RRGWGLRTKRSIKKGEFVNEYVGEL
hrx_human/1-129 -LKKTSKEAVGVYRSP--------IHGRGLFCKRNIDAGEMVIEYAGNV
wbp7_human/1-129 -LKKTSKEAVGVYRSA--------IHGRGLFCKRNIDAGEMVIEYSGIV
set1a_human/1-130 -QLKFRKKKLRFGRSR--------IHEWGLFAMEPIAADEMVIEYVGQN
mll3_human/1-129 -MKTEWKSNVYLARSR--------IQGLGLYAARDIEKHTMVIEYIGTI
mll2_human/1-129 -LRTEWKNNVYLARSR--------IQGLGLYAAKDLEKHTMVIEYIGTI
Conservation
-132212012155253--------003249353326232657336 2445
Quality
Consensus
-VQKGLKPRLELFRTE+YS---+DGKGWGLRAKRDIKKGEFVIEYVGE+

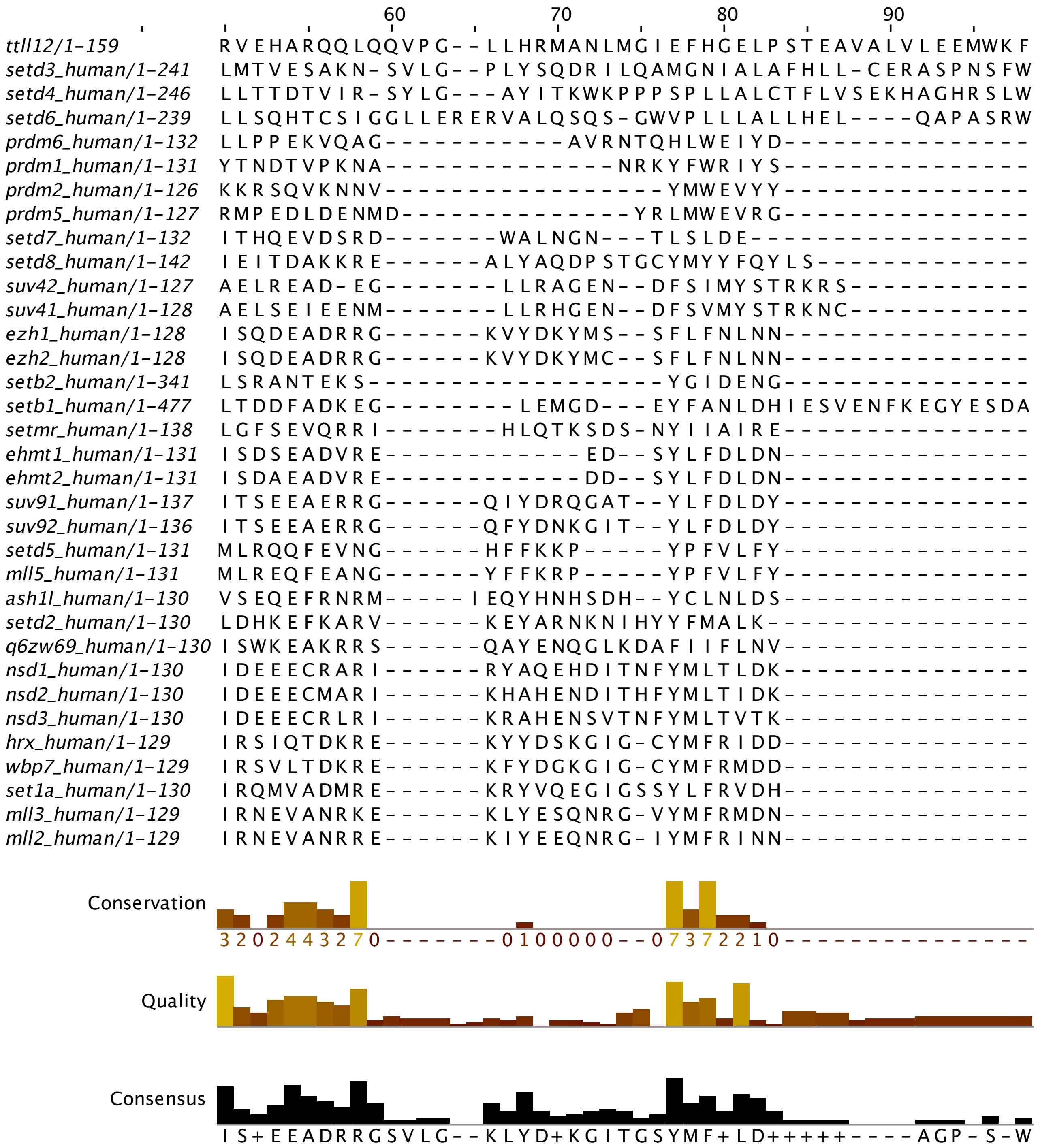

60
70
80
90
ttll12/1-159 RVEHARQQLQQVPG--LLHRMANLMGIEFHGELPSTEAVALVLEEMWKF
setd3_human/1-241 LMTVESAKN-SVLG--PLYSQDRILQAMGNIALAFHLL-CERASPNSFW
setd4_human/1-246 LLTTDTVIR-SYLG---AYITKWKPPPSPLLALCTFLVSEKHAGHRSLW
setd6_human/1-239 LLSQHTCSIGGLLERERVALQSQS-GWVPLLLALLHEL----QAPASRW
prdm6_human/1-132 LLPPEKVQAG-----------AVRNTQHLWEIYD---------------
prdm1_human/1-131 YTNDTVPKNA--------------NRKYFWRIYS---------------
prdm2_human/1-126 KKRSQVKNNV-----------------YMWEVYY---------------
prdm5_human/1-127 RMPEDLDENMD--------------YRLMWEVRG---------------
setd7_human/1-132 ITHQEVDSRD-------WALNGN---TLSLDE-----------------
setd8_human/1-142 IEITDAKKRE------ALYAQDPSTGCYMYYFQYLS-------------
suv42_human/1-127 AELREAD-EG-------LLRAGEN--DFSIMYSTRKRS-----------
suv41_human/1-128 AELSEIEENM-------LLRHGEN--DFSVMYSTRKNC-----------
ezh1_human/1-128 ISQDEADRRG------KVYDKYMS--SFLFNLNN---------------
ezh2_human/1-128 ISQDEADRRG------KVYDKYMC--SFLFNLNN---------------
setb2_human/1-341 LSRANTEKS------------------YGIDENG---------------
setb1_human/1-477 LTDDFADKEG--------LEMGD---EYFANLDHIESVENFKEGYESDA
setmr_human/1-138 LGFSEVQRRI-------HLQTKSDS-NYIIAIRE---------------
ehmt1_human/1-131 ISDSEADVRE------------ED--SYLFDLDN---------------
ehmt2_human/1-131 ISDAEADVRE------------DD--SYLFDLDN---------------
suv91_human/1-137 ITSEEAERRG------QIYDRQGAT--YLFDLDY---------------
suv92_human/1-136 ITSEEAERRG------QFYDNKGIT--YLFDLDY---------------
setd5_human/1-131 MLRQQFEVNG------HFFKKP-----YPFVLFY---------------
mll5_human/1-131 MLREQFEANG------YFFKRP-----YPFVLFY---------------
ash1l_human/1-130 VSEQEFRNRM-----IEQYHNHSDH--YCLNLDS---------------
setd2_human/1-130 LDHKEFKARV------KEYARNKNIHYYFMALK----------------
q6zw69_human/1-130 ISWKEAKRRS------QAYENQGLKDAFIIFLNV---------------
nsd1_human/1-130 IDEEECRARI------RYAQEHDITNFYMLTLDK---------------
nsd2_human/1-130 IDEEECMARI------KHAHENDITHFYMLTIDK---------------
nsd3_human/1-130 IDEEECRLRI------KRAHENSVTNFYMLTVTK---------------
hrx_human/1-129 IRSIQTDKRE------KYYDSKGIG-CYMFRIDD---------------
wbp7_human/1-129 IRSVLTDKRE------KFYDGKGIG-CYMFRMDD---------------
set1a_human/1-130 IRQMVADMRE------KRYVQEGIGSSYLFRVDH---------------
mll3_human/1-129 IRNEVANRKE------KLYESQNRG-VYMFRMDN---------------
mll2_human/1-129 IRNEVANRRE------KIYEEQNRG-IYMFRINN---------------
Conservation
3202443270-------0100000--07372210---------------
Quality
Consensus
IS+EEADRRGSVLG--KLYD+KGITGSYMF+LD+++++----AGP-S-W

```
                        100       110       120       130       140
ttll12/1-159          NQTYQLAH-----------------------------------------
setd3_human/1-241     QPYIQTLPSEYD--TPLYFEEDEVRY--LQSTQAIHDVFSQYKNTARQY
setd4_human/1-246     KPYLEILPKAYT--CPVCLEPEVVNL--LPKSLKAKAEEQRAHVQEFFA
setd6_human/1-239     RPYFALWPELGRLEHPMFWPEEERRCLLQGTGVPEAVEKDLANISSEYQ
prdm6_human/1-132     -------------------------------------------------
prdm1_human/1-131     -------------------------------------------------
prdm2_human/1-126     -------------------------------------------------
prdm5_human/1-127     -------------------------------------------------
setd7_human/1-132     -------------------------------------------------
setd8_human/1-142     -------------------------------------------------
suv42_human/1-127     -------------------------------------------------
suv41_human/1-128     -------------------------------------------------
ezh1_human/1-128      -------------------------------------------------
ezh2_human/1-128      -------------------------------------------------
setb2_human/1-341     -------------------------------------------------
setb1_human/1-477     PCSSDSSGVDLKDQEDGNSGTEDPEESNDDSSDDNFCKDEDFSTSSVWR
setmr_human/1-138     -------------------------------------------------
ehmt1_human/1-131     -------------------------------------------------
ehmt2_human/1-131     -------------------------------------------------
suv91_human/1-137     -------------------------------------------------
suv92_human/1-136     -------------------------------------------------
setd5_human/1-131     -------------------------------------------------
mll5_human/1-131      -------------------------------------------------
ash1l_human/1-130     -------------------------------------------------
setd2_human/1-130     -------------------------------------------------
q6zw69_human/1-130    -------------------------------------------------
nsd1_human/1-130      -------------------------------------------------
nsd2_human/1-130      -------------------------------------------------
nsd3_human/1-130      -------------------------------------------------
hrx_human/1-129       -------------------------------------------------
wbp7_human/1-129      -------------------------------------------------
set1a_human/1-130     -------------------------------------------------
mll3_human/1-129      -------------------------------------------------
mll2_human/1-129      -------------------------------------------------
```

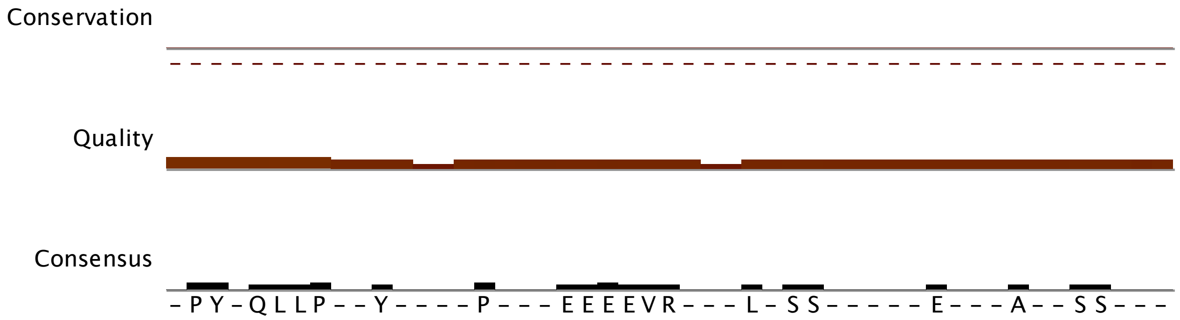

```
                          150       160       170       180       190
ttll12/1-159          -------------------------------------------------
setd3_human/1-241     AYFYKVIQTHPHANKLPLKDSFTYEDYRWAVSSVMTRQNQI--------
setd4_human/1-246     SSRDFFSSLQPL-FAEAVDSIFSYSALLWAWCTVNTRAVYL--------
setd6_human/1-239     SIVLPFMEAHPDLFSLGVRSLELYHQLVALVMAYSFQ------------
prdm6_human/1-132     -------------------------------------------------
prdm1_human/1-131     -------------------------------------------------
prdm2_human/1-126     -------------------------------------------------
prdm5_human/1-127     -------------------------------------------------
setd7_human/1-132     -------------------------------------------------
setd8_human/1-142     -------------------------------------------------
suv42_human/1-127     -------------------------------------------------
suv41_human/1-128     -------------------------------------------------
ezh1_human/1-128      -------------------------------------------------
ezh2_human/1-128      -------------------------------------------------
setb2_human/1-341     -----RDENTMKNIFSKKRKLEVACSDCEVEVLPLGLETHPRTAKTEKC
setb1_human/1-477     SYATRRQTRGQKENGLSETTSKDSHPPDLGPPHIPVPPSIPVGGCNPPS
setmr_human/1-138     -------------------------------------------------
ehmt1_human/1-131     -------------------------------------------------
ehmt2_human/1-131     -------------------------------------------------
suv91_human/1-137     -------------------------------------------------
suv92_human/1-136     -------------------------------------------------
setd5_human/1-131     SKFNGV-------------------------------------------
mll5_human/1-131      SKFHGL-------------------------------------------
ash1l_human/1-130     -------------------------------------------------
setd2_human/1-130     -------------------------------------------------
q6zw69_human/1-130    -------------------------------------------------
nsd1_human/1-130      -------------------------------------------------
nsd2_human/1-130      -------------------------------------------------
nsd3_human/1-130      -------------------------------------------------
hrx_human/1-129       -------------------------------------------------
wbp7_human/1-129      -------------------------------------------------
set1a_human/1-130     -------------------------------------------------
mll3_human/1-129      -------------------------------------------------
mll2_human/1-129      -------------------------------------------------

Conservation
                      -------------------------------------------------
```

Quality

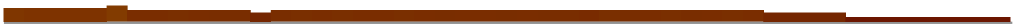

Consensus

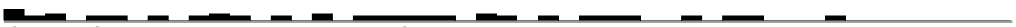

```
S+F-G+-E-HPK-+-L-VRS++-YH-L-WAV--V-TR---P--------
```

```
                        200        210        220        230        240
ttll12/1-159        -------------------------------------------------
setd3_human/1-241   -------------------------------------------------
setd4_human/1-246   -------------------------------------------------
setd6_human/1-239   -------------------------------------------------
prdm6_human/1-132   -------------------------------------------------
prdm1_human/1-131   -------------------------------------------------
prdm2_human/1-126   -------------------------------------------------
prdm5_human/1-127   -------------------------------------------------
setd7_human/1-132   -------------------------------------------------
setd8_human/1-142   -------------------------------------------------
suv42_human/1-127   -------------------------------------------------
suv41_human/1-128   -------------------------------------------------
ezh1_human/1-128    -------------------------------------------------
ezh2_human/1-128    -------------------------------------------------
setb2_human/1-341   PPKFSNNPKEL------------------------------TMETKYDN
setb1_human/1-477   SEETPKNKVASWLSCNSVSEGGFADSDSHSSFKTNEGGEGRAGGSRMEA
setmr_human/1-138   -------------------------------------------------
ehmt1_human/1-131   -------------------------------------------------
ehmt2_human/1-131   -------------------------------------------------
suv91_human/1-137   -------------------------------------------------
suv92_human/1-136   -------------------------------------------------
setd5_human/1-131   -------------------------------------------------
mll5_human/1-131    -------------------------------------------------
ash1l_human/1-130   -------------------------------------------------
setd2_human/1-130   -------------------------------------------------
q6zw69_human/1-130  -------------------------------------------------
nsd1_human/1-130    -------------------------------------------------
nsd2_human/1-130    -------------------------------------------------
nsd3_human/1-130    -------------------------------------------------
hrx_human/1-129     -------------------------------------------------
wbp7_human/1-129    -------------------------------------------------
set1a_human/1-130   -------------------------------------------------
mll3_human/1-129    -------------------------------------------------
mll2_human/1-129    -------------------------------------------------

       Conservation
                    -------------------------------------------------

            Quality

          Consensus
                    ------N------------------------------------------
```

```
                        250       260       270       280       290
ttll12/1-159         -------------------------------------------------
setd3_human/1-241    -------------------------------------------------
setd4_human/1-246    -------------------------------------------------
setd6_human/1-239    -------------------------------------------------
prdm6_human/1-132    -------------------------------------------------
prdm1_human/1-131    -------------------------------------------------
prdm2_human/1-126    -------------------------------------------------
prdm5_human/1-127    -------------------------------------------------
setd7_human/1-132    -------------------------------------------------
setd8_human/1-142    -------------------------------------------------
suv42_human/1-127    -------------------------------------------------
suv41_human/1-128    -------------------------------------------------
ezh1_human/1-128     -------------------------------------------------
ezh2_human/1-128     -------------------------------------------------
setb2_human/1-341    ISRIQYHSVIRDPESKTAIFQHNGKKMEFVSSESVTPEDNDGFKPPREH
setb1_human/1-477    EKASTSGLGIKDEGDIKQAKKEDTDDRNKMSVVTESSRNYGYNPSPVKP
setmr_human/1-138    -------------------------------------------------
ehmt1_human/1-131    -------------------------------------------------
ehmt2_human/1-131    -------------------------------------------------
suv91_human/1-137    -------------------------------------------------
suv92_human/1-136    -------------------------------------------------
setd5_human/1-131    -------------------------------------------------
mll5_human/1-131     -------------------------------------------------
ash1l_human/1-130    -------------------------------------------------
setd2_human/1-130    -------------------------------------------------
q6zw69_human/1-130   -------------------------------------------------
nsd1_human/1-130     -------------------------------------------------
nsd2_human/1-130     -------------------------------------------------
nsd3_human/1-130     -------------------------------------------------
hrx_human/1-129      -------------------------------------------------
wbp7_human/1-129     -------------------------------------------------
set1a_human/1-130    -------------------------------------------------
mll3_human/1-129     -------------------------------------------------
mll2_human/1-129     -------------------------------------------------

Conservation
                     -------------------------------------------------

Quality

Consensus
                     ---------I-D------------------S---------------P---
```

```
                         300       310       320       330       340
ttll12/1-159          -------------------------------------------------
setd3_human/1-241     -------------------------------------------------
setd4_human/1-246     -------------------------------------------------
setd6_human/1-239     -------------------------------------------------
prdm6_human/1-132     -------------------------------------------------
prdm1_human/1-131     -------------------------------------------------
prdm2_human/1-126     -------------------------------------------------
prdm5_human/1-127     -------------------------------------------------
setd7_human/1-132     -------------------------------------------------
setd8_human/1-142     -------------------------------------------------
suv42_human/1-127     -------------------------------------------------
suv41_human/1-128     -------------------------------------------------
ezh1_human/1-128      -------------------------------------------------
ezh2_human/1-128      -------------------------------------------------
setb2_human/1-341     LNSKTKGAQKDSSSNHVDEFEDNLLIESDVIDITKYREETPPRSRCNQA
setb1_human/1-477     EGLRRPPSKTSMHQSRRLMASAQSNPDDVLTLSSSTESEGESGTSRKPT
setmr_human/1-138     -------------------------------------------------
ehmt1_human/1-131     -------------------------------------------------
ehmt2_human/1-131     -------------------------------------------------
suv91_human/1-137     -------------------------------------------------
suv92_human/1-136     -------------------------------------------------
setd5_human/1-131     -------------------------------------------------
mll5_human/1-131      -------------------------------------------------
ash1l_human/1-130     -------------------------------------------------
setd2_human/1-130     -------------------------------------------------
q6zw69_human/1-130    -------------------------------------------------
nsd1_human/1-130      -------------------------------------------------
nsd2_human/1-130      -------------------------------------------------
nsd3_human/1-130      -------------------------------------------------
hrx_human/1-129       -------------------------------------------------
wbp7_human/1-129      -------------------------------------------------
set1a_human/1-130     -------------------------------------------------
mll3_human/1-129      -------------------------------------------------
mll2_human/1-129      -------------------------------------------------

Conservation
                      -------------------------------------------------

Quality

Consensus
                      --------------------------------------E----------
```

```
                            350       360       370       380       390
ttll12/1-159         -------------------------------------------------
setd3_human/1-241    -------------------------------------------------
setd4_human/1-246    -------------------------------------------------
setd6_human/1-239    -------------------------------------------------
prdm6_human/1-132    -------------------------------------------------
prdm1_human/1-131    -------------------------------------------------
prdm2_human/1-126    -------------------------------------------------
prdm5_human/1-127    -------------------------------------------------
setd7_human/1-132    -------------------------------------------------
setd8_human/1-142    -------------------------------------------------
suv42_human/1-127    -------------------------------------------------
suv41_human/1-128    -------------------------------------------------
ezh1_human/1-128     -------------------------------------------------
ezh2_human/1-128     -------------------------------------------------
setb2_human/1-341    TTL------------------------------DNQNIKKAIEVQIQKP
setb1_human/1-477    AGQTSATAVDSDDIQTISSGSEGDDFEDKKNMTGPMKRQVAVKSTRGFA
setmr_human/1-138    -------------------------------------------------
ehmt1_human/1-131    -------------------------------------------------
ehmt2_human/1-131    -------------------------------------------------
suv91_human/1-137    -------------------------------------------------
suv92_human/1-136    -------------------------------------------------
setd5_human/1-131    -------------------------------------------------
mll5_human/1-131     -------------------------------------------------
ash1l_human/1-130    -------------------------------------------------
setd2_human/1-130    -------------------------------------------------
q6zw69_human/1-130   -------------------------------------------------
nsd1_human/1-130     -------------------------------------------------
nsd2_human/1-130     -------------------------------------------------
nsd3_human/1-130     -------------------------------------------------
hrx_human/1-129      -------------------------------------------------
wbp7_human/1-129     -------------------------------------------------
set1a_human/1-130    -------------------------------------------------
mll3_human/1-129     -------------------------------------------------
mll2_human/1-129     -------------------------------------------------

Conservation
                     -------------------------------------------------

Quality

Consensus
                     ----------------------------------------A--------
```

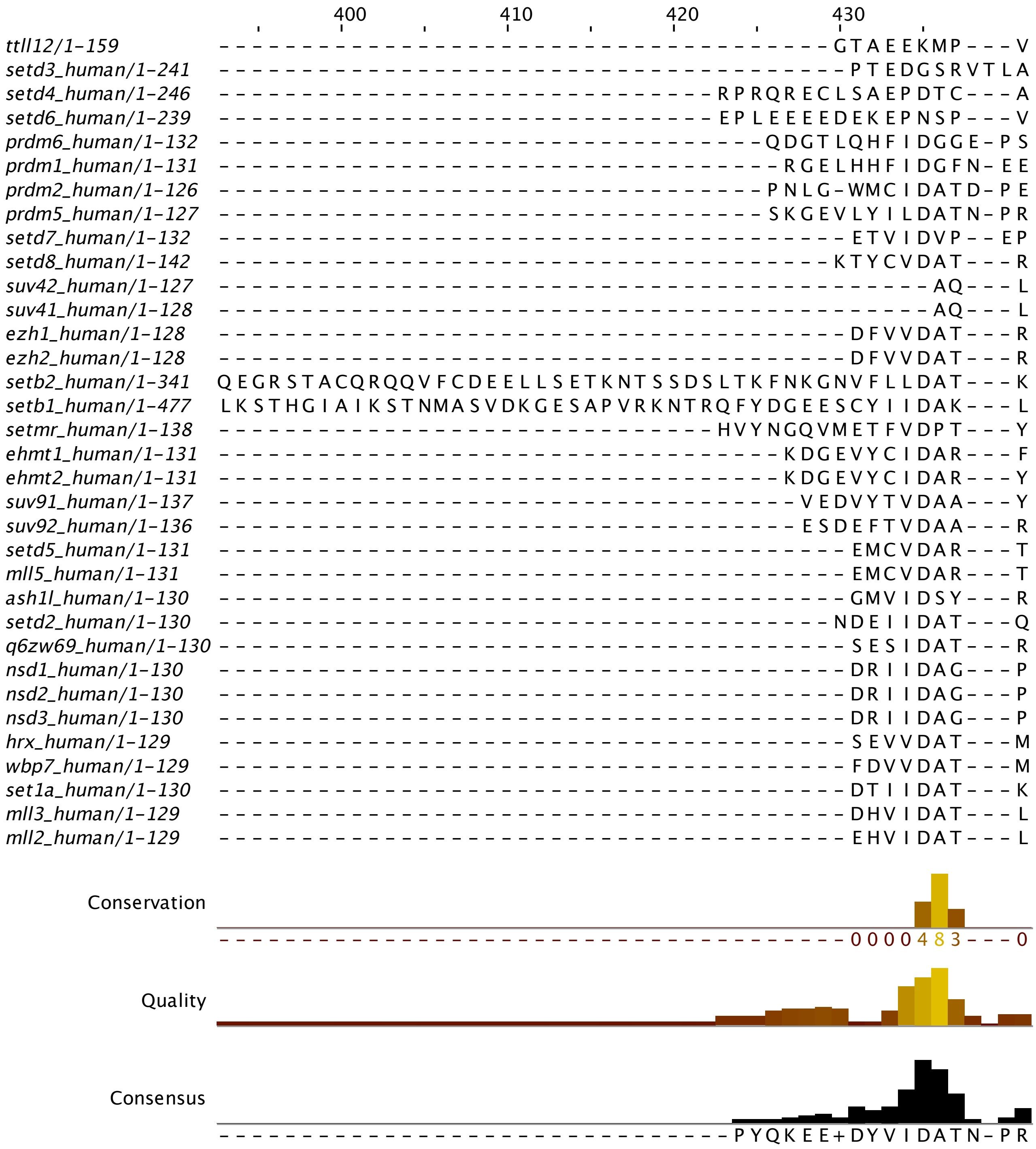
400 410 420 430
ttll12/1-159 -------------------------------------GTAEEKMP---V
setd3_human/1-241 --------------------------------------PTEDGSRVTLA
setd4_human/1-246 ------------------------------RPRQRECLSAEPDTC---A
setd6_human/1-239 ------------------------------EPLEEEEDEKEPNSP---V
prdm6_human/1-132 ---------------------------------QDGTLQHFIDGGE-PS
prdm1_human/1-131 ----------------------------------RGELHHFIDGFN-EE
prdm2_human/1-126 ---------------------------------PNLG-WMCIDATD-PE
prdm5_human/1-127 ---------------------------------SKGEVLYILDATN-PR
setd7_human/1-132 --------------------------------------ETVIDVP--EP
setd8_human/1-142 -------------------------------------KTYCVDAT---R
suv42_human/1-127 -------------------------------------------AQ---L
suv41_human/1-128 -------------------------------------------AQ---L
ezh1_human/1-128 --------------------------------------DFVVDAT---R
ezh2_human/1-128 --------------------------------------DFVVDAT---R
setb2_human/1-341 QEGRSTACQRQQVFCDEELLSETKNTSSDSLTKFNKGNVFLLDAT---K
setb1_human/1-477 LKSTHGIAIKSTNMASVDKGESAPVRKNTRQFYDGEESCYIIDAK---L
setmr_human/1-138 ------------------------------HVYNGQVMETFVDPT---Y
ehmt1_human/1-131 ----------------------------------KDGEVYCIDAR---F
ehmt2_human/1-131 ----------------------------------KDGEVYCIDAR---Y
suv91_human/1-137 -----------------------------------VEDVYTVDAA---Y
suv92_human/1-136 -----------------------------------ESDEFTVDAA---R
setd5_human/1-131 --------------------------------------EMCVDAR---T
mll5_human/1-131 --------------------------------------EMCVDAR---T
ash1l_human/1-130 --------------------------------------GMVIDSY---R
setd2_human/1-130 -------------------------------------NDEIIDAT---Q
q6zw69_human/1-130 --------------------------------------SESIDAT---R
nsd1_human/1-130 --------------------------------------DRIIDAG---P
nsd2_human/1-130 --------------------------------------DRIIDAG---P
nsd3_human/1-130 --------------------------------------DRIIDAG---P
hrx_human/1-129 --------------------------------------SEVVDAT---M
wbp7_human/1-129 --------------------------------------FDVVDAT---M
set1a_human/1-130 --------------------------------------DTIIDAT---K
mll3_human/1-129 --------------------------------------DHVIDAT---L
mll2_human/1-129 --------------------------------------EHVIDAT---L
Conservation
--------------------------------------0000483---0
Quality
Consensus
-------------------------------PYQKEE+DYVIDATN-PR

```
                     450       460       470       480
ttll12/1-159         WYI-MDEFGSRI----QHADVPSFATAPFFYMP--QQVAYTLLWPLRDL
setd3_human/1-241    LIP-LWDMC-------NHTNGLITT---GYNL----EDDRCECVALQDF
setd4_human/1-246    LAP-YLDLL-------NHSPHVQVKA--AFNE----ETHSYEIRTTSRW
setd6_human/1-239    MVP-AADIL-------NHLANHNA----NLEY----SANCLRMVATQPI
prdm6_human/1-132    KSS-WMRYI-------RCARHCGEQNLTVVQY----RSN-IFYRACIDI
prdm1_human/1-131    KSN-WMRYV-------NPAHSPREQNLAACQN----GMN-IYFYTIKPI
prdm2_human/1-126    KGN-WLRYV-------NWACSGEEQNLFPLEI----NRA-IYYKTLKPI
prdm5_human/1-127    HSN-WLRFV-------HEAPSQEQKNLAAIQE----GEN-IFYLAVEDI
setd7_human/1-132    YNH-VSKYCASLGHKANHSFTPNCIYDMFVHPRF--GPIK-CIRTLRAV
setd8_human/1-142    ETNRLGRLI-------NHSKCGNCQTKLHDID----GVPHLILIASRDI
suv42_human/1-127    WLG-PAAFI-------NHDCKPNCKFVPA-------DGNAACVKVLRDI
suv41_human/1-128    WLG-PAAFI-------NHDCRPNCKFVST-------GRDTACVKALRDI
ezh1_human/1-128     KGN-KIRFA-------NHSVNPNCYAKVVMVN----GDHRIGIFAKRAI
ezh2_human/1-128     KGN-KIRFA-------NHSVNPNCYAKVMMVN----GDHRIGIFAKRAI
setb2_human/1-341    EGN-VGRFL-------NHSCCPNLLVQNVFVETHNRNFPLVAFFTNRYV
setb1_human/1-477    EGN-LGRYL-------NHSCSPNLFVQNVFVDTHDLRFPWVAFFASKRI
setmr_human/1-138    IGN-IGRFL-------NHSCEPNLLMIPVRIDS---MVPKLALFAAKDI
ehmt1_human/1-131    YGN-VSRFI-------NHHCEPNLVPVRVFMAHQDLRFPRIAFFSTRLI
ehmt2_human/1-131    YGN-ISRFI-------NHLCDPNIIPVRVFMLHQDLRFPRIAFFSSRDI
suv91_human/1-137    YGN-ISHFV-------NHSCDPNLQVYNVFIDNLDERLPRIAFFATRTI
suv92_human/1-136    YGN-VSHFV-------NHSCDPNLQVFNVFIDNLDTRLPRIALFSTRTI
setd5_human/1-131    FGN-DARFI-------RRSCTPNAEVRHMIAD----GMIHLCIYAVSAI
mll5_human/1-131     FGN-EARFI-------RRSRTPNAEVRHEIQD----GTIHLYIYSIHSI
ash1l_human/1-130    MGN-EARFI-------NHSCDPNCEMQKWSVN----GVYRIGLYALKDM
setd2_human/1-130    KGN-CSRFM-------NHSCEPNCETQKWTVN----GQLRVGFFTTKLV
q6zw69_human/1-130   KGS-LARFI-------NHSCQPNCETRKWNVL----GEIRVGIFAKHDI
nsd1_human/1-130     KGN-YARFM-------NHCCQPNCETQKWSVN----GDTRVGLFALSDI
nsd2_human/1-130     KGN-YSRFM-------NHSCQPNCETLKWTVN----GDTRVGLFAVCDI
nsd3_human/1-130     KGN-YSRFM-------NHSCNPNCETQKWTVN----GDVRVGLFALCDI
hrx_human/1-129      HGN-AARFI-------NHSCEPNCYSRVINID----GQKHIVIFAMRKI
wbp7_human/1-129     HGN-AARFI-------NHSCEPNCFSRVIHVE----GQKHIVIFALRRI
set1a_human/1-130    CGN-LARFI-------NHCCTPNCYAKVITIE----SQKKIVIYSKQPI
mll3_human/1-129     TGG-PARYI-------NHSCAPNCVAEVVTFE----RGHKIIISSSRRI
mll2_human/1-129     TGG-PARYI-------NHSCAPNCVAEVVTFD----KEDKIIIISSRRI
```

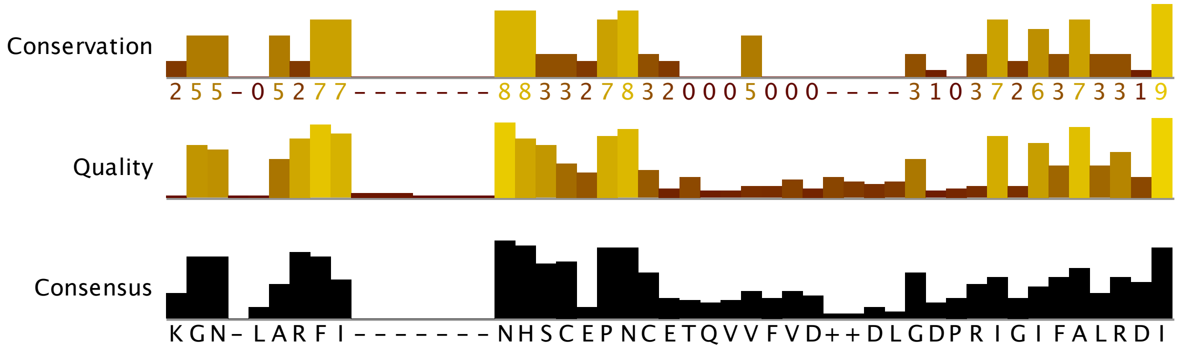

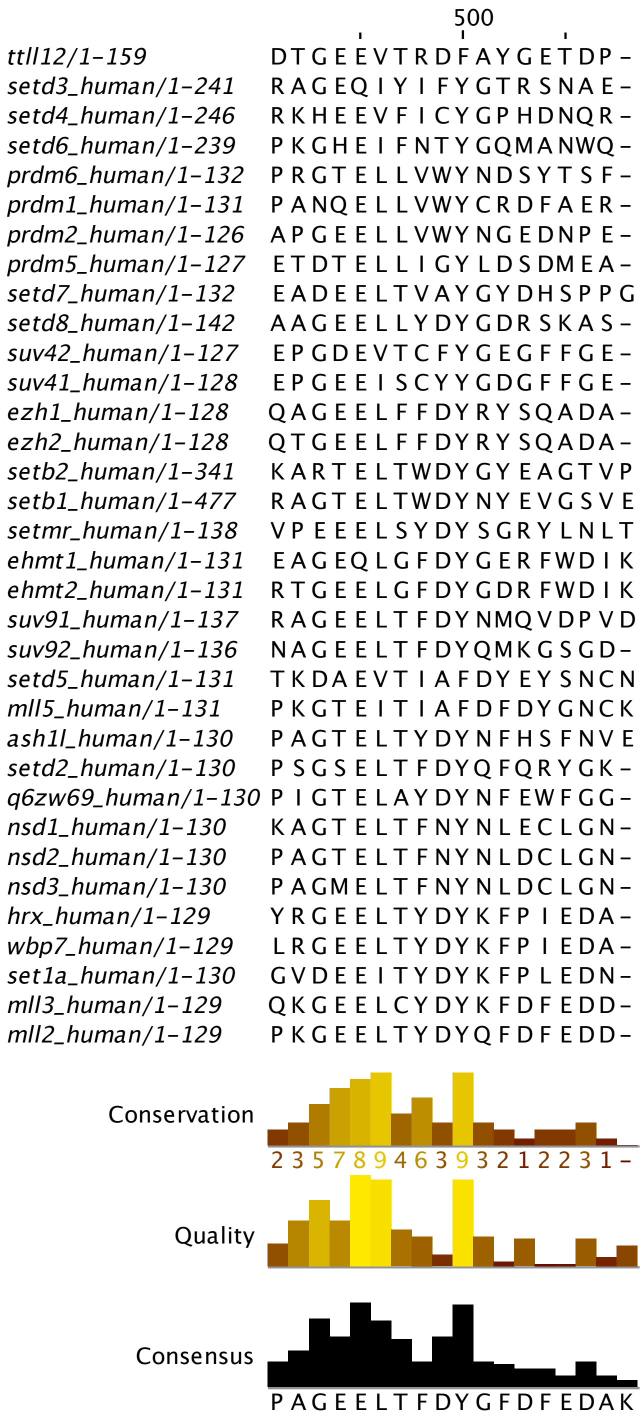
500
ttll12/1-159 DTGEEVTRDFAYGETDP-
setd3_human/1-241 RAGEQIYIFYGTRSNAE-
setd4_human/1-246 RKHEEVFICYGPHDNQR-
setd6_human/1-239 PKGHEIFNTYGQMANWQ-
prdm6_human/1-132 PRGTELLVWYNDSYTSF-
prdm1_human/1-131 PANQELLVWYCRDFAER-
prdm2_human/1-126 APGEELLVWYNGEDNPE-
prdm5_human/1-127 ETDTELLIGYLDSDMEA-
setd7_human/1-132 EADEELTVAYGYDHSPPG
setd8_human/1-142 AAGEELLYDYGDRSKAS-
suv42_human/1-127 EPGDEVTCFYGEGFFGE-
suv41_human/1-128 EPGEEISCYYGDGFFGE-
ezh1_human/1-128 QAGEELFFDYRYSQADA-
ezh2_human/1-128 QTGEELFFDYRYSQADA-
setb2_human/1-341 KARTELTWDYGYEAGTVP
setb1_human/1-477 RAGTELTWDYNYEVGSVE
setmr_human/1-138 VPEEELSYDYSGRYLNLT
ehmt1_human/1-131 EAGEQLGFDYGERFWDIK
ehmt2_human/1-131 RTGEELGFDYGDRFWDIK
suv91_human/1-137 RAGEELTFDYNMQVDPVD
suv92_human/1-136 NAGEELTFDYQMKGSGD-
setd5_human/1-131 TKDAEVTIAFDYEYSNCN
mll5_human/1-131 PKGTEITIAFDFDYGNCK
ash1l_human/1-130 PAGTELTYDYNFHSFNVE
setd2_human/1-130 PSGSELTFDYQFQRYGK-
q6zw69_human/1-130 PIGTELAYDYNFEWFGG-
nsd1_human/1-130 KAGTELTFNYNLECLGN-
nsd2_human/1-130 PAGTELTFNYNLDCLGN-
nsd3_human/1-130 PAGMELTFNYNLDCLGN-
hrx_human/1-129 YRGEELTYDYKFPIEDA-
wbp7_human/1-129 LRGEELTYDYKFPIEDA-
set1a_human/1-130 GVDEEITYDYKFPLEDN-
mll3_human/1-129 QKGEELCYDYKFDFEDD-
mll2_human/1-129 PKGEELTYDYQFDFEDD-
Conservation
2 3 5 7 8 9 4 6 3 9 3 2 1 2 2 3 1 -
Quality
Consensus
PAGEELTFDYGFDFEDAK
